# Regioselective access to polycyclic N-heterocycles via homogeneous copper-catalyzed cascade cyclization of allenynes

**DOI:** 10.1038/s42004-023-00910-9

**Published:** 2023-05-31

**Authors:** Kua-Fei Wei, Qing Liu, Guang Ma, Xiao-Lei Jiang, Xiu-Hong Zhu, Guang-Xin Ru, Wen-Bo Shen

**Affiliations:** College of Sciences and College of Forestry, Henan Agricultural University, Zhengzhou, 450002 China

**Keywords:** Synthetic chemistry methodology, Reaction mechanisms, Homogeneous catalysis, Asymmetric synthesis

## Abstract

Polycyclic N-heterocycles are important structural motifs commonly found in bioactive compounds, however, their selective construction via the cyclization of allenynes remains challenging yet highly desirable. Here we show a homogeneous copper-catalyzed hetero Diels−Alder (HDA) reaction of allenynes with cis-diazenes (PTAD, 4-phenyl-1,2,4-triazoline-3,5-dione), allowing the practical and efficient synthesis of a diverse array of valuable polycyclic N-heterocycles. A temperature-controlled and stereocontrolled chemoselectivity of the reaction was observed, leading to the chemodivergent synthesis of tetracyclic pyrrolidines, pentacyclic triazepanes and tricyclic pyrrolidines. Compared with related Au-catalyzed cyclization of allenynes, this copper catalysis achieves cyclization of allenynes terminating in C–N bond formation via the HDA reaction.

## Abstract (web summary)

Polycyclic *N*-heterocycles are important structural motifs commonly found in bioactive compounds, however, their selective construction *via* the cyclization of allenynes remains challenging. Here, the authors develop a copper-catalyzed, temperature-controlled regioselective *hetero* Diels-Alder reaction of allenynes with *cis* diazenes to produce a diverse array of polycyclic *N*-heterocycles.

## Introduction

Heterocycles containing C–N bonds are particularly significant structural motifs in organic molecules^1–7^. The structurally multiple and interesting family of polycyclic N-heterocycles, such as triazolo[1,2-a]pyridazines^8–11^, pyrrolo[2,3-d][1,2,4]triazolo[1,2-a]pyridazine^12–14^, and pyrrolo[2,3-d][1,2,4]triazolo[1,2-a]pyridazine^15^, are important structural cores that can be regularly found in bioactive natural products as well as in drug candidates. The preparation of structurally diverse heterocycles is important to the discovery of pharmaceutical lead compounds. The synthesis of these skeletons, however, always demands cumbrous synthetic pathways, which is usually time-consuming and labour-intensive. Thus, a practical and efficient strategy capable of selectively forming diverse valuable N-heterocycles from common starting materials, especially those based on assembling structures directly from readily available and easily varied building blocks, are of high value.

**Fig. 1:**
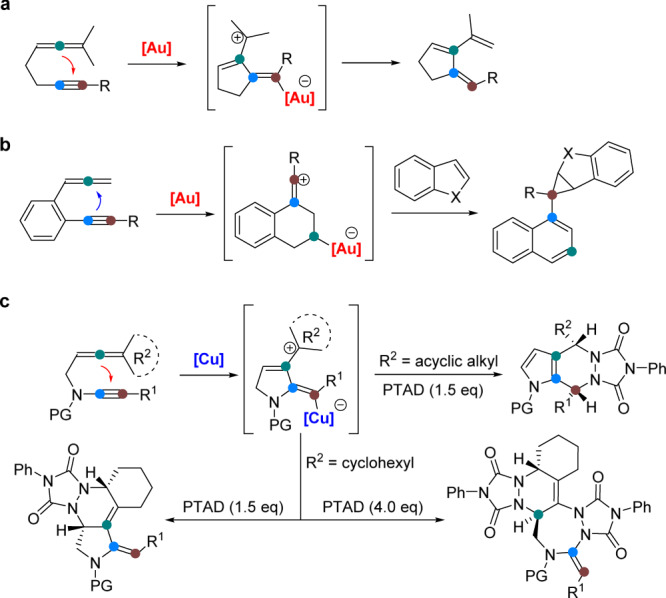
Transition-metal-catalyzed cascade cyclization of allenynes. **a** Gold-catalyzed allenynes intramolecular cyclization via allylic cation. **b** Gold-catalyzed allenynes intermolecular cyclization via vinyl cation. **c** Copper-catalyzed allenynes intermolecular cyclization via allylic cation (this work).

Recently, gold-catalyzed cascade cyclization reaction of alkynes, which have proven to be fundamental synthons in organic synthesis, has received enormous attention, and been widely used in the facile synthesis of a diverse array of the valuable cyclic compounds^16–21^. The gold-activated alkyne can be trapped by heteronucleophiles, such as nitrogen, oxygen, or sulfur derivatives, leading to the formation of heterocycles^18,22–27^. For example, generating α-oxo gold carbenes via oxidation of alkynes, as pioneered by the Zhang group^28,29^, has drawn considerable attention. The generation of α-imino gold carbenes via amination of alkynes have been widely applied to various catalytic reactions^30–34^. Besides, alkynes can also undergo various types of carbocyclizations, including [2 *+* 2]-^35,36^, [3 *+* 2]-^37–39^, [4 *+* 2]-^40–42^, and [2 *+* 2 *+* 2]-type reactions^43–45^, affording the structurally complex carbocycles. However, the gold-catalyzed cyclization of allenynes has been explored relatively seldom as chemo-, regio-, and stereoselectivity are significant. Realizing this tandem reaction is highly challenging due to two competing reactions. First, the allene functions as a nucleophile attacks Au-activated alkyne to form an allylic cation species. In this context, Toste reported an elegant protocol for the gold-catalyzed cycloisomerization of allenynes, and the resultant allylic cation species were terminated with a 1,5-hydrogen shift (Fig. 1a)^46–49^. Second, the alkyne functions as a nucleophile attacks Au-activated allene to generate a vinyl cation species^50,51^. In this case, Ohno et al. disclosed the gold(I)-catalyzed cyclization of allenynes terminating in C–C bond formation via the vinyl cation species (Fig. 1b)^50^. Despite these findings, the formed allylic cation or vinyl cation are terminated by deprotonation or nucleophilic attack, and the reactions terminated with C–N bond formation via the hetero Diels−Alder (HDA) reactions has not yet been reported. Furthermore, these allenyne cyclizations have been restricted to noble-metal catalysts, which has severely hampered the practical application of this approach. To our knowledge, non-noble-metal-catalyzed allenyne cyclizations to generate allylic cation intermediates has not been documented to date in homogeneous transition-metal catalysis. In our ongoing program of expanding copper catalysis into N-heterocycle synthesis^52–59^, we have developed an efficient synthesis of polycyclic N-heterocycles through copper-catalyzed, temperature-controlled regioselective HDA reaction of allenynes with cis-diazenes (PTAD) (Fig. 1c).

The outcome of the process depends on the reaction temperature, with tetracyclic pyrrolidines obtained at room temperature and pentacyclic triazepanes formed at 60 °C. Moreover, this method allows the stereocontrolled synthesis of valuable tricyclic pyrrolidines from an acyclic monosubstituted allenyne. Of note, in all the cases, no intramolecular products were detected^46–49^. The chemoselective HDA, and the high synthetic value of the heterocycles, are of great significance. Herein, the modular construction of synthetically valuable polycyclic N-heterocycles through homogeneous copper-catalyzed hetero Diels−Alder reactions of allenynes with PTAD have been described.

## Results and discussion

### Optimization of the reaction conditions

We started by employing allenyne **1a** as the model substrate and PTAD **2a** as the dienophile, and the initial reaction was run in DCE using Cu(CH_3_CN)_4_PF_6_ as the catalyst. Gratifyingly, the expected tetracyclic pyrrolidine **3a** was indeed obtained in 16% yield (Table 1, entry 1). A subsequent different copper catalysts screening revealed that Cu(OTf)_2_ gave the best result, thus giving the tetracyclic pyrrolidine **3a** in 75% yield (entry 5). We then tried a variety of other Lewis acids, including Zn(OTf)_2_, Y(OTf)_3_, and Sc(OTf)_3_, and general noble metal catalysts such as [Rh(CO)_2_Cl]_2_ and PPh_3_AuCl/AgNTf_2_, but failed to improve the yield (entries 7–11). Interestingly, pentacyclic triazepane **4a** was detected when the reaction was performed at a higher temperature (60 °C) (entry 12). Given the interest of this result, we optimized the reaction conditions towards the selective synthesis of either **3a** or **4a**. To our delight, the reaction was substantially improved when PTAD **2a** (3.0 equiv) was utilized (entry 13). Going a step further, 4.0 equivalents of PTAD **2a** was found to be optimal and the yield of **4a** was increased to 78% (entries 14 and 15). Further raising the reaction temperature failed to improve the reaction (entry 16).

**Table 1:** Optimization of reaction conditions^a^.


Entry	Cat. (*x* mol %)	2a (equiv)	*T* (°C)	Yield (%)^b^	
				3a	4a
1	Cu(CH_3_CN)_4_PF_6_ (10)	1.5	25	16	<1
2	Cu(CH_3_CN)_4_BF_4_ (10)	1.5	25	10	<1
3	CuOTf (10)	1.5	25	8	<1
4	Cu(PPh_3_)_3_Br (10)	1.5	25	<1	<1
5	Cu(OTf)_2_ (10)	1.5	25	75	<1
6	Cu(hfacac)_2_ (10)	1.5	25	<1	<1
7	Zn(OTf)_2_ (10)	1.5	25	15	<1
8	Y(OTf)_3_ (10)	1.5	25	8	<1
9	Sc(OTf)_3_ (10)	1.5	25	46	<1
10	[Rh(CO)_2_Cl]_2_ (5)	1.5	25	<1	<1
11	Ph_3_PAuCl/AgNTf_2_ (5)	1.5	25	<1	<1
12	Cu(OTf)_2_ (10)	1.5	60	41	17
13	Cu(OTf)_2_ (10)	3.0	60	<1	74
14	Cu(OTf)_2_ (10)	4.0	60	<1	78
15	Cu(OTf)_2_ (10)	5.0	60	<1	76
16	Cu(OTf)_2_ (10)	4.0	80	<1	72
17^c^	Cu(OTf)_2_ (10)	4.0	60	<1	81
18^c,d^	Cu(OTf)_2_ (10)	4.0	60	<1	85

^a^Reaction conditions: **1a** (0.1 mmol), **2a** (0.15–0.5 mmol), catalyst (5–10 mol %), 25–80 °C, in vials.

^b^Measured by ^1^H NMR using diethyl phthalate as the internal standard.

^c^The reaction was performed in a flame-dried vial with dry DCE as the solvent.

^d^Using 3 Å molecular sieves (20 mg/0.1 mmol) as an additive.

The temperature turned out to be crucial for the selectivity of this tandem reaction. The chemoselectivity was perfectly controlled via the reaction temperature. Much to our delight, the reaction yield were further improved by using dry DCE as the solvent and 3 Å molecular sieves as an additive (entries 17 and 18).

### Substrate scope for the formation of tetracyclic pyrrolidines 3

**Fig. 2:**
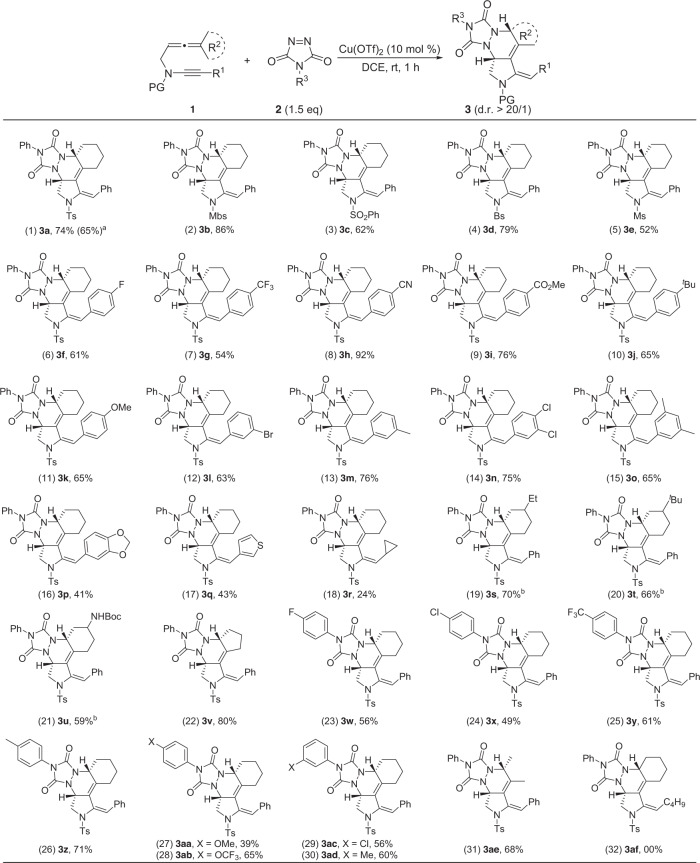
Reaction scope for the formation of tetracyclic pyrrolidines 3. Reaction conditions: [**1**] = 0.05 M; yields are those for the isolated products. **a** The reaction was carried out on a 3.0 mmol scale. **b** The presence of diastereomeric mixtures in the cases of **3s**-**u** could not be ruled out.

With the optimal reaction conditions in hand (Table 1, entry 5), the reaction scope of the copper-catalyzed synthesis of tetracyclic pyrrolidines was then explored (Fig. 2). The copper-catalyzed reaction of allenynes which contain different *N*-protecting groups, such as Ts, Mbs, SO_2_Ph, Bs, and Ms, furnished the expected tetracyclic pyrrolidines **3a**–**e** in 52–86% yields. Molecular structure of **3d** was confirmed by X-ray diffraction^60^. In entries 6–11, this HDA reaction worked smoothly with allenynes bearing p-phenyl substituents including fluoro, tert-butyl, methoxy and even trifluoromethyl, cyano, methyl formate, leading to the formation of tetracyclic pyrrolidines **3f**–**k** in 54–92% yields. Substitutions on the aromatic ring at different sites were easily allowed, thus yielding tetracyclic pyrrolidines **3l**–**p** in 41–76% yields. The reaction was also extended to the thienyl- and cyclopropyl-substituted allenynes to deliver tetracyclic pyrrolidines **3q** and **3r** in 43 and 24% yields, respectively. As shown in the cases of **3s**–**u**, substitutions such as Et, ^t^Bu, and even NHBoc, on the cyclohexane ring were all smoothly accommodated. In addition, cyclopentyl-substituted allenyne **1** **v** was also a suitable substrate for this HDA reaction to give the desired tetracyclic pyrrolidine **3** **v** in 80% yield. We next extended the reaction to a range of PTAD. To our satisfaction, various aryl-substituted PTAD were compatible with this cycloaddition, which delivered the expected tetracyclic pyrrolidines **3w**–**ad** in 39–71% yields. With regard to R^2^ – dimethyl, the corresponding product **3ae** could be obtained in 68% yield. However, with regard to R^1^ – alkyl, the corresponding product **3af** could not be obtained. Importantly, excellent diastereoselectivities (d.r. > 20/1) and unique regioselectivity were achieved in all cases.

### Substrate scope for the formation of pentacyclic triazepanes 4

**Fig. 3:**
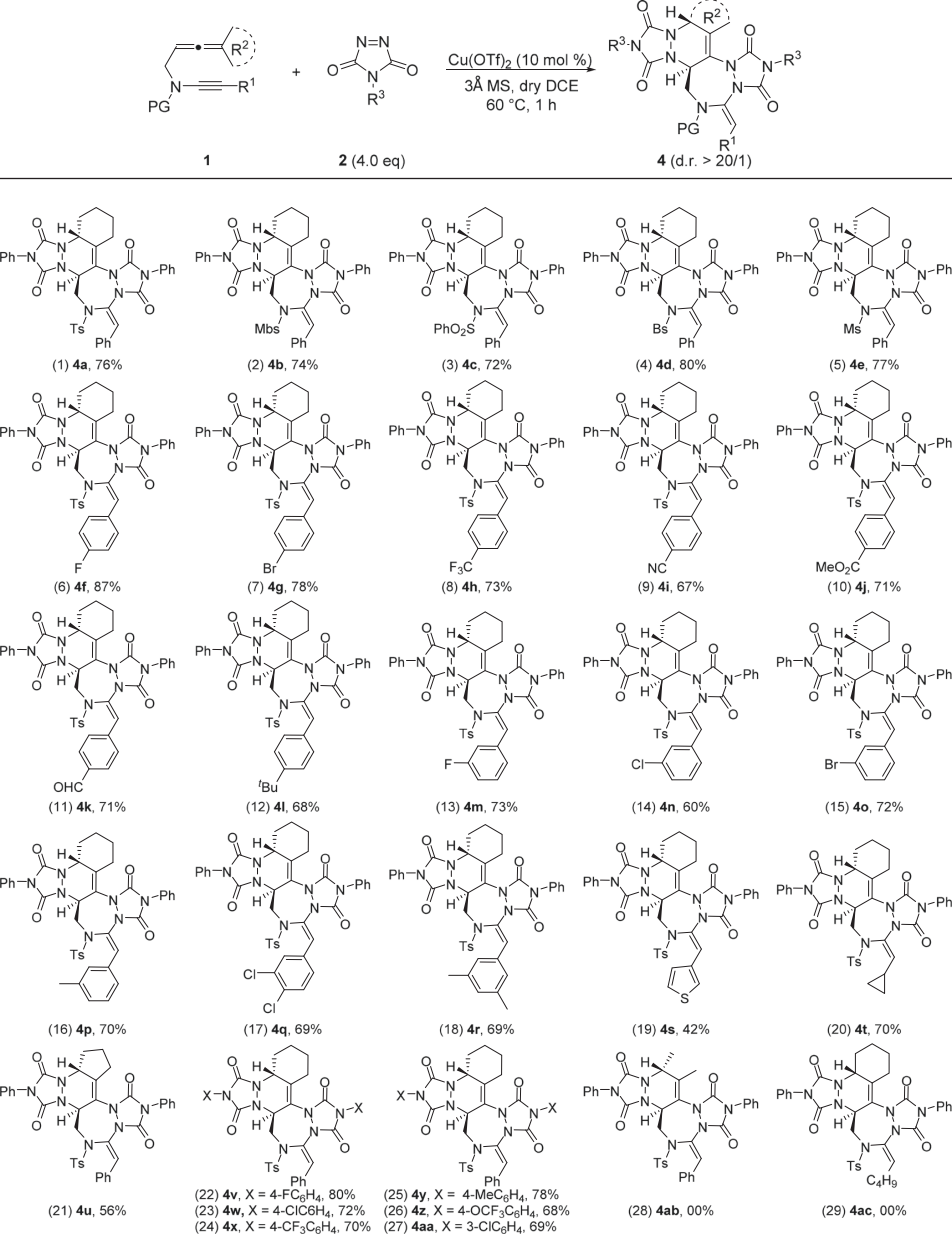
Reaction scope for the formation of pentacyclic triazepanes 4. Reaction conditions: [**1**] = 0.05 M; yields are those for the isolated products.

We also probed the substrate scope for the copper-catalyzed synthesis of pentacyclic triazepanes with the same allenynes substrates under the optimal reaction conditions (Table 1, entry 18). As shown in Fig. 3, allenynes bearing different protecting groups (Ts, Mbs, SO_2_Ph, Bs, and Ms) on nitrogen reacted quite well with PTAD **2a**, providing the pentacyclic triazepanes **4a**–**e** in 72–80% yields. Molecular structure of **4e** was confirmed by X-ray diffraction^60^. Various substituted allenynes were then examined. Allenynes with either electron-withdrawing (F, Br, and even CF_3_, CN, CO_2_Me, CHO) or electron-donating substituents attached to the para position of the phenyl ring proceeded successfully to provide the pentacyclic triazepanes **4f**–**l** in 67–87% yields. Allenynes with varied phenyl substituents including fluoro, chloro, bromo, and methyl substituents at the 3-position were also suitable substrates to give the corresponding products **4m**–**p** in 60–73% yields. The reaction was also applicable to the 3,4-dichloro-substituted and 3,5-dimethyl-substituted allenynes, which was converted into pentacyclic triazepanes **4q** and **4r** in 69% yields. The heteroaromatic thienyl and alkyl substituent were well tolerated in this reaction, affording the pentacyclic triazepanes **4s** and **4t** in 42 and 70% yields, respectively. Interestingly, cyclopentyl-substituted allenyne **1u** was also suitable for this reaction, and afforded the expected pentacyclic triazepane **4u** in 56% yield. Furthermore, various substituted PTAD reacted satisfactorily, furnishing the resulting tetracyclic pyrrolidines **4v**–**aa** in 68–80% yields. With regard to R^2^ – dimethyl, the corresponding product **4ab** could not be obtained. With regard to R^1^ – alkyl, the corresponding product **4ac** could not be obtained.

### Substrate scope for the formation of tricyclic pyrrolidines 6

**Fig. 4:**
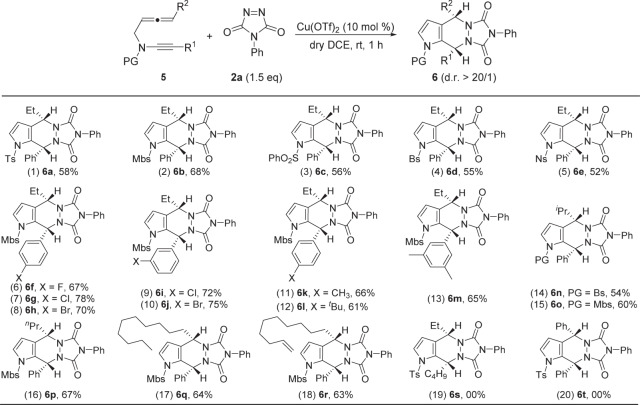
Reaction scope for the formation of tricyclic pyrrolidines 6. Reaction conditions: [**5**] = 0.05 M; yields are those for the isolated products.

We then considered the possibility of extending the above HDA reaction to acyclic monosubstituted allenynes. This HDA reaction proceeded smoothly in the presence of 10 mol% Cu(OTf)_2_ as the catalyst for various acyclic monosubstituted allenynes **5**, and the tricyclic pyrrolidines **6** were obtained in generally moderate to good yields with excellent diastereoselectivity. As shown in Fig. 4, this procedure worked well with a range of acyclic monosubstituted allenynes, and the yields ranged from 52 to 78%. Molecular structure of **6n** was confirmed by X-ray diffraction^60^. With regard to R^1^ - alkyl and R^2^ - phenyl, the corresponding products **6** **s** and **6t** could not be obtained. The newly formed configuration of tertiary carbon centers were consistent with the routine Diels−Alder reaction mechanism that the PTAD approached from the less hindered face. The regioselectivity of [4 + 2] cycloaddition within the key Cu-containing all-carbon 1,4-dipole intermediate is dominated by steric effects (For details, see the **ESI**).

### Experimental expansion

**Fig. 5:**
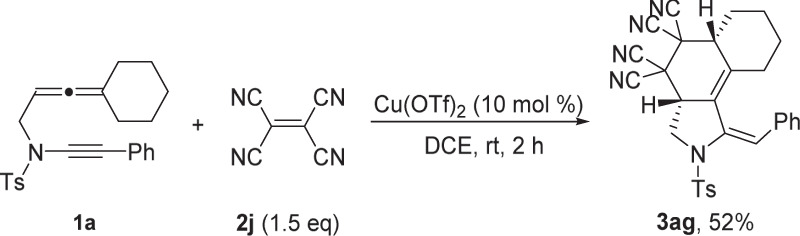
Experimental expansion. Tetracyanoethylene was used as the dienophile.

Besides, this cycloaddition chemistry is not restricted to highly reactive cyclic diazenes. When tetracyanoethylene **2j** as the dienophile, and the reaction was examined in DCE using Cu(OTf)_2_ as the catalyst, the corresponding benzo[e]isoindole **3ag** was also obtained in 52% yield (Fig. 5).

### Synthetic application

**Fig. 6:**
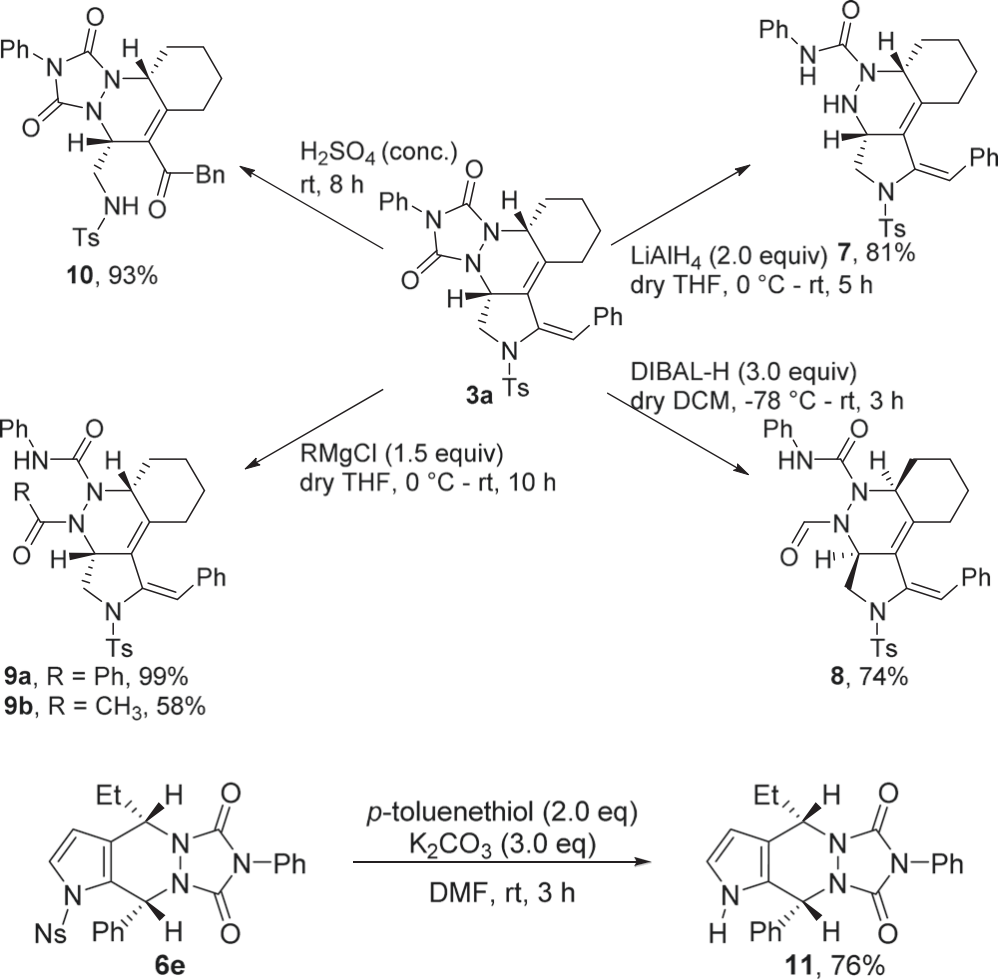
Synthetic applications. The synthetic utility of the **3a** and **6e** were examined.

Further synthetic transformation of the as-synthesized products were investigated (Fig. 6). The compound **3a** could be easily transformed into the resulting products **7** and **8** in 81 and 74% yields, respectively, by treatment with LiAlH_4_ and DIBAL-H. Furthermore, the reaction of **3a** with Grignard reagents resulted in the formation of **9** in 58–99% yields. The C=C double bond of the **3a** could be oxidized by treatment with H_2_SO_4_ to deliver the observed product **10** in almost quantitative yield. The molecular structures of **8,** **9b** and **10** were confirmed by X-ray diffraction^60^. In addition, the Ns group in tricyclic pyrrolidine **6a**, was easily removed by the treatment with p-toluenethiol and K_2_CO_3_ to provide the resulting **11** in 76% yield.

### Mechanistic studies

**Fig. 7:**
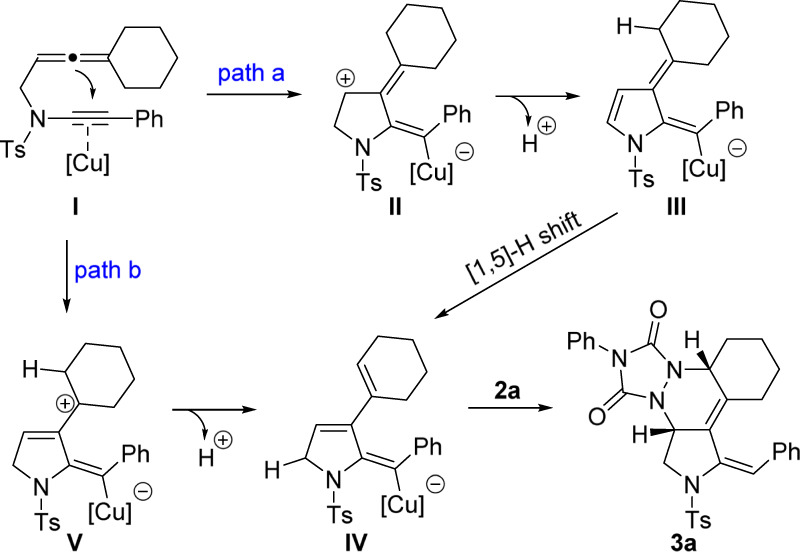
Control experiments. Original mechanism proposed for the formation of product **3a**.

On the basis of previous studies on transition-metal-catalyzed allenyne cyclization^61^, two plausible mechanisms to rationalize the synthesis of tetracyclic pyrrolidine **3a** are presented (Fig. 7). In path a, the reaction commences with attacking of allene moiety to the copper-activated triple bond of electron-rich ynamide moiety, giving the endocyclic allylic cation **II**. Subsequent deprotonation and [1,5]-H shift generates copper-containing diene **IV**, followed by reacting with PTAD **2a**, via HDA reaction, to yield the tetracyclic pyrrolidine **3a**. In path b, exocyclic allylic cation **V** can be generated, further producing the expected copper-containing diene **IV** upon a loss of a proton.

**Fig. 8:**
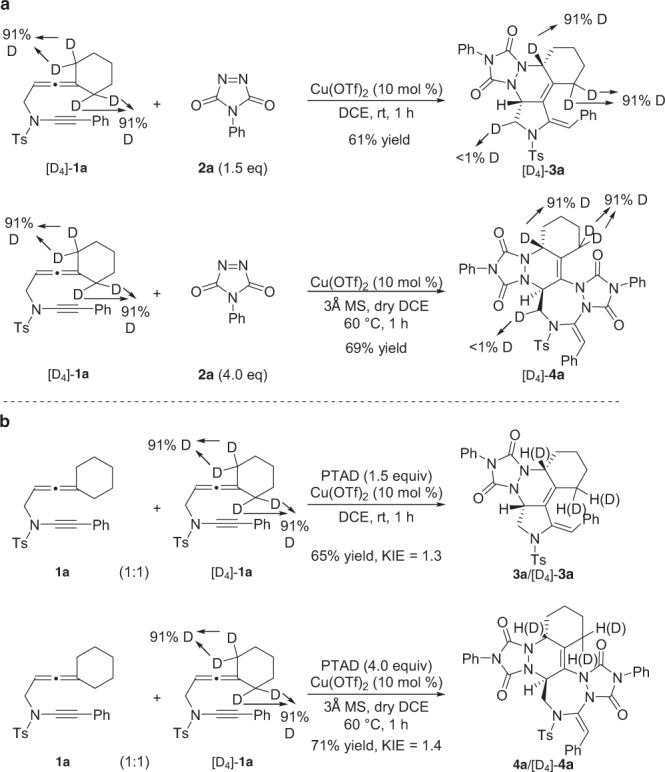
Deuterium labeling experiment. **a** The reaction of [D_4_]-**1a** under the standard conditions. **b** KIE experiment.

To further understand the mechanism of the allenyne cyclizations, in particular accounting for the distinct selectivity, several control experiments were explored. First, deuterium labeling experiment was examined with the allenyne [D_4_]-**1a**. It was found that <1% deuterium incorporation into the pyrrole ring partner of the desired product was detected when starting from the deuterium-labelled substrate [D_4_]-**1a** (91% D), suggesting that path a of Scheme 3 was unlikely, as hydride shift should occur in the formation of copper-containing diene **IV** (Fig. 8a). Besides, kinetic isotope effect (KIE) studies were also performed with the mixture of allenyne **1a** and [D_4_]-**1a**, and the KIE data indicated that the cleavage of methylene C(sp^3^)–H bond was not the rate-determining step (Fig. 8b).

### Proposed mechanism

**Fig. 9:**
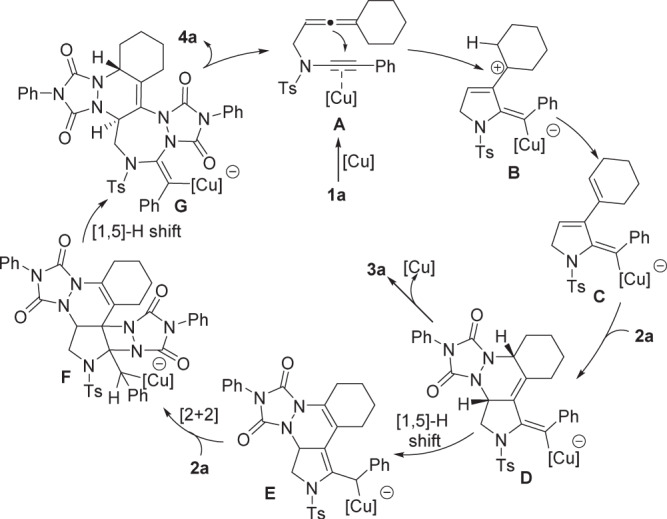
Reaction mechanism. Plausible reaction mechanism for the formation of **3a** and **4a**.

Based on the above experimental observations, a plausible mechanism to rationalize the formation of **3a** and **4a** are presented (Fig. 9). The electron-rich alkyne moiety of **1a** is activated by a cationic copper catalyst to afford copper complex **A**, followed by intramolecular nucleophilic attack by the allene to generate allylic cation intermediate **B**. The allylic cation **B** undergoes a loss of a proton to form copper-containing diene **C**, followed by reacting with PTAD, via HDA reaction, to deliver the tetracyclic pyrrolidine **3a** through vinyl copper intermediate **D**^61^. Alternatively, [1,5]-H shift may occur and then form the intermediate **E**, further reacting with PTAD, via [2 + 2] cycloaddition, to provide the hexacyclic pyrrolidine intermediate **F**. Subsequent [1,5]-H migration and C–C cleavage allow the formation of the pentacyclic triazepane **4a** along with the release of ring strain. The newly generated configuration of tertiary carbon centers (**3a**) supports the Diels−Alder reaction mechanism that the PTAD approaches from the less hindered face. The formed configuration of tertiary carbon centers (**4a**) suggests that hydride shift was presumably involved in the formation of seven-membered ring moiety.

## Conclusions

In summary, we have developed the modular construction of synthetically valuable polycyclic N-heterocycles through homogeneous copper-catalyzed hetero Diels−Alder reactions of allenynes with PTAD. This reaction shows excellent diastereoselectivities in the ring-formation step and features a broad substrate scope. It is worth noting that the outcome of the procedure can be controlled by adjusting the temperature of the reaction system, giving access to two different N-heterocycle skeletons, namely tetracyclic pyrrolidines and pentacyclic triazepanes, from the same starting materials. This method enables efficient and stereocontrolled access to valuable tricyclic pyrrolidines under mild reaction conditions from an acyclic monosubstituted allenyne. Compared with related Au-catalyzed cyclization of allenynes, this copper catalysis achieves cyclization of allenynes terminating in C–N bond formation via the chemoselective HDA, to the best of our knowledge, which represents the first non-noble metal-catalyzed allenyne cyclizations to generate allylic cation intermediates in homogeneous transition-metal catalysis. Moreover, copper catalysis may be advantageous in catalytic asymmetric reactions over gold catalysis due to the linear coordination favored by gold. The development of copper-catalyzed asymmetric cascade cyclization of allenynes and mechanistic explorations are the themes of continuing study in our laboratory.

## Methods

### Materials

Unless otherwise noted, materials were obtained commercially and used without further purification. All the solvents were treated according to general methods. Flash column chromatography was performed over silica gel (300–400 mesh). See Supplementary Methods for experimental details.

### General methods

^1^H NMR spectra were recorded on a Bruker AV-400 spectrometer in chloroform-d_3_. Chemical shifts are reported in ppm with the internal TMS signal at 0.0 ppm as a standard. The data is being reported as (s = singlet, d = doublet, t = triplet, m = multiplet or unresolved, brs = broad singlet, coupling constant(s) in Hz, integration). ^13^C NMR spectra were recorded on a Bruker AV-400 spectrometer in chloroform-d_3_. Chemical shifts are reported in ppm with the internal chloroform signal at 77.0 ppm as a standard. Infrared spectra were recorded on a Nicolet iS 10 spectrometer as thin film and are reported in reciprocal centimeter (cm^−1^). Mass spectra were recorded with Micromass Q-Exactive Focus mass spectrometer using electron spray ionization. More mechanism studies are supplied: see Supplementary Figs. 89–90. Representative synthetic procedures for the preparation of substrates are supplied: see Supplementary Figs. 91–93. General procedure for the synthesis of tetracyclic pyrrolidines **3** are supplied: see Supplementary Fig. 94. General procedure for the synthesis of pentacyclic triazepanes **4** are supplied: see Supplementary Fig. 95. General procedure for the synthesis of tricyclic pyrrolidines **6** are supplied: see Supplementary Fig. 96. Synthetic applications are supplied: see Supplementary Figs. 97–101. Deuterium labeling experiments are supplied: see Supplementary Figs. 102–106. Crystal datas are supplied: see Supplementary Tables 1–6. See Supplementary Methods for the characterization data of compounds not listed in this part.

### General procedure for the synthesis of tetracyclic pyrrolidines 3

PTAD (cis-diazenes) **2** (0.3 mmol), and Cu(OTf)_2_ (0.02 mmol, 7.2 mg) were added in this order to the allenynes **1** (0.2 mmol) in DCE (4.0 mL) at room temperature. The reaction mixture was stirred at room temperature and the progress of the reaction was monitored by TLC. The reaction typically took 1 h. Upon completion, the mixture was then concentrated and the residue was purified by chromatography on silica gel (eluent: petroleum ether/ethyl acetate) to afford the desired products **3**.

### General procedure for the synthesis of pentacyclic triazepanes 4

PTAD (cis-diazenes) **2** (0.8 mmol), 3 Å molecular sieves (40 mg), and Cu(OTf)_2_ (0.02 mmol, 7.2 mg) were added in this order to the allenynes **1** (0.2 mmol) in dry DCE (4.0 mL) at room temperature. The reaction mixture was stirred at 60 °C (60 °C, heating mantle temperature) and the progress of the reaction was monitored by TLC. The reaction typically took 1 h. Upon completion, the mixture was then concentrated and the residue was purified by chromatography on silica gel (eluent: petroleum ether/ethyl acetate) to afford the desired products **4**.

### General procedure for the synthesis of tricyclic pyrrolidines 6

PTAD (cis-diazenes) **2a** (0.3 mmol, 52.5 mg), and Cu(OTf)_2_ (0.02 mmol, 7.2 mg) were added in this order to the allenynes **5** (0.2 mmol) in dry DCE (8.0 mL) at room temperature. Under N_2_ atmosphere, the reaction mixture was stirred at room temperature and the progress of the reaction was monitored by TLC. The reaction typically took 1 h. Upon completion, the mixture was then concentrated and the residue was purified by chromatography on silica gel (eluent: petroleum ether/ethyl acetate) to afford the desired products **6**.

## Supplementary information

Supplementary Information

Description of Additional Supplementary Files

## Data Availability

The authors declare that the data supporting the findings of this study are available within the article and the Supplementary Information as well as from the authors upon reasonable request. The compound characterizations are available in Supplementary Data 1. ^1^H NMR, and ^13^C NMR are supplied for all compounds: see Supplementary Figs. 1–88. The X-ray crystallographic coordinates for structures **3d**, **4e**, **6n**, **8**, **9b**, and **10**, reported in this study have been deposited at the Cambridge Crystallographic Data Centre (CCDC), under CCDC 2205946 (**3d**, Supplementary Data 2), 2192313 (**4e**, Supplementary Data 3), 2192314 (**6n**, Supplementary Data 4), 2192316 (**8**, Supplementary Data 5), 2192317 (**9b**, Supplementary Data 6) and 2192318 (**10**, Supplementary Data 7), respectively. These data can be obtained free of charge from The Cambridge Crystallographic Data Centre via http://www.ccdc.cam.ac.uk/data_request/cif.
