# Supplementary material for: Regioselective access to polycyclic N-heterocycles via homogeneous copper-catalyzed cascade cyclization of allenynes
Source: Commun Chem. 2023 May 31;6:104. doi: 10.1038/s42004-023-00910-9 (PMC10232412; doi:10.1038/s42004-023-00910-9)
Supplement: Supplementary file 1 — Supplementary Information [file 42004_2023_910_MOESM1_ESM.pdf]

Supplementary Table 1. Crystal data and structure refinement for 3d. CCDC Number = 2205946

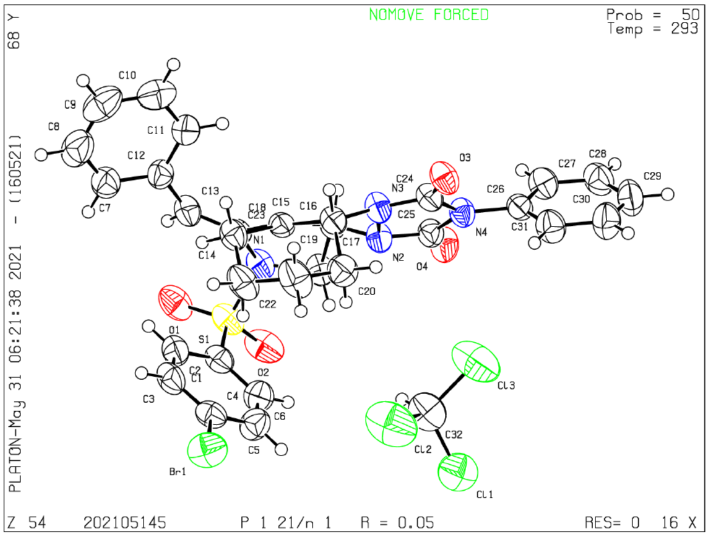

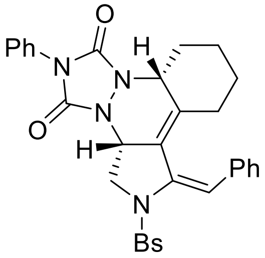

Bond precision:	C-C = 0.0062 A		Wavelength=1.54184
Cell:	a=9.8081(6)	b=28.9644(16)	c=12.7092(10)
	alpha=90	beta=111.895(8)	gamma=90
Temperature:	293 K		

	Calculated	Reported
Volume	3350.1(4)	3350.1(4)
Space group	P 21/n	P 1 21/n 1
Hall group	-P 2yn	-P 2yn
Moiety formula	C31 H27 Br N4 O4 S, C H Cl3	C31 H27 Br N4 O4 S, C H Cl3
Sum formula	C32 H28 Br Cl3 N4 O4 S	C32 H28 Br Cl3 N4 O4 S
Mr	750.89	750.90
Dx,g cm-3	1.489	1.489
Z	4	4
Mu (mm-1)	4.811	4.811
F000	1528.0	1528.0
F000’	1534.47	
h,k,lmax	11,34,15	11,34,15
Nref	5993	5986
Tmin,Tmax	0.525,0.618	0.899,1.000
Tmin’	0.441	

Correction method= # Reported T Limits: Tmin=0.899 Tmax=1.000
AbsCorr = MULTI-SCAN

Data completeness= 0.999 Theta(max)= 67.079

R(reflections)= 0.0514(4421) wR2(reflections)= 0.1488(5986)

S = 1.063 Npar= 406

Supplementary Table 2. Crystal data and structure refinement for 4e. CCDC Number = 2192313

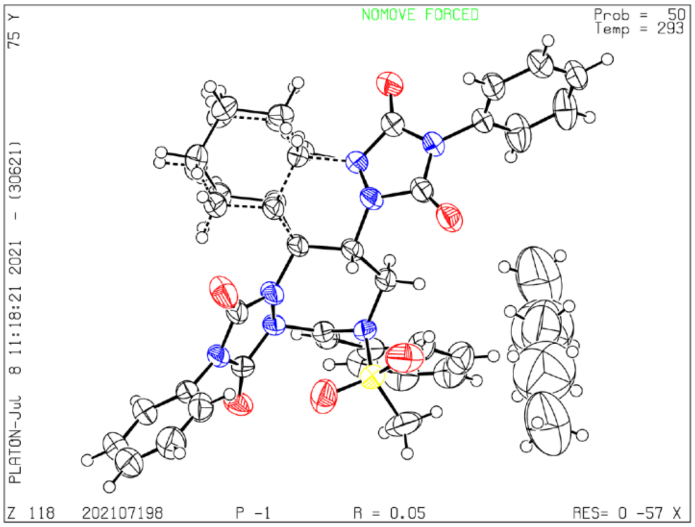

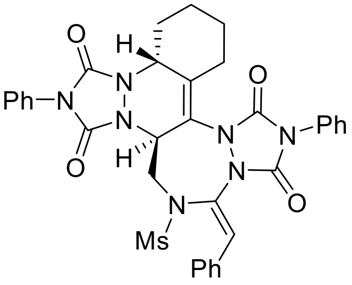

Bond precision: C-C = 0.0062 A Wavelength=1.54184

Cell: a=9.9395(10) b=13.2124(15) c=14.8336(14)
alpha=65.975(10) beta=81.500(8) gamma=89.806(9)

Temperature: 293 K

	Calculated	Reported
Volume	1756.2(3)	1756.2(3)
Space group	P -1	P -1
Hall group	-P 1	-P 1
Moiety formula	C34 H31 N7 O6 S, 0.5(C6 H14)	C34 H31 N7 O6 S, C3 H7
Sum formula	C37 H38 N7 O6 S	C37 H38 N7 O6 S
Mr	708.80	708.80
Dx,g cm-3	1.340	1.340
Z	2	2
Mu (mm-1)	1.293	1.293
F000	746.0	746.0
F000’	748.89	
h,k,lmax	12,16,18	12,16,18
Nref	6778	6615
Tmin,Tmax	0.856,0.925	0.854,1.000
Tmin’	0.834	

Correction method= # Reported T Limits: Tmin=0.854 Tmax=1.000
AbsCorr = MULTI-SCAN

Data completeness= 0.976 Theta(max)= 70.954

R(reflections)= 0.0532(4838) wR2(reflections)= 0.1539(6615)

S = 1.023 Npar= 481

Supplementary Table 3. Crystal data and structure refinement for 6n. CCDC Number = 2192314

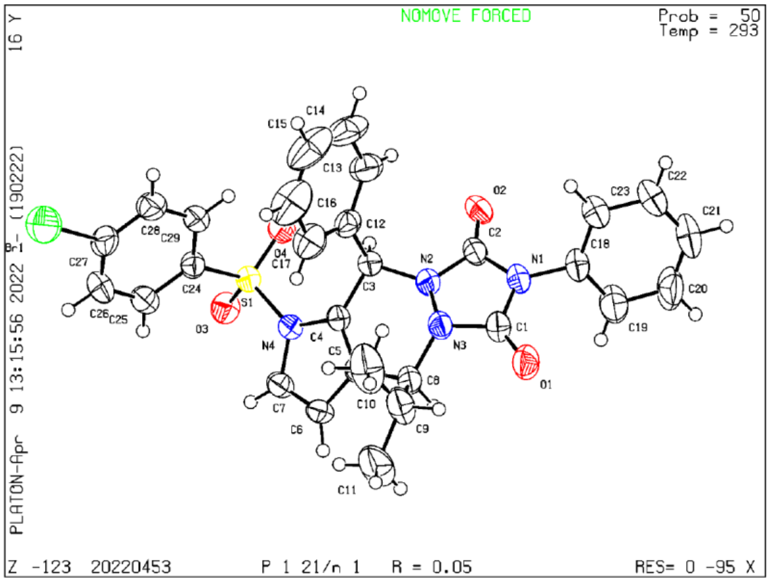

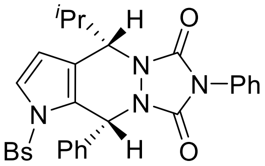

Bond precision: C-C = 0.0060 A Wavelength=1.54184

Cell:	a=10.4466(3)	b=22.3001(6)	c=12.1357(3)
	alpha=90	beta=106.400(3)	gamma=90
Temperature:	293 K		

	Calculated	Reported
Volume	2712.11(13)	2712.10(13)
Space group	P 21/n	P 1 21/n 1
Hall group	-P 2yn	-P 2yn
Moiety formula	C29 H25 Br N4 O4 S	C29 H25 Br N4 O4 S
Sum formula	C29 H25 Br N4 O4 S	C29 H25 Br N4 O4 S
Mr	605.49	605.50
Dx,g cm-3	1.483	1.483
Z	4	4
Mu (mm-1)	3.142	3.142
F000	1240.0	1240.0
F000’	1241.82	
h,k,lmax	12,26,14	12,26,14
Nref	4853	4852
Tmin,Tmax	0.698,0.730	0.789,1.000
Tmin’	0.633	

Correction method= # Reported T Limits: Tmin=0.789 Tmax=1.000
AbsCorr = MULTI-SCAN

Data completeness= 1.000 Theta(max)= 67.074

R(reflections)= 0.0532(3937) wR2(reflections)= 0.1661(4852)

S = 1.032 Npar= 354

Supplementary Table 4. Crystal data and structure refinement for 8. CCDC Number = 2192316

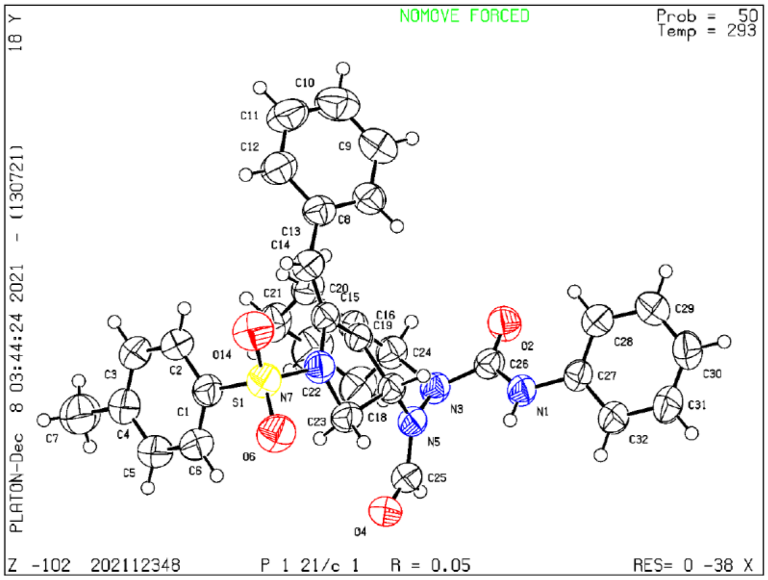

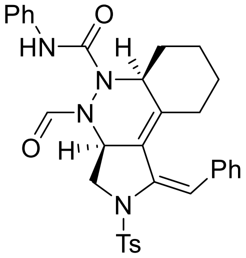

Bond precision: C-C = 0.0037 A Wavelength=1.54184

Cell:	a=12.2897(7)	b=19.6801(9)	c=16.1367(8)
	alpha=90	beta=111.309(6)	gamma=90
Temperature:	293 K		

	Calculated	Reported
Volume	3636.0(4)	3636.0(4)
Space group	P 21/c	P 1 21/c 1
Hall group	-P 2ybc	-P 2ybc
Moiety formula	C32 H32 N4 O4 S [+ solvent]	C32 H32 N4 O4 S
Sum formula	C32 H32 N4 O4 S [+ solvent]	C32 H32 N4 O4 S
Mr	568.68	568.67
Dx,g cm-3	1.039	1.039
Z	4	4
Mu (mm-1)	1.075	1.075
F000	1200.0	1200.0
F000'	1204.74	
h,k,lmax	14,23,19	14,23,19
Nref	6505	6498
Tmin,Tmax	0.846,0.898	0.851,1.000
Tmin'	0.824	

Correction method= # Reported T Limits: Tmin=0.851 Tmax=1.000
AbsCorr = MULTI-SCAN

Data completeness= 0.999 Theta(max)= 67.079

R(reflections)= 0.0486(4713) wR2(reflections)= 0.1454(6498)

S = 1.035 Npar= 375

Supplementary Table 5. Crystal data and structure refinement for 9b. CCDC Number = 2192317

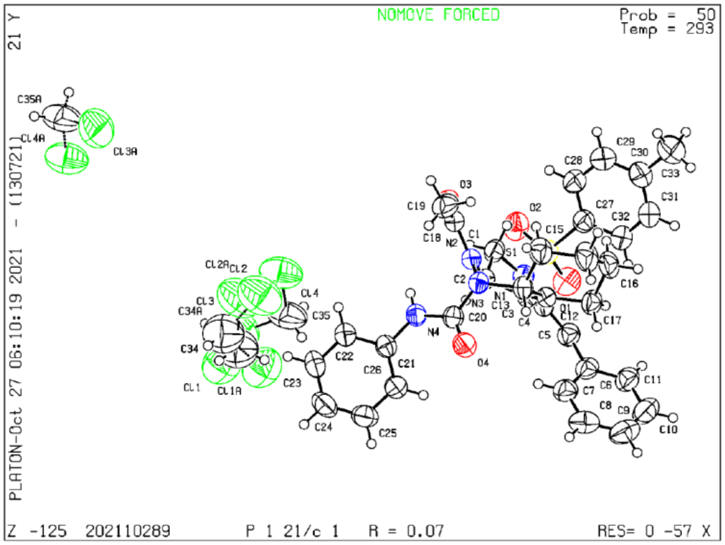

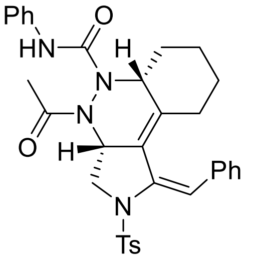

Bond precision: C-C = 0.0052 A Wavelength=1.54184

Cell:	a=12.91883(14)	b=19.00279(19)	c=16.11415(18)
	alpha=90	beta=110.4134(13)	gamma=90
Temperature:	293 K		

	Calculated	Reported
Volume	3707.49(8)	3707.49(7)
Space group	P 21/c	P 1 21/c 1
Hall group	-P 2ybc	-P 2ybc
Moiety formula	C33 H34 N4 O4 S, 2(C H2 Cl2)	C33 H34 N4 O4 S, 2(C1 H2 Cl2)
Sum formula	C35 H38 Cl4 N4 O4 S	C35 H38 Cl4 N4 O4 S
Mr	752.55	752.55
Dx,g cm-3	1.348	1.348
Z	4	4
Mu (mm-1)	3.777	3.777
F000	1568.0	1568.0
F000'	1578.84	
h,k,lmax	15,22,19	15,22,19
Nref	6629	6628
Tmin,Tmax	0.596,0.685	0.893,1.000
Tmin'	0.540	

Correction method= # Reported T Limits: Tmin=0.893 Tmax=1.000
AbsCorr = MULTI-SCAN

Data completeness= 1.000 Theta(max)= 67.071

R(reflections)= 0.0746(5346) wR2(reflections)= 0.2372(6628)

S = 1.047 Npar= 455

Supplementary Table 6. Crystal data and structure refinement for 10. CCDC Number = 2192318

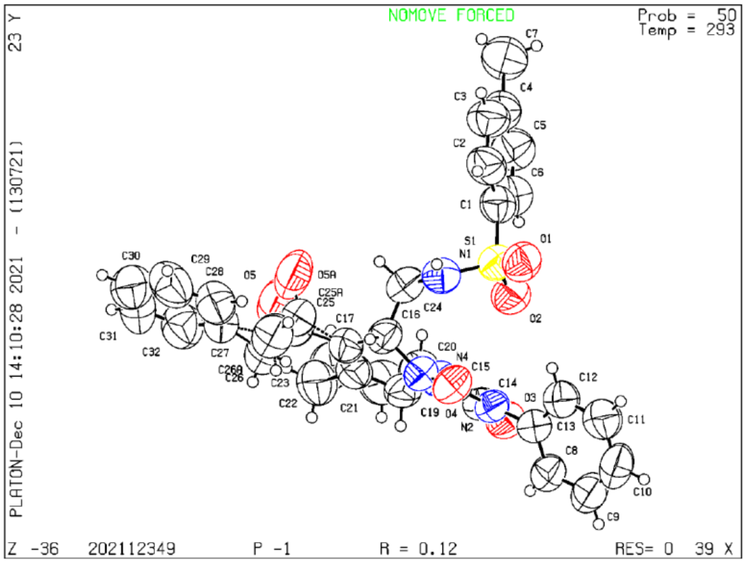

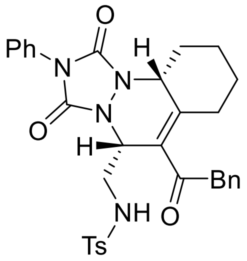

Bond precision: C-C = 0.0095 A Wavelength=1.54184

Cell: a=11.3534(14) b=13.0168(13) c=15.1159(19)
alpha=65.367(11) beta=70.152(12) gamma=70.92(1)

Temperature: 293 K

	Calculated	Reported
Volume	1864.6(4)	1864.6(4)
Space group	P -1	P -1
Hall group	-P 1	-P 1
Moiety formula	C32 H32 N4 O5 S [+ solvent]	C32 H32 N4 O5 S
Sum formula	C32 H32 N4 O5 S [+ solvent]	C32 H32 N4 O5 S
Mr	584.68	584.67
Dx,g cm-3	1.041	1.041
Z	2	2
Mu (mm-1)	1.081	1.081
F000	616.0	616.0
F000'	618.47	
h,k,lmax	13,15,18	13,15,18
Nref	6661	6643
Tmin,Tmax	0.823,0.898	0.542,1.000
Tmin'	0.788	

Correction method= # Reported T Limits: Tmin=0.542 Tmax=1.000
AbsCorr = MULTI-SCAN

Data completeness= 0.997 Theta(max)= 67.078

R(reflections)= 0.1182(2896) wR2(reflections)= 0.3522(6643)

S = 1.040 Npar= 358

Supplementary Discussion

More Mechanism Studies

1. To understand the mechanism of the allenyne cyclizations, several control experiments were explored. First, when tetracyclic pyrrolidine **3a** as the substrate and PTAD as the dienophile, and the reaction was examined in DCE using $Cu(OTf)_2$ as the catalyst, the pentacyclic triazepane **4a** was not detected. **4a** could not be converted into **3a** under Cu catalysis, and only **4a** was recoveried. These results indicated that **3a** and **4a** could not be converted to each other.

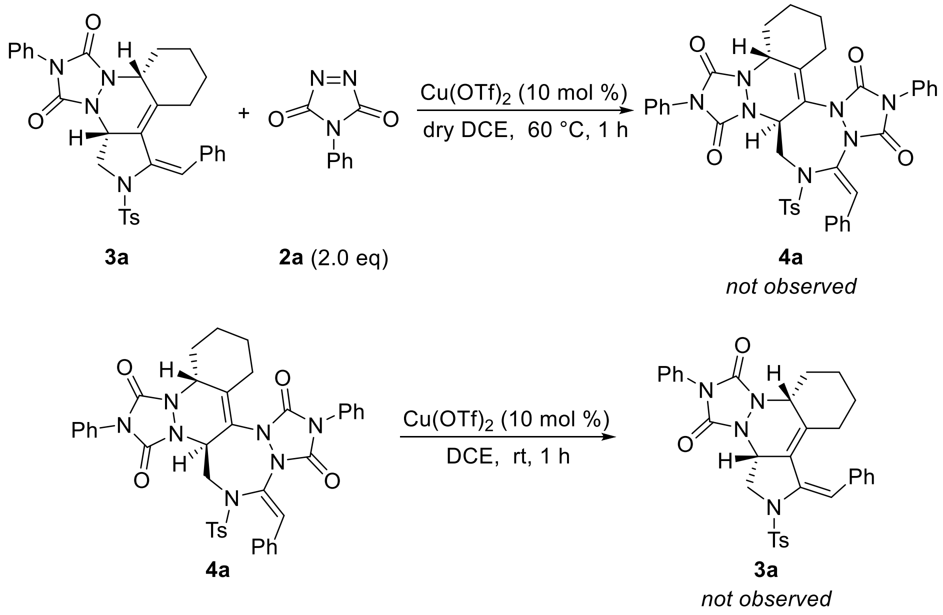

Supplementary Figure 89. Control experiments.

2. Plausible reaction mechanism for the formation of **6**.

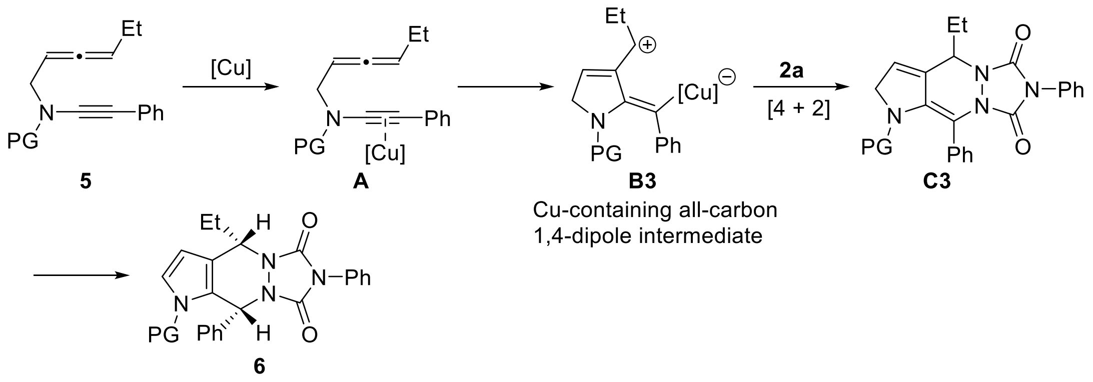

Supplementary Figure 90. Plausible reaction pathway.

Supplementary Methods

General Information. Ethyl acetate (ACS grade), hexanes (ACS grade) and anhydrous 1,2-dichloroethane (ACS grade) were obtained commercially and used without further purification. Methylene chloride, tetrahydrofuran and diethyl ether were purified according to standard methods unless otherwise noted. Commercially available reagents were used without further purification. Reactions were monitored by thin layer chromatography (TLC) using silicycle pre-coated silica gel plates. Flash column chromatography was performed over silica gel (300-400 mesh). Infrared spectra were recorded on a Nicolet iS 10 spectrometer as thin film and are reported in reciprocal centimeter (cm^{-1}). Mass spectra were recorded with Micromass Q-Exactive Focus mass spectrometer using electron spray ionization.

^{1}H NMR spectra were recorded on a Bruker AV-400 spectrometer in chloroform-d_3. Chemical shifts are reported in ppm with the internal TMS signal at 0.0 ppm as a standard. The data is being reported as (s = singlet, d = doublet, t = triplet, m = multiplet or unresolved, brs = broad singlet, coupling constant(s) in Hz, integration).

^{13}C NMR spectra were recorded on a Bruker AV-400 spectrometer in chloroform-d_3. Chemical shifts are reported in ppm with the internal chloroform signal at 77.0 ppm as a standard.

Experimental Section

Representative synthetic procedures for the preparation of allenynes 1a-1u:

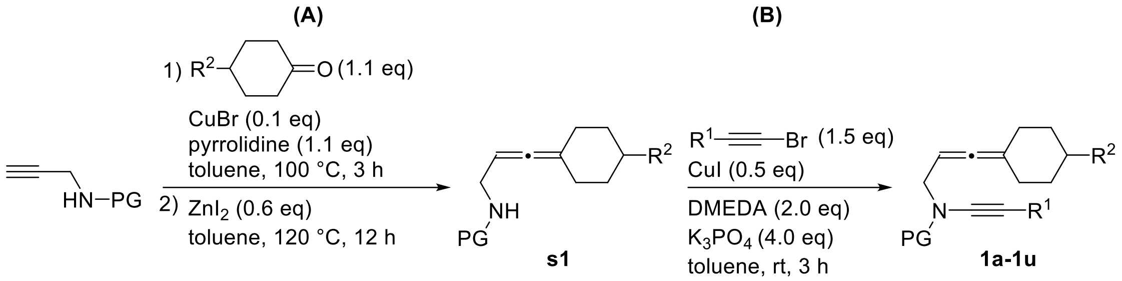

Supplementary Figure 91. Representative synthetic procedures for the preparation of allenynes **1** (**1a-1u**).

(A): *N*-sulfonyl propargyl amine (10.0 mmol), CuBr (1.0 mmol, 143.0 mg) was slowly added to a solution of cyclohexanone (11.0 mmol) in 30.0 mL toluene at room temperature. The reaction mixture was stirred at room temperature for 10 min. Then the pyrrolidine (11.0 mmol, 781.0 mg) was added and the reaction mixture was stirred at 100 °C for 3 h. Upon completion, the reaction crude was filtered through a Celite plug and concentrated under vacuum. Then ZnI_2 (6.0 mmol, 1914.0 mg), toluene (30.0 mL) were added into the residue and the reaction mixture was stirred at 120 °C for 12 h. Upon completion, the reaction crude was filtered through a Celite plug and concentrated under vacuum, and the residue was purified by chromatography on silica gel (eluent: petroleum ether/ethyl acetate) to afford the desired substrates **s1**.[1]

(B): To a mixture of **s1** (3.0 mmol), K_3PO_4 (12.0 mmol, 2544.0 mg), CuI (1.5 mmol, 285.0 mg), and DMEDA (3.0 mmol, 264.0 mg) in the reaction vial was added a solution of a respective brominated alkyne (3.3 mmol) in toluene. The reaction mixture was stirred at room temperature and the progress of the reaction was monitored by TLC. The reaction typically took 3 h. Upon completion, the reaction crude was filtered through a Celite plug and concentrated under vacuum, and the residue was purified by chromatography on silica gel (eluent: petroleum ether/ethyl acetate) to afford the desired allenyne substrates **1a-1u**.[2]

Representative synthetic procedures for the preparation of *cis*-diazenes 2b-2i:

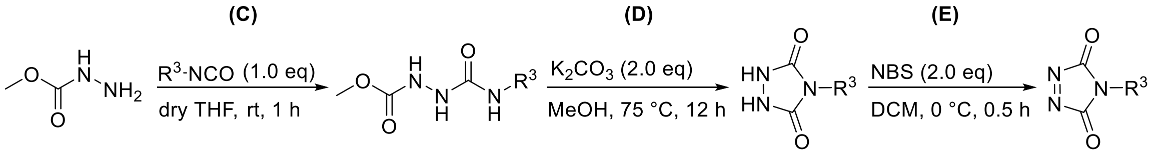

s2 **s3** **2b-2i**

Supplementary Figure 92. Representative synthetic procedures for the preparation of *cis*-diazenes **2** (**2b-2i**).

(C): Aryl isocyante (10.0 mmol) was slowly added to a solution of methyl hydrazinocarboxylate (10.0 mmol, 900.0 mg) in 30.0 mL dry THF at room temperature under argon atmosphere. The reaction mixture was stirred at room temperature for 1 h. Upon completion, the reaction crude was filtered to afford the the white precipitate substrates **s2**.[3]

(D): To a solution of **s2** (10.0 mmol) in MeOH (20 mL) was added potassium carboxylate (20.0 mmol, 2760.0 mg) at room temperature. The reaction mixture was stirred 75 °C and the progress of the reaction was monitored by TLC. The reaction typically took 12 h. Upon completion, the reaction crude was filtered through a Celite plug and concentrated under vacuum, and the residue was purified by chromatography on silica gel (eluent: petroleum ether/ethyl acetate) to afford the substrates **s3**.[3]

(E): To a solution of **s3** (3.0 mmol) in DCM (15 mL) was added NBS (6.0 mmol, 1068.0 mg) at 0°C and the resulting reaction mixture was stirred for 0.5 h. Upon completion, the reaction crude was filtered through a Celite plug and concentrated under vacuum, and the residue was purified by chromatography on silica gel (eluent: petroleum ether/ethyl acetate) to afford the *cis*-diazenes **2b-2i**.[4]

Representative synthetic procedures for the preparation of allenynes 5a-5i:

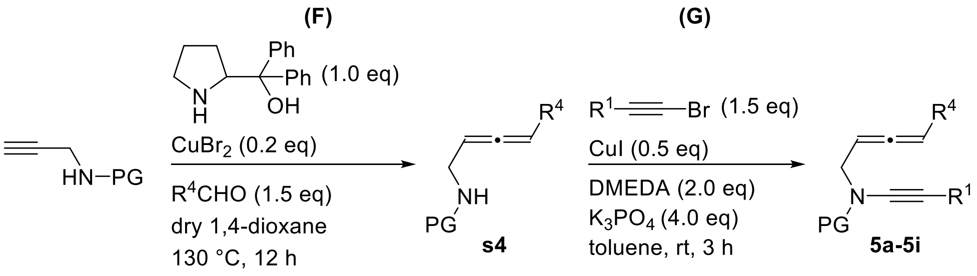

Supplementary Figure 93. Representative synthetic procedures for the preparation of allenynes **5** (**5a-5i**).

(F): To a mixture of aldehyde (3.0 mmol), $CuBr_2$ (0.6 mmol, 133.8 mg), and 2-(diphenylhydroxymethyl) pyrrolidine (3.0 mmol, 759.0 mg) in the reaction vial was added a solution of a respective *N*-sulfonyl propargyl amine (3.0 mmol) in dry 1,4-dioxane (15 mL) at room temperature. The resulting reaction mixture was stirred at 130°C for 12 h. After cooled down to room temperature, the reaction mixture was diluted with diethyl ether (20 ml) and washed with aqueous hydrochloric acid solution (1M, 20 ml). The organic layer was separated, and the aqueous layer was washed with diethyl ether three times (15 ml). The combined organic layer was dried with $MgSO_4$, then filtered and concentrated under vacuum, and the residue was purified by chromatography on silica gel (eluent: petroleum ether/ethyl acetate) to afford the substrates **s4**.[5].

(G): To a mixture of **s4** (3.0 mmol), K_3PO_4 (12.0 mmol, 2544.0 mg), CuI (1.5 mmol, 285.0 mg), and DMEDA (3.0 mmol, 264.0 mg) in the reaction vial was added a solution of a respective brominated alkyne (3.3 mmol) in toluene. The reaction mixture was stirred at room temperature and the progress of the reaction was monitored by TLC. The reaction typically took 3 h. Upon completion, the reaction crude was filtered through a Celite plug and concentrated under vacuum, and the residue was purified by chromatography on silica gel (eluent: petroleum ether/ethyl acetate) to afford the desired allenyne substrates **5a-5i**.[2]

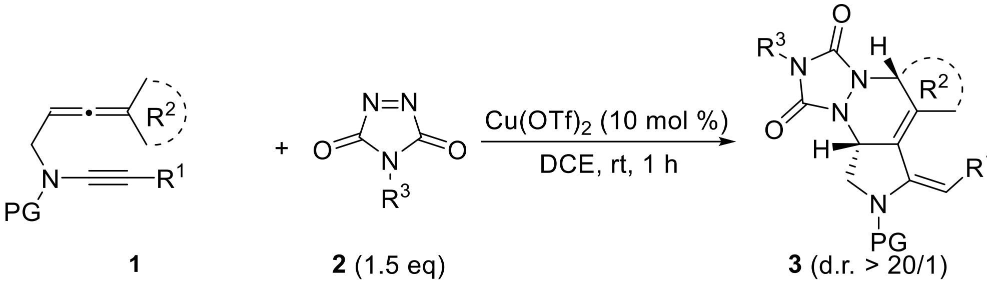

Supplementary Figure 94. Synthesis of tetracyclic pyrrolidines **3**.

General procedure for the synthesis of tetracyclic pyrrolidines 3:

PTAD (*cis*-diazenes) **2** (0.3 mmol), and $Cu(OTf)_2$ (0.02 mmol, 7.2 mg) were added in this order to the allenynes **1** (0.2 mmol) in DCE (4.0 mL) at room temperature. The reaction mixture was stirred at room temperature and the progress of the reaction was monitored by TLC. The reaction typically took 1 h. Upon completion, the mixture was then concentrated and the residue was purified by chromatography on silica gel (eluent: petroleum ether/ethyl acetate) to afford the desired products **3**.

(*E*)-1-benzylidene-6-phenyl-2-tosyl-2,3,3a,8a,9,10,11,12-octahydro-1*H*,5*H*-pyrrolo[3,4-*c*][1,2,4]triazolo[1,2-*a*]cinnoline-5,7(6*H*)-dione (3a)

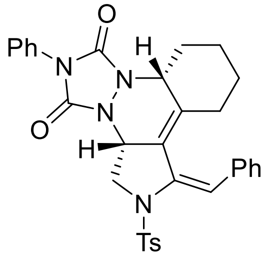

3a

The reaction was conducted with *N*-(3-cyclohexylideneallyl)-4-methyl-*N*-(phenylethynyl)benzenesulfonamide (**1a**, 0.2 mmol, 78.4 mg), 4-phenyl-3*H*-1,2,4-triazole-3,5(4*H*)-dione **2a** (0.3 mmol, 52.5 mg), and $Cu(OTf)_2$ (0.02 mmol, 7.2 mg) in DCE (4.0 mL) at room temperature. Purification by column chromatography on silica gel (petroleum ether/ethyl acetate = 5:1) yielded **3a** (83.9 mg, 74%) as a pale yellow oil.

The reaction was conducted with *N*-(3-cyclohexylideneallyl)-4-methyl-*N*-(phenylethynyl)benzenesulfonamide (**1a**, 3.0 mmol, 1176.0 mg), 4-phenyl-3*H*-1,2,4-

triazole-3,5(4*H*)-dione **2a** (4.5 mmol, 787.5 mg), and $Cu(OTf)_2$ (0.3 mmol, 108.6 mg) in DCE (30.0 mL) at room temperature. Purification by column chromatography on silica gel (petroleum ether/ethyl acetate = 5:1) yielded **3a** (1106.2 mg, 65%).

^{1}H NMR (400 MHz, $CDCl_3$) δ 7.79 (d, *J* = 8.2 Hz, 2H), 7.49 – 7.45 (m, 4H), 7.40 – 7.36 (m, 1H), 7.30 – 7.26 (m, 4H), 7.23 – 7.16 (m, 3H), 4.66 – 4.62 (m, 1H), 4.23 – 4.15 (m, 2H), 3.49 (t, *J* = 10.0 Hz, 1H), 2.39 (s, 3H), 2.30 (d, *J* = 11.8 Hz, 1H), 2.04 (d, *J* = 13.9 Hz, 1H), 1.66 (d, *J* = 13.5 Hz, 1H), 1.46 (d, *J* = 11.5 Hz, 1H), 1.35 – 1.17 (m, 3H), 1.11 – 1.04 (m, 1H), 0.67 – 0.57 (m, 1H); ^{13}C NMR (100 MHz, $CDCl_3$) δ 154.6, 150.3, 144.5, 136.5, 134.6, 134.2, 132.9, 130.8, 129.7, 129.2, 128.6, 128.3, 128.1, 127.6, 126.9, 125.2, 122.4, 115.4, 55.5, 54.6, 53.4, 31.3, 30.5, 26.6, 23.7, 21.5; IR (neat): 2925, 1776, 1716, 1635, 1596, 1502, 1417, 1352, 1239, 1165, 1043, 978, 812, 756, 666, 591, 544; HRMS (ESI) m/z: $[M + H]^+$ calcd for $C_{32}H_{31}N_4O_4S$ 567.2061, found 567.2060.

(*E*)-1-benzylidene-2-((4-methoxyphenyl)sulfonyl)-6-phenyl-2,3,3a,8a,9,10,11,12-octahydro-1*H*,5*H*-pyrrolo[3,4-*c*][1,2,4]triazolo[1,2-*a*]cinnoline-5,7(6*H*)-dione (3b)

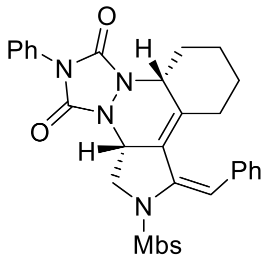

3b

The reaction was conducted with *N*-(3-cyclohexylideneallyl)-4-methoxy-*N*-(phenylethynyl)benzenesulfonamide (**1b**, 0.2 mmol, 81.6 mg), 4-phenyl-3*H*-1,2,4-triazole-3,5(4*H*)-dione **2a** (0.3 mmol, 52.5 mg), and $Cu(OTf)_2$ (0.02 mmol, 7.2 mg) in DCE (4.0 mL) at room temperature. Purification by column chromatography on silica gel (petroleum ether/ethyl acetate = 5:1) yielded **3b** (100.3 mg, 86%) as a pale yellow oil.

^{1}H NMR (400 MHz, $CDCl_3$) δ 7.85 (d, *J* = 5.9 Hz, 2H), 7.50 – 7.48 (m, 4H), 7.40 – 7.38 (m, 1H), 7.27 – 7.19 (m, 5H), 6.95 (d, *J* = 9.0 Hz, 2H), 4.65 (t, *J* = 7.6 Hz, 1H), 4.24 – 4.16 (m, 2H), 3.83 (s, 3H), 3.48 (t, *J* = 10.1 Hz, 1H), 2.31 (d, *J* = 10.1 Hz, 1H), 2.07 (d, *J* = 14.4 Hz, 1H), 1.68 (d, *J* = 13.1 Hz, 1H), 1.48 (d, *J* = 12.8 Hz, 1H), 1.36 – 1.17 (m, 3H),

1.11 – 1.05 (m, 1H), 0.75 – 0.69 (m, 1H); ^{13}C NMR (100 MHz, $CDCl_3$) δ 163.6, 154.6, 150.3, 136.5, 134.7, 133.0, 130.8, 129.7, 129.2, 128.8, 128.6, 128.3, 128.1, 126.9, 125.2, 122.5, 115.2, 114.3, 55.6, 55.5, 54.7, 53.4, 31.3, 30.5, 26.7, 23.6; IR (neat): 2929, 1774, 1719, 1596, 1498, 1420, 1350, 1263, 1161, 1092, 1027, 980, 835, 804, 670, 591, 561; HRMS (ESI) m/z: $[M + H]^+$ calcd for $C_{32}H_{31}N_4O_5S$ 583.2010, found 583.2008.

(*E*)-1-benzylidene-6-phenyl-2-(phenylsulfonyl)-2,3,3a,8a,9,10,11,12-octahydro-1*H*,5*H*-pyrrolo[3,4-*c*][1,2,4]triazolo[1,2-*a*]cinnoline-5,7(6*H*)-dione (3c)

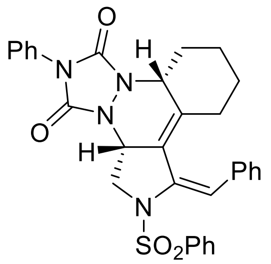

3c

The reaction was conducted with *N*-(3-cyclohexylideneallyl)-*N*-(phenylethynyl)benzenesulfonamide (**1c**, 0.2 mmol, 75.6 mg), 4-phenyl-3*H*-1,2,4-triazole-3,5(4*H*)-dione **2a** (0.3 mmol, 52.5 mg), and $Cu(OTf)_2$ (0.02 mmol, 7.2 mg) in DCE (4.0 mL) at room temperature. Purification by column chromatography on silica gel (petroleum ether/ethyl acetate = 5:1) yielded **3c** (68.6 mg, 62%) as a pale yellow oil.

^{1}H NMR (400 MHz, $CDCl_3$) δ 7.92 (d, J = 5.2 Hz, 2H), 7.67 – 7.59 (m, 1H), 7.53 – 7.48 (m, 6H), 7.40 – 7.38 (m, 1H), 7.27 – 7.18 (m, 5H), 4.66 (t, J = 7.6 Hz, 1H), 4.22 – 4.15 (m, 2H), 3.48 (t, J = 9.8 Hz, 1H), 2.30 (d, J = 10.2 Hz, 1H), 2.03 (d, J = 13.8 Hz, 1H), 1.72 – 1.63 (m, 2H), 1.45 (d, J = 12.8 Hz, 1H), 1.32 (d, J = 12.7 Hz, 1H), 1.20 – 1.05 (m, 2H), 0.64 – 0.55 (m, 1H); ^{13}C NMR (100 MHz, $CDCl_3$) δ 154.6, 150.3, 137.1, 136.4, 134.7, 133.5, 132.8, 130.8, 129.2(0), 129.1(5), 128.6, 128.3, 128.1, 127.6, 127.0, 125.2, 122.3, 115.7, 55.4, 54.5, 53.3, 31.3, 30.5, 26.7, 23.6; IR (neat): 2927, 1776, 1715, 1634, 1503, 1447, 1417, 1352, 1290, 1168, 1134, 1091, 978, 790, 690, 597, 547; HRMS (ESI) m/z: $[M + H]^+$ calcd for $C_{31}H_{29}N_4O_4S$ 553.1904, found 553.1900.

(*E*)-1-benzylidene-2-((4-bromophenyl)sulfonyl)-6-phenyl-2,3,3a,8a,9,10,11,12-octahydro-1*H*,5*H*-pyrrolo[3,4-*c*][1,2,4]triazolo[1,2-*a*]cinnoline-5,7(6*H*)-dione (3d)

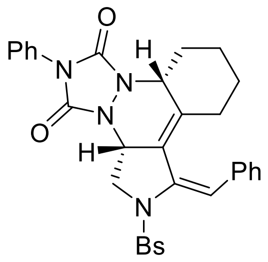

3d

The reaction was conducted with 4-bromo-*N*-(3-cyclohexylideneallyl)-*N*-(phenylethynyl)benzenesulfonamide (**1d**, 0.2 mmol, 91.2 mg), 4-phenyl-3*H*-1,2,4-triazole-3,5(4*H*)-dione **2a** (0.3 mmol, 52.5 mg), and $Cu(OTf)_2$ (0.02 mmol, 7.2 mg) in DCE (4.0 mL) at room temperature. Purification by column chromatography on silica gel (petroleum ether/ethyl acetate = 5:1) yielded **3d** (99.9 mg, 79%) as a pale yellow oil.

^{1}H NMR (400 MHz, $CDCl_3$) δ 7.77 (d, *J* = 6.7 Hz, 2H), 7.65 (d, *J* = 6.7 Hz, 2H), 7.49 – 7.48 (m, 4H), 7.40 – 7.38 (m, 1H), 7.31 – 7.27 (m, 1H), 7.23 – 7.18 (m, 4H), 4.63 (t, *J* = 9.1 Hz, 1H), 4.25 – 4.20 (m, 2H), 3.48 (t, *J* = 10.0 Hz, 1H), 2.32 (d, *J* = 10.9 Hz, 1H), 2.02 (d, *J* = 14.6 Hz, 1H), 1.70 (d, *J* = 13.6 Hz, 1H), 1.51 (d, *J* = 12.8 Hz, 1H), 1.36 – 1.10 (m, 4H), 0.67 – 0.58 (m, 1H); ^{13}C NMR (100 MHz, $CDCl_3$) δ 154.6, 150.2, 136.1, 136.0, 135.1, 132.6, 132.4, 130.7, 129.2, 128.9, 128.8, 128.6, 128.3, 128.1, 127.1, 125.2, 122.1, 115.8, 55.5, 54.5, 53.4, 31.4, 30.5, 26.7, 23.6; IR (neat): 2928, 1776, 1715, 1635, 1573, 1503, 1417, 1354, 1278, 1170, 1088, 1006, 522, 741, 697, 604, 547; HRMS (ESI) m/z: $[M + H]^+$ calcd for $C_{31}H_{28}BrN_4O_4S$ 631.1009, found 631.1007.

(*E*)-1-benzylidene-2-(methylsulfonyl)-6-phenyl-2,3,3a,8a,9,10,11,12-octahydro-1*H*,5*H*-pyrrolo[3,4-*c*][1,2,4]triazolo[1,2-*a*]cinnoline-5,7(6*H*)-dione (3e)

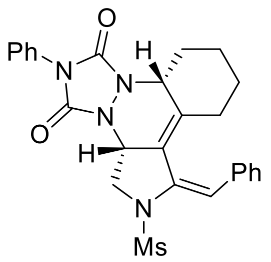

3e

The reaction was conducted with *N*-(3-cyclohexylideneallyl)-*N*-(phenylethynyl)methanesulfonamide (**1e**, 0.2 mmol, 63.0 mg), 4-phenyl-3*H*-1,2,4-triazole-3,5(4*H*)-dione **2a** (0.3 mmol, 52.5 mg), and $Cu(OTf)_2$ (0.02 mmol, 7.2 mg) in DCE (4.0 mL) at room temperature. Purification by column chromatography on silica gel (petroleum ether/ethyl acetate = 5:1) yielded **3e** (51.1 mg, 52%) as a pale yellow oil.

^{1}H NMR (400 MHz, $CDCl_3$) δ 7.53 – 7.47 (m, 4H), 7.39 (t, *J* = 6.9 Hz, 1H), 7.31 – 7.28 (m, 4H), 7.20 – 7.17 (m, 1H), 6.89 (s, 1H), 4.61 – 4.50 (m, 2H), 4.37 (d, *J* = 4.7 Hz, 1H), 3.62 (t, *J* = 9.3 Hz, 1H), 3.01 (s, 3H), 2.44 – 2.42 (m, 1H), 2.30 (d, *J* = 13.3 Hz, 1H), 1.87 – 1.80 (m, 1H), 1.66 (d, *J* = 12.3 Hz, 1H), 1.49 – 1.42 (m, 2H), 1.27 – 1.12 (m, 2H); ^{13}C NMR (100 MHz, $CDCl_3$) δ 154.7, 150.4, 136.3, 135.8, 133.0, 130.8, 129.2, 128.6, 128.4, 128.1, 126.9, 125.2, 122.3, 113.0, 55.6, 55.1, 53.3, 35.3, 31.5, 30.7, 27.1, 23.7; IR (neat): 2929, 1775, 1717, 1596, 1503, 1418, 1344, 1161, 1135, 1078, 1049, 882, 755, 692, 514; HRMS (ESI) m/z: $[M + H]^+$ calcd for $C_{26}H_{27}N_4O_4S$ 491.1748, found 491.1746.

(*E*)-1-(4-fluorobenzylidene)-6-phenyl-2-tosyl-2,3,3a,8a,9,10,11,12-octahydro-1*H*,5*H*-pyrrolo[3,4-*c*][1,2,4]triazolo[1,2-*a*]cinnoline-5,7(6*H*)-dione (3f)

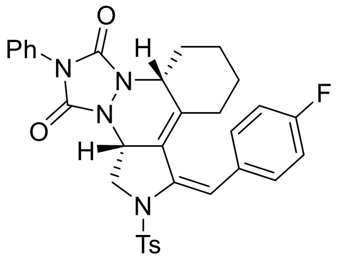

3f

The reaction was conducted with *N*-(3-cyclohexylideneallyl)-*N*-((4-fluorophenyl)ethynyl)-4-methylbenzenesulfonamide (**1f**, 0.2 mmol, 82.0 mg), 4-phenyl-

3*H*-1,2,4-triazole-3,5(4*H*)-dione **2a** (0.3 mmol, 52.5 mg), and $Cu(OTf)_2$ (0.02 mmol, 7.2 mg) in DCE (4.0 mL) at room temperature. Purification by column chromatography on silica gel (petroleum ether/ethyl acetate = 5:1) yielded **3f** (71.4 mg, 61%) as a pale yellow oil.

^{1}H NMR (400 MHz, $CDCl_3$) δ 7.78 (d, *J* = 8.1 Hz, 2H), 7.49 – 7.48 (m, 3H), 7.40 – 7.37 (m, 1H), 7.29 (d, *J* = 8.0 Hz, 1H), 7.21 – 7.18 (m, 2H), 7.13 (s, 1H), 7.00 – 6.95 (m, 2H), 4.66 – 4.61 (m, 1H), 4.23 – 4.14 (m, 2H), 3.48 (t, *J* = 10.0 Hz, 1H), 2.40 (s, 3H), 2.31 (d, *J* = 9.3 Hz, 1H), 2.09 – 2.02 (m, 1H), 1.68 (d, *J* = 13.9 Hz, 1H), 1.49 (d, *J* = 13.6 Hz, 1H), 1.37 – 1.30 (m, 1H), 1.21 – 1.08 (m, 3H), 0.69 – 0.59 (m, 1H); ^{13}C NMR (100 MHz, $CDCl_3$) δ 161.4 (d, *J* = 247.0 Hz), 154.6, 150.3, 144.5, 134.7, 134.2, 132.8 (d, *J* = 1.0 Hz), 132.5 (d, *J* = 4.0 Hz), 130.8, 129.8, 129.7, 129.2, 128.3, 127.6, 125.2, 122.4, 115.6 (d, *J* = 21.0 Hz), 114.2, 55.5, 54.6, 53.4, 31.4, 30.5, 26.6, 23.6, 21.5; IR (neat): 2927, 1774, 1719, 1637, 1507, 1420, 1351, 1290, 1227, 1166, 1092, 978, 892, 767, 663, 590, 547, 415; HRMS (ESI) m/z: $[M + H]^+$ calcd for $C_{32}H_{30}FN_4O_4S$ 585.1966, found 585.1968.

(*E*)-6-phenyl-2-tosyl-1-(4-(trifluoromethyl)benzylidene)-2,3,3a,8a,9,10,11,12-octahydro-1*H*,5*H*-pyrrolo[3,4-*c*][1,2,4]triazolo[1,2-*a*]cinnoline-5,7(6*H*)-dione (3g)

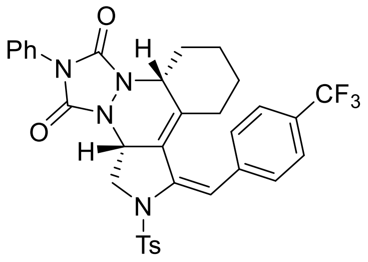

3g

The reaction was conducted with *N*-(3-cyclohexylideneallyl)-4-methyl-*N*-((4-(trifluoromethyl)phenyl)ethynyl)benzenesulfonamide (**1g**, 0.2 mmol, 92.0 mg), 4-phenyl-3*H*-1,2,4-triazole-3,5(4*H*)-dione **2a** (0.3 mmol, 52.5 mg), and $Cu(OTf)_2$ (0.02 mmol, 7.2 mg) in DCE (4.0 mL) at room temperature. Purification by column chromatography on silica gel (petroleum ether/ethyl acetate = 5:1) yielded **3g** (68.6 mg, 54%) as a pale yellow oil.

^{1}H NMR (400 MHz, $CDCl_3$) δ 7.78 (d, J = 6.9 Hz, 2H), 7.52 – 7.48 (m, 5H), 7.40 – 7.37 (m, 1H), 7.33 – 7.29 (m, 4H), 7.17 (s, 1H), 4.67 (t, J = 8.6 Hz, 1H), 4.25 – 4.19 (m, 2H), 3.52 (t, J = 9.9 Hz, 1H), 2.40 (s, 3H), 2.33 (d, J = 10.9 Hz, 1H), 2.01 (d, J = 13.1 Hz, 1H), 1.70 (d, J = 13.5 Hz, 1H), 1.51 (d, J = 12.5 Hz, 1H), 1.39 – 1.19 (m, 3H), 1.09 (t, J = 13.4 Hz, 1H), 0.75 – 0.66 (m, 1H); ^{13}C NMR (100 MHz, $CDCl_3$) δ 154.6, 150.3, 144.8, 140.3, 135.9, 134.8, 134.2, 130.8, 129.9, 129.2, 128.7, 128.4, 128.2, 127.5, 125.5 (q, J = 3.4 Hz), 125.1, 122.4, 112.9, 55.6, 54.7, 53.5, 31.5, 30.5, 26.7, 23.6, 21.6; IR (neat): 2927, 1719, 1654, 1618, 1506, 1418, 1322, 1166, 1120, 1067, 982, 869, 765, 591, 474; HRMS (ESI) m/z: $[M + H]^+$ calcd for $C_{33}H_{30}F_3N_4O_4S$ 635.1934, found 635.1933.

(*E*)-4-((5,7-dioxo-6-phenyl-2-tosyl-2,3,3a,6,7,8a,9,10,11,12-decahydro-1*H*,5*H*-pyrrolo[3,4-*c*][1,2,4]triazolo[1,2-*a*]cinnolin-1-ylidene)methyl)benzonitrile (3h)

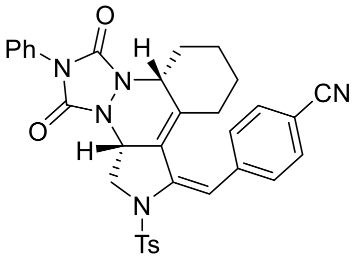

3h

The reaction was conducted with *N*-((4-cyanophenyl)ethynyl)-*N*-(3-cyclohexylideneallyl)-4-methylbenzenesulfonamide (**1h**, 0.2 mmol, 83.4 mg), 4-phenyl-3*H*-1,2,4-triazole-3,5(4*H*)-dione **2a** (0.3 mmol, 52.5 mg), and $Cu(OTf)_2$ (0.02 mmol, 7.2 mg) in DCE (4.0 mL) at room temperature. Purification by column chromatography on silica gel (petroleum ether/ethyl acetate = 5:1) yielded **3h** (108.9 mg, 92%) as a pale yellow oil.

^{1}H NMR (400 MHz, $CDCl_3$) δ 7.77 (d, J = 7.5 Hz, 2H), 7.55 (d, J = 7.7 Hz, 2H), 7.49 – 7.46 (m, 4H), 7.40 – 7.38 (m, 1H), 7.35 – 7.30 (m, 3H), 7.13 (s, 1H), 4.69 – 4.65 (m, 1H), 4.26 – 4.18 (m, 2H), 3.53 (t, J = 10.0 Hz, 1H), 2.41 (s, 3H), 2.34 (d, J = 9.7 Hz, 1H), 2.00 (d, J = 13.5 Hz, 1H), 1.72 (d, J = 13.6 Hz, 1H), 1.53 (d, J = 13.0 Hz, 1H), 1.41 – 1.13 (m, 4H), 0.79 – 0.69 (m, 1H); ^{13}C NMR (100 MHz, $CDCl_3$) δ 154.6, 150.3, 144.9, 141.5, 136.6, 135.7, 134.1, 132.3, 130.7, 129.9, 129.2, 128.4(4), 128.3(7), 127.4, 125.1, 122.5,

118.8, 112.1, 109.9, 55.6, 54.7, 53.5, 31.6, 30.5, 26.7, 23.6, 21.6; IR (neat): 2927, 1769, 1714, 1649, 1596, 1504, 1414, 1359, 1286, 1163, 1086, 965, 814, 661, 547; HRMS (ESI) m/z: $[M + H]^+$ calcd for $C_{33}H_{30}N_5O_4S$ 592.2013, found 592.2012.

methyl (*E*)-4-((5,7-dioxo-6-phenyl-2-tosyl-2,3,3a,6,7,8a,9,10,11,12-decahydro-1*H*,5*H*-pyrrolo[3,4-*c*][1,2,4]triazolo[1,2-*a*]cinnolin-1-ylidene)methyl)benzoate (3i)

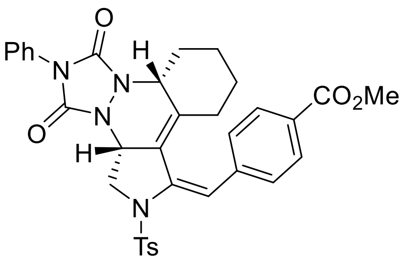

3i

The reaction was conducted with methyl 4-(((*N*-(3-cyclohexylideneallyl)-4-methylphenyl)sulfonamido)ethynyl)benzoate (**1i**, 0.2 mmol, 90.0 mg), 4-phenyl-3*H*-1,2,4-triazole-3,5(4*H*)-dione **2a** (0.3 mmol, 52.5 mg), and $Cu(OTf)_2$ (0.02 mmol, 7.2 mg) in DCE (4.0 mL) at room temperature. Purification by column chromatography on silica gel (petroleum ether/ethyl acetate = 5:1) yielded **3i** (95.0 mg, 76%) as a pale yellow oil.

^{1}H NMR (400 MHz, $CDCl_3$) δ 7.93 (d, *J* = 7.8 Hz, 2H), 7.78 (d, *J* = 7.7 Hz, 2H), 7.49 – 7.46 (m, 4H), 7.40 – 7.35 (m, 1H), 7.31 – 7.27 (m, 3H), 7.19 (s, 1H), 4.67 (t, *J* = 9.1 Hz, 1H), 4.24 – 4.17 (m, 2H), 3.92 (s, 3H), 3.52 (t, *J* = 10.0 Hz, 1H), 2.40 (s, 3H), 2.33 (d, *J* = 9.7 Hz, 1H), 2.04 – 1.99 (m, 1H), 1.69 (d, *J* = 13.1 Hz, 1H), 1.48 (d, *J* = 11.3 Hz, 1H), 1.37 – 1.21 (m, 3H), 1.10 (t, *J* = 13.3 Hz, 1H), 0.75 – 0.65 (m, 1H); ^{13}C NMR (100 MHz, $CDCl_3$) δ 166.7, 154.6, 150.3, 144.7, 141.5, 135.9, 134.8, 134.2, 130.8, 129.9, 129.8, 129.2, 128.3, 128.2, 127.9, 127.5, 125.2, 122.5, 113.5, 55.6, 54.7, 53.5, 52.1, 31.5, 30.5, 26.6, 23.6, 21.6; IR (neat): 2928, 1775, 1719, 1603, 1503, 1413, 1352, 1279, 1166, 1113, 1043, 896, 767, 659, 591, 546; HRMS (ESI) m/z: $[M + H]^+$ calcd for $C_{34}H_{33}N_4O_6S$ 625.2115, found 625.2114.

(*E*)-1-(4-(*tert*-butyl)benzylidene)-6-phenyl-2-tosyl-2,3,3a,8a,9,10,11,12-octahydro-1*H*,5*H*-pyrrolo[3,4-*c*][1,2,4]triazolo[1,2-*a*]cinnoline-5,7(6*H*)-dione (3j)

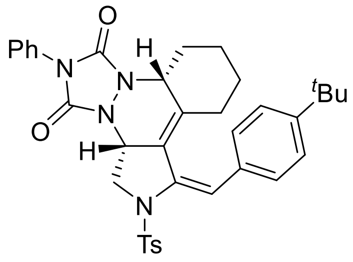

3j

The reaction was conducted with *N*-((4-(*tert*-butyl)phenyl)ethynyl)-*N*-(3-cyclohexylideneallyl)-4-methylbenzenesulfonamide (**1j**, 0.2 mmol, 89.6 mg), 4-phenyl-3*H*-1,2,4-triazole-3,5(4*H*)-dione **2a** (0.3 mmol, 52.5 mg), and $Cu(OTf)_2$ (0.02 mmol, 7.2 mg) in DCE (4.0 mL) at room temperature. Purification by column chromatography on silica gel (petroleum ether/ethyl acetate = 5:1) yielded **3j** (81.0 mg, 65%) as a pale yellow oil.

^{1}H NMR (400 MHz, $CDCl_3$) δ 7.79 (d, *J* = 7.8 Hz, 2H), 7.49 – 7.46 (m, 3H), 7.40 – 7.35 (m, 1H), 7.29 – 7.26 (m, 4H), 7.22 – 7.13 (m, 3H), 4.62 (t, *J* = 9.0 Hz, 1H), 4.26 – 4.15 (m, 2H), 3.47 (t, *J* = 9.9 Hz, 1H), 2.39 (s, 3H), 2.31 (d, *J* = 10.0 Hz, 1H), 2.10 – 2.04 (m, 1H), 1.66 (d, *J* = 13.3 Hz, 1H), 1.49 – 1.37 (m, 2H), 1.31 (s, 9H), 1.19 – 1.11 (m, 3H), 0.63 – 0.54 (m, 1H); ^{13}C NMR (100 MHz, $CDCl_3$) δ 154.6, 150.3, 150.0, 144.4, 134.4, 134.2, 133.3, 132.1, 130.9, 129.7, 129.2, 128.3, 127.9, 127.6, 125.4, 125.2, 122.5, 115.7, 55.4, 54.6, 53.3, 34.6, 31.4, 31.2, 30.6, 26.6, 23.7, 21.5; IR (neat): 2927, 1771, 1719, 1654, 1502, 1410, 1349, 1276, 1163, 1090, 1041, 858, 750, 661, 591, 477; HRMS (ESI) m/z: $[M + Na]^+$ calcd for $C_{36}H_{38}N_4NaO_4S$ 645.2506, found 645.2500.

(*E*)-1-(4-methoxybenzylidene)-6-phenyl-2-tosyl-2,3,3a,8a,9,10,11,12-octahydro-1*H*,5*H*-pyrrolo[3,4-*c*][1,2,4]triazolo[1,2-*a*]cinnoline-5,7(6*H*)-dione (3k)

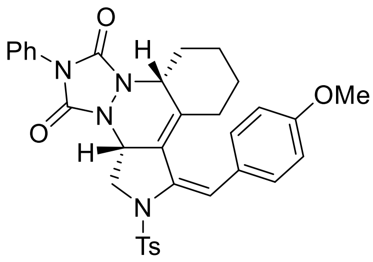

3k

The reaction was conducted with *N*-(3-cyclohexylideneallyl)-*N*-((4-methoxyphenyl)ethynyl)-4-methylbenzenesulfonamide (**1k**, 0.2 mmol, 84.4 mg), 4-phenyl-3*H*-1,2,4-triazole-3,5(4*H*)-dione **2a** (0.3 mmol, 52.5 mg), and $Cu(OTf)_2$ (0.02 mmol, 7.2 mg) in DCE (4.0 mL) at room temperature. Purification by column chromatography on silica gel (petroleum ether/ethyl acetate = 5:1) yielded **3k** (77.6 mg, 65%) as a pale yellow oil.

^{1}H NMR (400 MHz, $CDCl_3$) δ 7.78 (d, *J* = 8.2 Hz, 2H), 7.50 – 7.47 (m, 3H), 7.40 – 7.36 (m, 1H), 7.28 (d, *J* = 8.3 Hz, 2H), 7.18 – 7.14 (m, 3H), 6.81 (d, *J* = 8.6 Hz, 2H), 4.64 – 4.60 (m, 1H), 4.24 – 4.13 (m, 2H), 3.81 (s, 3H), 3.46 (t, *J* = 10.0 Hz, 1H), 2.39 (s, 3H), 2.30 (d, *J* = 9.1 Hz, 1H), 2.08 (d, *J* = 14.1 Hz, 1H), 1.67 (d, *J* = 13.6 Hz, 1H), 1.47 (d, *J* = 13.3 Hz, 1H), 1.34 (d, *J* = 13.4 Hz, 1H), 1.26 – 1.15 (m, 3H), 0.65 – 0.55 (m, 1H); ^{13}C NMR (100 MHz, $CDCl_3$) δ 158.4, 154.6, 150.3, 144.4, 134.2, 134.1, 131.4, 130.9, 129.7, 129.4, 129.2, 128.8, 128.3, 127.6, 125.2, 122.6, 115.7, 114.0, 55.4, 55.2, 54.6, 53.3, 31.4, 30.5, 26.6, 23.7, 21.5; IR (neat): 2927, 1773, 1718, 1637, 1508, 1414, 1349, 1249, 1165, 1133, 1034, 976, 854, 750, 590, 469, 412, 405; HRMS (ESI) m/z: $[M + H]^+$ calcd for $C_{33}H_{33}N_4O_5S$ 597.2166, found 597.2164.

(*E*)-1-(3-bromobenzylidene)-6-phenyl-2-tosyl-2,3,3a,8a,9,10,11,12-octahydro-1*H*,5*H*-pyrrolo[3,4-*c*][1,2,4]triazolo[1,2-*a*]cinnoline-5,7(6*H*)-dione (3l)

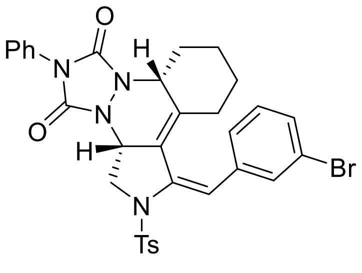

3l

The reaction was conducted with *N*-((3-bromophenyl)ethynyl)-*N*-(3-cyclohexylideneallyl)-4-methylbenzenesulfonamide (**1l**, 0.2 mmol, 94.0 mg), 4-phenyl-3*H*-1,2,4-triazole-3,5(4*H*)-dione **2a** (0.3 mmol, 52.5 mg), and $Cu(OTf)_2$ (0.02 mmol, 7.2 mg) in DCE (4.0 mL) at room temperature. Purification by column chromatography on

silica gel (petroleum ether/ethyl acetate = 5:1) yielded **3l** (81.4 mg, 63%) as a yellow solid (mp 161-163 °C).

^{1}H NMR (400 MHz, $CDCl_3$) δ 7.78 (d, *J* = 5.3 Hz, 2H), 7.50 – 7.49 (m, 3H), 7.40 – 7.36 (m, 2H), 7.31 – 7.30 (m, 3H), 7.15 – 7.09 (m, 3H), 4.65 (t, *J* = 9.1 Hz, 1H), 4.27 – 4.20 (m, 2H), 3.50 (t, *J* = 10.0 Hz, 1H), 2.41 (s, 3H), 2.34 (d, *J* = 10.1 Hz, 1H), 2.03 (d, *J* = 13.2 Hz, 1H), 1.70 (d, *J* = 12.5 Hz, 1H), 1.53 (d, *J* = 12.3 Hz, 1H), 1.40 – 1.34 (m, 1H), 1.24 – 1.14 (m, 3H), 0.73 – 0.64 (m, 1H); ^{13}C NMR (100 MHz, $CDCl_3$) δ 154.6, 150.3, 144.7, 138.6, 135.5, 134.2, 134.0, 130.8, 130.6, 130.1, 129.8, 129.7, 129.2, 128.4, 127.5, 126.8, 125.2, 122.6, 122.3, 113.2, 55.5, 54.6, 53.4, 31.6, 30.5, 26.7, 23.6, 21.6; IR (neat): 2927, 1776, 1719, 1637, 1502, 1416, 1352, 1292, 1166, 1133, 1039, 976, 843, 768, 662, 592, 560; HRMS (ESI) m/z: $[M + H]^+$ calcd for $C_{32}H_{30}BrN_4O_4S$ 645.1166, found 645.1163.

(*E*)-1-(3-methylbenzylidene)-6-phenyl-2-tosyl-2,3,3a,8a,9,10,11,12-octahydro-1*H*,5*H*-pyrrolo[3,4-*c*][1,2,4]triazolo[1,2-*a*]cinnoline-5,7(6*H*)-dione (3m)

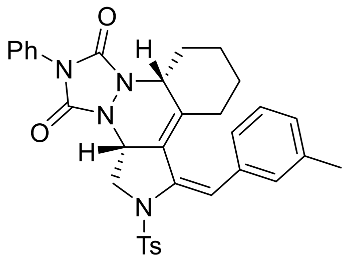

3m

The reaction was conducted with *N*-(3-cyclohexylideneallyl)-4-methyl-*N*-(*m*-tolylethynyl)benzenesulfonamide (**1m**, 0.2 mmol, 81.2 mg), 4-phenyl-3*H*-1,2,4-triazole-3,5(4*H*)-dione **2a** (0.3 mmol, 52.5 mg), and $Cu(OTf)_2$ (0.02 mmol, 7.2 mg) in DCE (4.0 mL) at room temperature. Purification by column chromatography on silica gel (petroleum ether/ethyl acetate = 5:1) yielded **3m** (88.3 mg, 76%) as a pale yellow oil.

^{1}H NMR (400 MHz, $CDCl_3$) δ 7.78 (d, *J* = 8.3 Hz, 2H), 7.50 – 7.45 (m, 4H), 7.39 – 7.36 (m, 1H), 7.28 (d, *J* = 8.2 Hz, 2H), 7.17 – 7.13 (m, 2H), 7.04 (s, 1H), 7.01 – 6.98 (m, 3H), 4.65 – 4.61 (m, 1H), 4.23 – 4.16 (m, 2H), 3.47 (t, *J* = 10.0 Hz, 1H), 2.39 (s, 3H), 2.31 (s, 3H), 2.05 – 2.02 (m, 1H), 1.72 – 1.65 (m, 2H), 1.47 (d, *J* = 13.7 Hz, 1H), 1.36 – 1.07 (m,

4H), 0.66 – 0.56 (m, 1H); ^{13}C NMR (100 MHz, $CDCl_3$) δ 154.5, 150.3, 144.4, 138.1, 136.2, 134.5, 134.2, 132.6, 130.8, 129.7, 129.2, 128.9, 128.4, 128.3, 127.7, 127.6, 125.2, 125.0, 122.4, 115.7, 55.4, 54.6, 53.3, 31.4, 30.5, 26.5, 23.6, 21.5, 21.4; IR (neat): 2929, 1774, 1717, 1637, 1500, 1419, 1350, 1165, 1133, 1091, 980, 845, 768, 689, 663, 593, 503; HRMS (ESI) m/z: $[M + H]^+$ calcd for $C_{33}H_{33}N_4O_4S$ 581.2217, found 581.2216.

(*E*)-1-(3,4-dichlorobenzylidene)-6-phenyl-2-tosyl-2,3,3a,8a,9,10,11,12-octahydro-1*H*,5*H*-pyrrolo[3,4-*c*][1,2,4]triazolo[1,2-*a*]cinnoline-5,7(6*H*)-dione (3n)

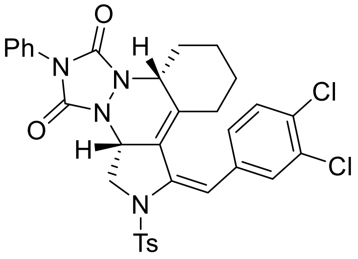

3n

The reaction was conducted with *N*-(3-cyclohexylideneallyl)-*N*-((3,4-dichlorophenyl)ethynyl)-4-methylbenzenesulfonamide (**1n**, 0.2 mmol, 92.0 mg), 4-phenyl-3*H*-1,2,4-triazole-3,5(4*H*)-dione **2a** (0.3 mmol, 52.5 mg), and $Cu(OTf)_2$ (0.02 mmol, 7.2 mg) in DCE (4.0 mL) at room temperature. Purification by column chromatography on silica gel (petroleum ether/ethyl acetate = 5:1) yielded **3n** (95.4 mg, 75%) as a pale yellow oil.

^{1}H NMR (400 MHz, $CDCl_3$) δ 7.76 (d, *J* = 6.9 Hz, 2H), 7.49 – 7.45 (m, 3H), 7.41 – 7.37 (m, 1H), 7.34 – 7.29 (m, 4H), 7.10 – 7.04 (m, 2H), 4.67 – 4.63 (m, 1H), 4.27 – 4.17 (m, 2H), 3.50 (t, *J* = 10.0 Hz, 1H), 2.40 (s, 3H), 2.34 (d, *J* = 10.5 Hz, 1H), 2.03 (d, *J* = 12.4 Hz, 1H), 1.71 (d, *J* = 12.9 Hz, 1H), 1.55 (d, *J* = 13.0 Hz, 1H), 1.45 – 1.38 (m, 1H), 1.25 – 1.19 (m, 3H), 0.75 – 0.66 (m, 1H); ^{13}C NMR (100 MHz, $CDCl_3$) δ 154.6, 150.3, 144.8, 136.5, 135.9, 134.4, 134.1, 132.6, 130.7, 130.5, 130.4, 129.8, 129.5, 129.2, 128.4, 127.5, 127.3, 125.2, 122.3, 112.0, 55.5, 54.6, 53.4, 31.7, 30.5, 26.7, 23.6, 21.6; IR (neat): 2935, 1776, 1718, 1637, 1502, 1416, 1353, 1290, 1220, 1165, 1090, 978, 813, 661, 595, 543, 405; HRMS (ESI) m/z: $[M + H]^+$ calcd for $C_{32}H_{29}Cl_2N_4O_4S$ 635.1281, found 635.1282.

(*E*)-1-(3,5-dimethylbenzylidene)-6-phenyl-2-tosyl-2,3,3a,8a,9,10,11,12-octahydro-1*H*,5*H*-pyrrolo[3,4-*c*][1,2,4]triazolo[1,2-*a*]cinnoline-5,7(6*H*)-dione (3o)

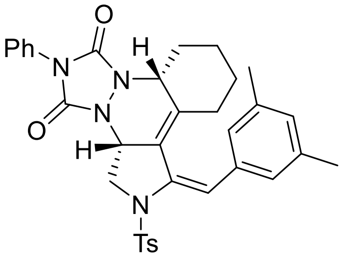

3o

The reaction was conducted with *N*-(3-cyclohexylideneallyl)-*N*-((3,5-dimethylphenyl)ethynyl)-4-methylbenzenesulfonamide (**1o**, 0.2 mmol, 84.0 mg), 4-phenyl-3*H*-1,2,4-triazole-3,5(4*H*)-dione **2a** (0.3 mmol, 52.5 mg), and $Cu(OTf)_2$ (0.02 mmol, 7.2 mg) in DCE (4.0 mL) at room temperature. Purification by column chromatography on silica gel (petroleum ether/ethyl acetate = 5:1) yielded **3o** (77.4 mg, 65%) as a pale yellow oil.

^{1}H NMR (400 MHz, $CDCl_3$) δ 7.78 (d, J = 8.1 Hz, 2H), 7.49 – 7.45 (m, 3H), 7.40 – 7.37 (m, 1H), 7.28 (d, J = 8.1 Hz, 2H), 7.13 (s, 1H), 6.90 – 6.82 (m, 3H), 4.65 – 4.60 (m, 1H), 4.22 – 4.18 (m, 2H), 3.47 (t, J = 10.0 Hz, 1H), 2.39 (s, 3H), 2.32 (d, J = 12.3 Hz, 1H), 2.26 (s, 6H), 2.03 (d, J = 13.9 Hz, 1H), 1.67 (d, J = 13.6 Hz, 1H), 1.48 (d, J = 12.8 Hz, 1H), 1.37 – 1.09 (m, 4H), 0.66 – 0.56 (m, 1H); ^{13}C NMR (100 MHz, $CDCl_3$) δ 154.5, 150.4, 144.3, 137.9, 136.0, 134.3, 134.2, 132.3, 130.8, 129.7, 129.2, 128.7, 128.3, 127.6, 125.9, 125.2, 122.5, 116.0, 55.5, 54.6, 53.2, 31.6, 30.6, 26.6, 23.7, 21.5, 21.3; IR (neat): 2927, 1775, 1719, 1597, 1503, 1414, 1352, 1288, 1166, 1133, 1090, 1041, 908, 765, 657, 594, 541; HRMS (ESI) m/z: $[M + H]^+$ calcd for $C_{34}H_{35}N_4O_4S$ 595.2374, found 595.2371.

(*E*)-1-(benzo[*d*][1,3]dioxol-5-ylmethylene)-6-phenyl-2-tosyl-2,3,3a,8a,9,10,11,12-octahydro-1*H*,5*H*-pyrrolo[3,4-*c*][1,2,4]triazolo[1,2-*a*]cinnoline-5,7(6*H*)-dione (3p)

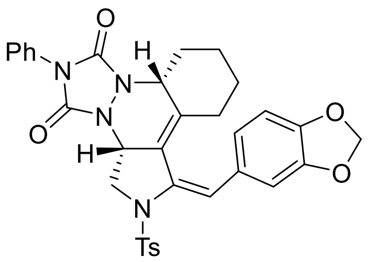

3p

The reaction was conducted with *N*-(benzo[*d*][1,3]dioxol-5-ylethynyl)-*N*-(3-cyclohexylideneallyl)-4-methylbenzenesulfonamide (**1p**, 0.2 mmol, 87.2 mg), 4-phenyl-3*H*-1,2,4-triazole-3,5(4*H*)-dione **2a** (0.3 mmol, 52.5 mg), and $Cu(OTf)_2$ (0.02 mmol, 7.2 mg) in DCE (4.0 mL) at room temperature. Purification by column chromatography on silica gel (petroleum ether/ethyl acetate = 5:1) yielded **3p** (50.1 mg, 41%) as a pale yellow oil.

^{1}H NMR (400 MHz, $CDCl_3$) δ 7.77 (d, *J* = 7.7 Hz, 2H), 7.49 – 7.45 (m, 3H), 7.40 – 7.38 (m, 1H), 7.29 (d, *J* = 7.9 Hz, 2H), 7.11 (s, 1H), 6.78 – 6.71 (m, 3H), 5.96 (d, *J* = 4.0 Hz, 2H), 4.65 – 4.60 (m, 1H), 4.24 (d, *J* = 10.8 Hz, 1H), 4.13 (t, *J* = 8.3 Hz, 1H), 3.46 (t, *J* = 10.0 Hz, 1H), 2.40 (s, 3H), 2.31 (d, *J* = 9.1 Hz, 1H), 2.11 (d, *J* = 13.6 Hz, 1H), 1.68 (d, *J* = 13.5 Hz, 1H), 1.52 (d, *J* = 12.8 Hz, 1H), 1.37 (d, *J* = 13.5 Hz, 1H), 1.26 – 1.14 (m, 3H), 0.68 – 0.58 (m, 1H); ^{13}C NMR (100 MHz, $CDCl_3$) δ 154.6, 150.3, 147.8, 146.4, 144.4, 134.5, 134.2, 131.8, 130.8, 130.3, 129.7, 129.2, 128.3, 127.6, 125.2, 122.5(3), 122.4(6), 115.6, 108.5, 107.7, 101.1, 55.5, 54.6, 53.3, 31.4, 30.6, 26.6, 23.7, 21.5; IR (neat): 2925, 1775, 1719, 1596, 1503, 1417, 1351, 1259, 1240, 1165, 1039, 929, 810, 735, 663, 588, 545; HRMS (ESI) m/z: $[M + Na]^+$ calcd for $C_{33}H_{30}N_4NaO_6S$ 633.1778, found 633.1776.

(*E*)-6-phenyl-1-(thiophen-3-ylmethylene)-2-tosyl-2,3,3a,8a,9,10,11,12-octahydro-1*H*,5*H*-pyrrolo[3,4-*c*][1,2,4]triazolo[1,2-*a*]cinnoline-5,7(6*H*)-dione (3q)

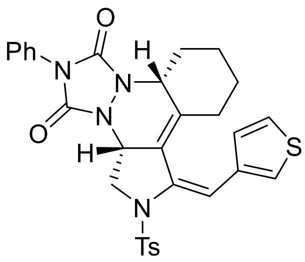

3q

The reaction was conducted with *N*-(3-cyclohexylideneallyl)-4-methyl-*N*-(thiophen-3-ylethynyl)benzenesulfonamide (**1q**, 0.2 mmol, 79.6 mg), 4-phenyl-3*H*-1,2,4-triazole-3,5(4*H*)-dione **2a** (0.3 mmol, 52.5 mg), and $Cu(OTf)_2$ (0.02 mmol, 7.2 mg) in DCE (4.0 mL) at room temperature. Purification by column chromatography on silica gel (petroleum ether/ethyl acetate = 5:1) yielded **3q** (49.3 mg, 43%) as a pale yellow oil.

^{1}H NMR (400 MHz, $CDCl_3$) δ 7.78 (d, *J* = 7.8 Hz, 2H), 7.49 – 7.45 (m, 3H), 7.40 – 7.37 (m, 1H), 7.29 (d, *J* = 7.9 Hz, 2H), 7.24 – 7.22 (m, 1H), 7.20 – 7.12 (m, 2H), 6.99 (d, *J* = 5.0 Hz, 1H), 4.62 – 4.57 (m, 1H), 4.28 (d, *J* = 10.2 Hz, 1H), 4.19 (t, *J* = 8.4 Hz, 1H), 3.44 (t, *J* = 9.9 Hz, 1H), 2.39 (s, 3H), 2.32 (d, *J* = 11.0 Hz, 1H), 2.14 (d, *J* = 13.6 Hz, 1H), 1.68 (d, *J* = 13.5 Hz, 1H), 1.51 (d, *J* = 12.8 Hz, 1H), 1.39 – 1.34 (m, 3H), 1.20 – 1.14 (m, 1H), 0.63 – 0.53 (m, 1H); ^{13}C NMR (100 MHz, $CDCl_3$) δ 154.6, 150.4, 144.4, 137.0, 135.2, 134.1, 132.4, 130.8, 129.7, 129.2, 128.3, 127.6, 126.9, 125.5, 125.2, 123.6, 122.9, 110.3, 55.5, 54.5, 53.2, 31.5, 30.6, 26.7, 23.7, 21.5; IR (neat): 2927, 1776, 1716, 1594, 1502, 1417, 1351, 1288, 1165, 1088, 1039, 978, 767, 659, 588, 541; HRMS (ESI) m/z: $[M + H]^+$ calcd for $C_{30}H_{29}N_4O_4S_2$ 573.1625, found 573.1629.

(*E*)-1-(cyclopropylmethylene)-6-phenyl-2-tosyl-2,3,3a,8a,9,10,11,12-octahydro-1*H*,5*H*-pyrrolo[3,4-*c*][1,2,4]triazolo[1,2-*a*]cinnoline-5,7(6*H*)-dione (3r)

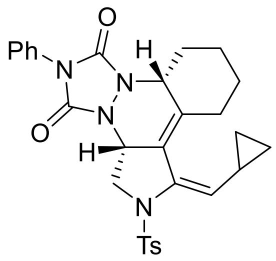

3r

The reaction was conducted with *N*-(3-cyclohexylideneallyl)-*N*-(cyclopropylethynyl)-4-methylbenzenesulfonamide (**1r**, 0.2 mmol, 71.0 mg), 4-phenyl-3*H*-1,2,4-triazole-3,5(4*H*)-dione **2a** (0.3 mmol, 52.5 mg), and $Cu(OTf)_2$ (0.02 mmol, 7.2 mg) in DCE (4.0 mL) at room temperature. Purification by column chromatography on silica gel (petroleum ether/ethyl acetate = 5:1) yielded **3r** (25.5 mg, 24%) as a pale yellow oil.

^{1}H NMR (400 MHz, $CDCl_3$) δ 7.76 (d, *J* = 8.0 Hz, 2H), 7.50 – 7.44 (m, 4H), 7.36 (t, *J* = 6.8 Hz, 1H), 7.30 (d, *J* = 8.0 Hz, 2H), 4.85 (d, *J* = 10.4 Hz, 1H), 4.36 – 4.31 (m, 1H), 4.25 (d, *J* = 10.8 Hz, 1H), 4.04 (t, *J* = 8.3 Hz, 1H), 3.73 – 3.68 (m, 1H), 2.71 (d, *J* = 13.8 Hz, 1H), 2.41 (s, 3H), 2.29 (d, *J* = 10.9 Hz, 1H), 1.82 (t, *J* = 13.3 Hz, 1H), 1.70 (d, *J* = 13.6 Hz, 1H), 1.62 (d, *J* = 13.5 Hz, 1H), 1.45 (d, *J* = 13.6 Hz, 1H), 1.15 – 1.08 (m, 1H), 0.95 – 0.84 (m, 3H), 0.69 – 0.66 (m, 1H), 0.59 – 0.54 (m, 1H), 0.48 – 0.44 (m, 1H); ^{13}C NMR (100 MHz, $CDCl_3$) δ 155.0, 149.7, 144.0, 133.9, 131.7, 130.9, 130.0, 129.6, 129.1, 128.4, 128.2, 127.8, 125.2, 124.4, 54.7, 54.4, 52.3, 30.5, 29.0, 26.0, 23.9, 21.5, 12.2, 9.0, 7.9; IR (neat): 2931, 1773, 1718, 1654, 1500, 1420, 1355, 1286, 1167, 1090, 978, 813, 765, 576, 502, 411; HRMS (ESI) m/z: $[M + H]^+$ calcd for $C_{29}H_{31}N_4O_4S$ 531.2061, found 531.2060.

(*E*)-1-benzylidene-10-ethyl-6-phenyl-2-tosyl-2,3,3a,8a,9,10,11,12-octahydro-1*H*,5*H*-pyrrolo[3,4-*c*][1,2,4]triazolo[1,2-*a*]cinnoline-5,7(6*H*)-dione (3s)

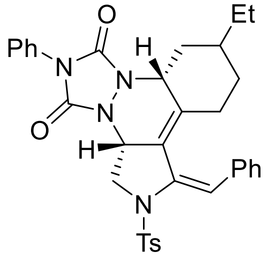

3s

The reaction was conducted with *N*-(3-(4-ethylcyclohexylidene)allyl)-4-methyl-*N*-(phenylethynyl)benzenesulfonamide (**1s**, 0.2 mmol, 84.0 mg), 4-phenyl-3*H*-1,2,4-triazole-3,5(4*H*)-dione **2a** (0.3 mmol, 52.5 mg), and $Cu(OTf)_2$ (0.02 mmol, 7.2 mg) in DCE (4.0 mL) at room temperature. Purification by column chromatography on silica gel (petroleum ether/ethyl acetate = 5:1) yielded **3s** (83.3 mg, 70%) as a pale yellow oil.

^{1}H NMR (400 MHz, $CDCl_3$) δ 7.79 (d, *J* = 8.2 Hz, 2H), 7.49 – 7.45 (m, 3H), 7.39 – 7.37 (m, 1H), 7.30 – 7.26 (m, 4H), 7.24 – 7.18 (m, 4H), 4.67 – 4.62 (m, 1H), 4.38 (d, *J* = 9.6 Hz, 1H), 4.19 – 4.15 (m, 1H), 3.49 (t, *J* = 10.1 Hz, 1H), 2.39 (s, 3H), 2.21 (d, *J* = 12.0 Hz, 1H), 1.87 (d, *J* = 14.1 Hz, 1H), 1.40 – 1.26 (m, 6H), 0.93 – 0.76 (m, 5H); ^{13}C NMR (100 MHz, $CDCl_3$) δ 154.6, 150.4, 144.5, 136.5, 135.3, 134.3, 132.9, 130.8, 129.7, 129.2,

128.6, 128.3, 128.1, 127.6, 126.9, 125.1, 122.3, 115.4, 54.5, 53.4, 51.6, 34.2, 33.6, 30.0, 27.0, 23.9, 21.5, 12.1; IR (neat): 2927, 1773, 1719, 1654, 1500, 1420, 1349, 1271, 1166, 1088, 1031, 870, 750, 590, 495, 459; HRMS (ESI) m/z: $[M + H]^+$ calcd for $C_{34}H_{35}N_4O_4S$ 595.2374, found 595.2369.

(*E*)-1-benzylidene-10-(*tert*-butyl)-6-phenyl-2-tosyl-2,3,3a,8a,9,10,11,12-octahydro-1*H*,5*H*-pyrrolo[3,4-*c*][1,2,4]triazolo[1,2-*a*]cinnoline-5,7(6*H*)-dione (3t)

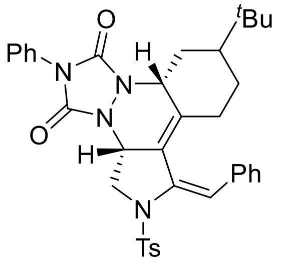

3t

The reaction was conducted with *N*-(3-(4-(*tert*-butyl)cyclohexylidene)allyl)-4-methyl-*N*-(phenylethynyl)benzenesulfonamide (**1t**, 0.2 mmol, 89.6 mg), 4-phenyl-3*H*-1,2,4-triazole-3,5(4*H*)-dione **2a** (0.3 mmol, 52.5 mg), and $Cu(OTf)_2$ (0.02 mmol, 7.2 mg) in DCE (4.0 mL) at room temperature. Purification by column chromatography on silica gel (petroleum ether/ethyl acetate = 5:1) yielded **3t** (82.2 mg, 66%) as a pale yellow oil.

^{1}H NMR (400 MHz, $CDCl_3$) δ 7.64 (d, *J* = 8.1 Hz, 2H), 7.50 – 7.37 (m, 6H), 7.32 – 7.24 (m, 6H), 4.56 – 4.51 (m, 1H), 4.33 (d, *J* = 15.6 Hz, 1H), 4.15 – 4.09 (m, 1H), 3.95 – 3.89 (m, 1H), 3.11 (d, *J* = 18.5 Hz, 1H), 2.59 (d, *J* = 11.9 Hz, 1H), 2.50 (d, *J* = 15.8 Hz, 2H), 2.43 (s, 3H), 1.91 (d, *J* = 10.9 Hz, 1H), 1.36 – 1.32 (m, 2H), 1.16 – 1.12 (m, 1H), 0.90 (s, 9H); ^{13}C NMR (100 MHz, $CDCl_3$) δ 151.6, 145.6, 144.2, 138.0, 137.2, 134.5, 130.9, 130.5, 129.8, 129.2, 128.6, 128.5, 128.4, 127.4, 126.6, 125.8, 120.2, 107.3, 57.3, 55.0, 43.6, 33.4, 32.4, 28.3, 27.6, 26.9, 23.3, 21.7; IR (neat): 2960, 1773, 1719, 1599, 1503, 1413, 1353, 1290, 1166, 1090, 971, 814, 759, 663, 589, 549; HRMS (ESI) m/z: $[M + Na]^+$ calcd for $C_{36}H_{38}N_4NaO_4S$ 645.2506, found 645.2502.

***tert*-butyl (*E*)-(1-benzylidene-5,7-dioxo-6-phenyl-2-tosyl-2,3,3a,6,7,8a,9,10,11,12-decahydro-1*H*,5*H*-pyrrolo[3,4-*c*][1,2,4]triazolo[1,2-*a*]cinnolin-10-yl)carbamate (3u)**

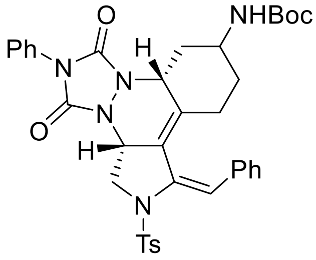

3u

The reaction was conducted with *tert*-butyl (4-(3-((4-methyl-*N*-(phenylethynyl)phenyl)sulfonamido)prop-1-en-1-ylidene)cyclohexyl)carbamate (**1u**, 0.2 mmol, 101.4 mg), 4-phenyl-3*H*-1,2,4-triazole-3,5(4*H*)-dione **2a** (0.3 mmol, 52.5 mg), and $Cu(OTf)_2$ (0.02 mmol, 7.2 mg) in DCE (4.0 mL) at room temperature. Purification by column chromatography on silica gel (petroleum ether/ethyl acetate = 5:1) yielded **3u** (80.5 mg, 59%) as a pale yellow oil.

^{1}H NMR (400 MHz, $CDCl_3$) δ 7.69 – 7.65 (m, 4H), 7.48 – 7.45 (m, 4H), 7.40 – 7.38 (m, 1H), 7.36 – 7.32 (m, 2H), 7.28 – 7.24 (m, 3H), 6.45 (s, 1H), 4.90 (d, *J* = 6.1 Hz, 1H), 4.59 (d, *J* = 9.4 Hz, 1H), 4.45 – 4.40 (m, 1H), 4.05 (t, *J* = 8.1 Hz, 1H), 3.89 (s, 1H), 3.78 – 3.73 (m, 1H), 3.03 (d, *J* = 14.7 Hz, 1H), 2.40 (s, 3H), 2.32 (t, *J* = 13.4 Hz, 1H), 1.95 (d, *J* = 11.5 Hz, 1H), 1.46 (s, 9H), 1.39 – 1.33 (m, 3H); ^{13}C NMR (100 MHz, $CDCl_3$) δ 155.2, 155.0, 149.8, 144.5, 135.4, 134.2, 131.3, 130.8, 129.7, 129.3, 129.2, 128.3(2), 128.2(8), 127.9(8), 127.9(6), 126.4, 125.1, 123.5, 80.0, 55.0, 52.1, 50.5, 45.7, 34.5, 28.4, 24.6, 21.6; IR (neat): 2929, 1773, 1717, 1654, 1500, 1420, 1363, 1251, 1166, 1088, 978, 855, 769, 690, 581, 463, 407; HRMS (ESI) m/z: $[M + H]^+$ calcd for $C_{37}H_{40}N_5O_6S$ 682.2694, found 682.2700.

(*E*)-1-benzylidene-6-phenyl-2-tosyl-1,2,3,3a,8a,9,10,11-octahydro-5*H*-cyclopenta[c]pyrrolo[3,4-*e*][1,2,4]triazolo[1,2-*a*]pyridazine-5,7(6*H*)-dione (3v)

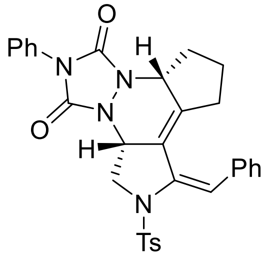

3v

The reaction was conducted with *N*-(3-cyclopentylideneallyl)-4-methyl-*N*-(phenylethynyl)benzenesulfonamide (**1v**, 0.2 mmol, 75.6 mg), 4-phenyl-3*H*-1,2,4-triazole-3,5(4*H*)-dione **2a** (0.3 mmol, 52.5 mg), and $Cu(OTf)_2$ (0.02 mmol, 7.2 mg) in DCE (4.0 mL) at room temperature. Purification by column chromatography on silica gel (petroleum ether/ethyl acetate = 5:1) yielded **3v** (88.5 mg, 80%) as a pale yellow oil.

^{1}H NMR (400 MHz, $CDCl_3$) δ 7.55 (d, *J* = 8.2 Hz, 2H), 7.40 – 7.34 (m, 4H), 7.24 – 7.17 (m, 8H), 4.54 – 4.49 (m, 1H), 4.24 – 4.18 (m, 1H), 4.12 – 4.07 (m, 1H), 3.95 – 3.82 (m, 2H), 3.09 – 3.03 (m, 1H), 2.96 – 2.88 (m, 1H), 2.79 – 2.71 (m, 1H), 2.63 – 2.57 (m, 1H), 2.36 (s, 3H), 2.07 – 1.94 (m, 1H), 0.82 – 0.76 (m, 1H); ^{13}C NMR (100 MHz, $CDCl_3$) δ 151.5, 145.1, 144.2, 137.8, 136.1, 134.6, 132.1, 130.6, 129.9, 129.2, 128.6, 128.4(9), 128.4(5), 127.4, 126.7, 125.5, 117.8, 111.1, 57.4, 55.1, 32.9, 30.5, 29.9, 21.8, 21.6; IR (neat): 2923, 2852, 1773, 1719, 1648, 1597, 1502, 1416, 1288, 1163, 1089, 1030; HRMS (ESI) m/z: $[M + Na]^+$ calcd for $C_{31}H_{28}N_4NaO_4S$ 575.1723, found 575.1724.

(*E*)-1-benzylidene-6-(4-fluorophenyl)-2-tosyl-2,3,3a,8a,9,10,11,12-octahydro-1*H*,5*H*-pyrrolo[3,4-*c*][1,2,4]triazolo[1,2-*a*]cinnoline-5,7(6*H*)-dione (3w)

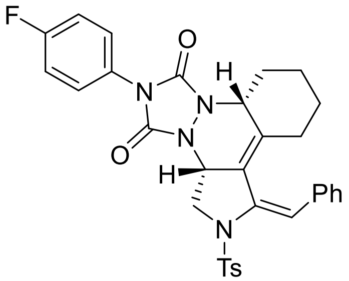

3w

The reaction was conducted with *N*-(3-cyclohexylideneallyl)-4-methyl-*N*-(phenylethynyl)benzenesulfonamide (**1a**, 0.2 mmol, 78.4 mg), 4-(4-fluorophenyl)-3*H*-

1,2,4-triazole-3,5(4*H*)-dione **2b** (0.3 mmol, 57.9 mg), and Cu(OTf)$_2$ (0.02 mmol, 7.2 mg) in DCE (4.0 mL) at room temperature. Purification by column chromatography on silica gel (petroleum ether/ethyl acetate = 5:1) yielded **3w** (65.5 mg, 56%) as a pale yellow oil.
^{1}H NMR (400 MHz, CDCl$_3$) δ 7.79 (d, *J* = 7.1 Hz, 2H), 7.50 – 7.47 (m, 2H), 7.30 – 7.26 (m, 3H), 7.22 – 7.14 (m, 6H), 4.63 (t, *J* = 8.8 Hz, 1H), 4.22 – 4.15 (m, 2H), 3.48 (t, *J* = 9.9 Hz, 1H), 2.39 (s, 3H), 2.29 (d, *J* = 11.0 Hz, 1H), 2.04 (d, *J* = 13.7 Hz, 1H), 1.66 (d, *J* = 13.4 Hz, 1H), 1.46 (d, *J* = 11.9 Hz, 1H), 1.33 (d, *J* = 12.6 Hz, 1H), 1.19 – 1.04 (m, 3H), 0.67 – 0.58 (m, 1H); ^{13}C NMR (100 MHz, CDCl$_3$) δ 161.9 (d, *J* = 247.0 Hz), 154.5, 150.2, 144.5, 136.5, 134.4 (d, *J* = 33.0 Hz), 132.8, 129.7, 128.6, 128.1, 127.6, 127.1, 127.0, 126.9, 126.8 (d, *J* = 3.0 Hz), 122.4, 116.2 (d, *J* = 23.0 Hz), 115.5, 55.5, 54.6, 53.4, 31.3, 30.6, 26.6, 23.7, 21.5; IR (neat): 2927, 1773, 1719, 1654, 1557, 1512, 1420, 1349, 1276, 1165, 1037, 978, 846, 750, 600, 460; HRMS (ESI) m/z: $[M + H]^+$ calcd for $C_{32}H_{30}FN_4O_4S$ 585.1966, found 585.1966.

(*E*)-1-benzylidene-6-(4-chlorophenyl)-2-tosyl-2,3,3a,8a,9,10,11,12-octahydro-1*H*,5*H*-pyrrolo[3,4-*c*][1,2,4]triazolo[1,2-*a*]cinnoline-5,7(6*H*)-dione (3x)

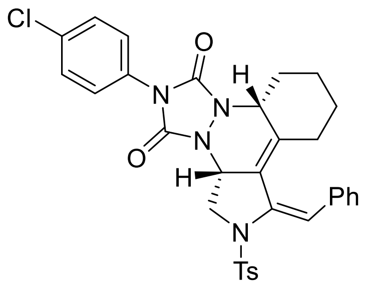

3x

The reaction was conducted with *N*-(3-cyclohexylideneallyl)-4-methyl-*N*-(phenylethynyl)benzenesulfonamide (**1a**, 0.2 mmol, 78.4 mg), 4-(4-chlorophenyl)-3*H*-1,2,4-triazole-3,5(4*H*)-dione **2c** (0.3 mmol, 63.0 mg), and Cu(OTf)$_2$ (0.02 mmol, 7.2 mg) in DCE (4.0 mL) at room temperature. Purification by column chromatography on silica gel (petroleum ether/ethyl acetate = 5:1) yielded **3w** (58.9 mg, 49%) as a pale yellow oil.
^{1}H NMR (400 MHz, CDCl$_3$) δ 7.79 (d, *J* = 6.7 Hz, 2H), 7.50 – 7.44 (m, 4H), 7.30 – 7.26 (m, 3H), 7.22 – 7.18 (m, 4H), 4.63 (t, *J* = 8.8 Hz, 1H), 4.22 – 4.18 (m, 2H), 3.48 (t, *J* = 9.9 Hz, 1H), 2.39 (s, 3H), 2.30 (d, *J* = 11.5 Hz, 1H), 2.04 (d, *J* = 14.0 Hz, 1H), 1.67 (d, *J*

= 13.3 Hz, 1H), 1.47 (d, *J* = 11.9 Hz, 1H), 1.33 (d, *J* = 9.9 Hz, 1H), 1.18 (d, *J* = 12.6 Hz, 1H), 1.11 – 1.04 (m, 1H), 0.88 – 0.84 (m, 1H), 0.68 – 0.58 (m, 1H); ^{13}C NMR (100 MHz, $CDCl_3$) δ 154.2, 149.9, 144.5, 136.5, 134.5, 134.2, 134.0, 132.8, 129.7, 129.5, 129.4, 128.6, 128.1, 127.6, 127.0, 126.2, 122.4, 115.5, 55.5, 54.6, 53.4, 31.3, 30.6, 26.6, 23.7, 21.6; IR (neat): 2925, 1780, 1715, 1645, 1594, 1496, 1417, 1347, 1235, 1165, 1090, 1014, 814, 663, 588, 541; HRMS (ESI) m/z: $[M + H]^+$ calcd for $C_{32}H_{30}ClN_4O_4S$ 601.1671, found 601.1673.

(*E*)-1-benzylidene-2-tosyl-6-(4-(trifluoromethyl)phenyl)-2,3,3a,8a,9,10,11,12-octahydro-1*H*,5*H*-pyrrolo[3,4-*c*][1,2,4]triazolo[1,2-*a*]cinnoline-5,7(6*H*)-dione (3y)

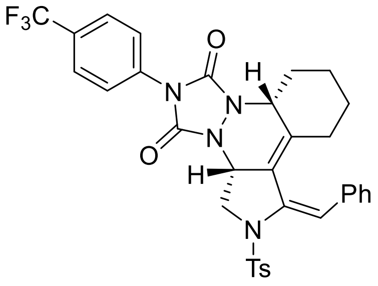

3y

The reaction was conducted with *N*-(3-cyclohexylideneallyl)-4-methyl-*N*-(phenylethynyl)benzenesulfonamide (**1a**, 0.2 mmol, 78.4 mg), 4-(4-(trifluoromethyl)phenyl)-3*H*-1,2,4-triazole-3,5(4*H*)-dione **2c** (0.3 mmol, 72.9 mg), and $Cu(OTf)_2$ (0.02 mmol, 7.2 mg) in DCE (4.0 mL) at room temperature. Purification by column chromatography on silica gel (petroleum ether/ethyl acetate = 5:1) yielded **3y** (77.5 mg, 61%) as a pale yellow oil.

^{1}H NMR (400 MHz, $CDCl_3$) δ 7.79 (d, *J* = 6.2 Hz, 2H), 7.74 – 7.69 (m, 4H), 7.30 – 7.26 (m, 3H), 7.22 – 7.16 (m, 4H), 4.64 (t, *J* = 8.0 Hz, 1H), 4.24 – 4.17 (m, 2H), 3.48 (t, *J* = 11.0 Hz, 1H), 2.39 (s, 3H), 2.31 (d, *J* = 6.5 Hz, 1H), 2.06 – 2.04 (m, 1H), 1.67 (d, *J* = 13.8 Hz, 1H), 1.47 (d, *J* = 13.1 Hz, 1H), 1.33 (d, *J* = 13.2 Hz, 1H), 1.20 – 1.05 (m, 3H), 0.67 – 0.58 (m, 1H); ^{13}C NMR (100 MHz, $CDCl_3$) δ 154.0, 149.6, 144.5, 136.4, 134.4, 134.2, 132.7, 129.8, 128.6, 128.1, 127.6, 127.0, 126.3 (q, J = 3.7 Hz), 124.8, 122.3, 115.6, 55.5, 54.6, 53.3, 31.3, 30.6, 26.5, 23.7, 21.5; IR (neat): 2927, 1718, 1654, 1618, 1492,

1410, 1320, 1163, 1122, 1065, 1018, 843, 661, 591, 492, 405; HRMS (ESI) m/z: $[M + H]^+$ calcd for $C_{33}H_{30}F_3N_4O_4S$ 635.1934, found 635.1934.

(*E*)-1-benzylidene-6-(*p*-tolyl)-2-tosyl-2,3,3a,8a,9,10,11,12-octahydro-1*H*,5*H*-pyrrolo[3,4-*c*][1,2,4]triazolo[1,2-*a*]cinnoline-5,7(6*H*)-dione (3z)

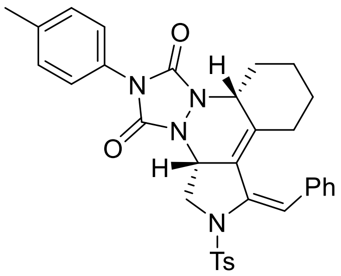

3z

The reaction was conducted with *N*-(3-cyclohexylideneallyl)-4-methyl-*N*-(phenylethynyl)benzenesulfonamide (**1a**, 0.2 mmol, 78.4 mg), 4-(*p*-tolyl)-3*H*-1,2,4-triazole-3,5(4*H*)-dione **2d** (0.3 mmol, 56.7 mg), and $Cu(OTf)_2$ (0.02 mmol, 7.2 mg) in DCE (4.0 mL) at room temperature. Purification by column chromatography on silica gel (petroleum ether/ethyl acetate = 5:1) yielded **3z** (82.5 mg, 71%) as a pale yellow oil.

^1H NMR (400 MHz, $CDCl_3$) δ 7.72 (d, *J* = 8.1 Hz, 2H), 7.27 (d, *J* = 7.1 Hz, 2H), 7.22 – 7.19 (m, 5H), 7.15 – 7.08 (m, 4H), 4.59 – 4.54 (m, 1H), 4.15 – 4.08 (m, 2H), 3.41 (t, *J* = 10.0 Hz, 1H), 2.32 (s, 3H), 2.31 (s, 3H), 2.23 (d, *J* = 10.7 Hz, 1H), 1.96 (d, *J* = 10.9 Hz, 1H), 1.59 (d, *J* = 12.3 Hz, 1H), 1.39 (d, *J* = 9.6 Hz, 1H), 1.26 – 1.21 (m, 2H), 1.11 (d, *J* = 13.3 Hz, 1H), 0.99 (t, *J* = 13.5 Hz, 1H), 0.60 – 0.50 (m, 1H); ^{13}C NMR (100 MHz, $CDCl_3$) δ 154.8, 150.5, 144.4, 138.5, 136.5, 134.7, 134.2, 132.9, 129.8, 129.7, 128.6, 128.1, 127.6, 126.9, 125.2, 122.5, 115.4, 55.4, 54.6, 53.4, 31.3, 30.5, 26.6, 23.7, 21.5, 21.2; IR (neat): 2925, 1776, 1715, 1646, 1517, 1417, 1352, 1288, 1165, 1133, 1035, 810, 697, 665, 589, 545; HRMS (ESI) m/z: $[M + H]^+$ calcd for $C_{33}H_{33}N_4O_4S$ 581.2217, found 581.2217.

(*E*)-1-benzylidene-6-(4-methoxyphenyl)-2-tosyl-2,3,3a,8a,9,10,11,12-octahydro-1*H*,5*H*-pyrrolo[3,4-*c*][1,2,4]triazolo[1,2-*a*]cinnoline-5,7(6*H*)-dione (3aa)

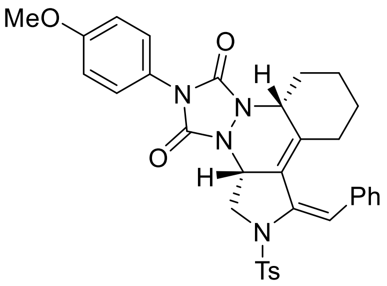

3aa

The reaction was conducted with *N*-(3-cyclohexylideneallyl)-4-methyl-*N*-(phenylethynyl)benzenesulfonamide (**1a**, 0.2 mmol, 78.4 mg), 4-(4-methoxyphenyl)-3*H*-1,2,4-triazole-3,5(4*H*)-dione **2e** (0.3 mmol, 61.5 mg), and $Cu(OTf)_2$ (0.02 mmol, 7.2 mg) in DCE (4.0 mL) at room temperature. Purification by column chromatography on silica gel (petroleum ether/ethyl acetate = 5:1) yielded **3aa** (46.6 mg, 39%) as a pale yellow oil. ^1H NMR (400 MHz, $CDCl_3$) δ 7.70 – 7.66 (m, 4H), 7.38 – 7.32 (m, 4H), 7.28 – 7.23 (m, 3H), 6.97 (d, *J* = 8.4 Hz, 2H), 6.43 (s, 1H), 4.43 – 4.34 (m, 2H), 4.03 (t, *J* = 7.5 Hz, 1H), 3.82 (s, 3H), 3.77 – 3.72 (m, 1H), 3.18 (d, *J* = 13.6 Hz, 1H), 2.40 (s, 3H), 2.07 – 2.01 (m, 1H), 1.78 (d, *J* = 11.9 Hz, 2H), 1.60 – 1.53 (m, 1H), 1.08 – 0.86 (m, 3H); ^{13}C NMR (100 MHz, $CDCl_3$) δ 159.3, 155.3, 150.1, 144.2, 135.6, 134.2, 131.5, 130.5, 129.7, 129.3, 128.3, 127.9, 127.8, 126.8, 125.3, 123.4, 123.2, 114.5, 55.5, 55.0, 54.8, 52.1, 30.5, 29.2, 26.2, 23.9, 21.6; IR (neat): 2926, 1771, 1715, 1635, 1516, 1418, 1351, 1302, 1252, 1165, 1135, 1090, 1033, 780, 664, 589, 544; HRMS (ESI) m/z: $[M + H]^+$ calcd for $C_{33}H_{33}N_4O_5S$ 597.2166, found 597.2166.

(*E*)-1-benzylidene-2-tosyl-6-(4-(trifluoromethoxy)phenyl)-2,3,3a,8a,9,10,11,12-octahydro-1*H*,5*H*-pyrrolo[3,4-*c*][1,2,4]triazolo[1,2-*a*]cinnoline-5,7(6*H*)-dione (3ab)

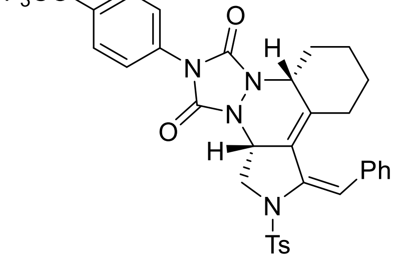

3ab

The reaction was conducted with *N*-(3-cyclohexylideneallyl)-4-methyl-*N*-(phenylethynyl)benzenesulfonamide (**1a**, 0.2 mmol, 78.4 mg), 4-(4-(trifluoromethoxy)phenyl)-3*H*-1,2,4-triazole-3,5(4*H*)-dione **2f** (0.3 mmol, 77.7 mg), and $Cu(OTf)_2$ (0.02 mmol, 7.2 mg) in DCE (4.0 mL) at room temperature. Purification by column chromatography on silica gel (petroleum ether/ethyl acetate = 5:1) yielded **3ab** (84.6 mg, 65%) as a pale yellow oil.

^{1}H NMR (400 MHz, $CDCl_3$) δ 7.79 (d, *J* = 6.4 Hz, 2H), 7.58 (d, *J* = 6.8 Hz, 2H), 7.33 – 7.28 (m, 5H), 7.22 – 7.14 (m, 4H), 4.64 (t, *J* = 8.0 Hz, 1H), 4.23 – 4.18 (m, 2H), 3.48 (t, *J* = 10.0 Hz, 1H), 2.39 (s, 3H), 2.29 (d, *J* = 11.0 Hz, 1H), 2.04 (d, *J* = 14.0 Hz, 1H), 1.67 (d, *J* = 13.4 Hz, 1H), 1.46 (d, *J* = 12.5 Hz, 1H), 1.33 (d, *J* = 11.0 Hz, 1H), 1.18 (d, *J* = 12.2 Hz, 1H), 1.10 – 1.04 (m, 1H), 0.88 – 0.84 (m, 1H), 0.67 – 0.58 (m, 1H); ^{13}C NMR (100 MHz, $CDCl_3$) δ 154.2, 149.9, 148.4, 144.5, 136.4, 134.5, 134.2, 132.8, 129.8, 129.4, 128.6, 128.1, 127.6, 127.0, 126.4, 122.3, 121.7, 115.6, 55.5, 54.6, 53.4, 31.3, 30.6, 26.6, 23.6, 21.6; IR (neat): 2927, 1771, 1718, 1654, 1618, 1510, 1416, 1355, 1263, 1165, 1043, 982, 857, 751, 592, 465; HRMS (ESI) m/z: $[M + Na]^+$ calcd for $C_{33}H_{29}F_3N_4NaO_5S$ 673.1703, found 673.1700.

(*E*)-1-benzylidene-6-(3-chlorophenyl)-2-tosyl-2,3,3a,8a,9,10,11,12-octahydro-1*H*,5*H*-pyrrolo[3,4-*c*][1,2,4]triazolo[1,2-*a*]cinnoline-5,7(6*H*)-dione (3ac)

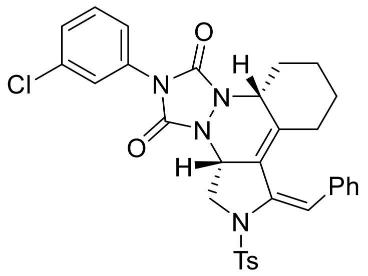

3ac

The reaction was conducted with *N*-(3-cyclohexylideneallyl)-4-methyl-*N*-(phenylethynyl)benzenesulfonamide (**1a**, 0.2 mmol, 78.4 mg), 4-(3-chlorophenyl)-3*H*-1,2,4-triazole-3,5(4*H*)-dione **2g** (0.3 mmol, 63.0 mg), and $Cu(OTf)_2$ (0.02 mmol, 7.2 mg) in DCE (4.0 mL) at room temperature. Purification by column chromatography on silica gel (petroleum ether/ethyl acetate = 5:1) yielded **3ac** (67.3 mg, 56%) as a pale yellow oil.

^{1}H NMR (400 MHz, $CDCl_3$) δ 7.79 (d, *J* = 7.1 Hz, 2H), 7.57 (s, 1H), 7.46 – 7.35 (m, 3H), 7.30 – 7.26 (m, 3H), 7.22 – 7.16 (m, 4H), 4.63 (t, *J* = 9.0 Hz, 1H), 4.22 – 4.15 (m, 2H), 3.47 (t, *J* = 10.0 Hz, 1H), 2.39 (s, 3H), 2.29 (d, *J* = 11.2 Hz, 1H), 2.04 (d, *J* = 13.8 Hz, 1H), 1.67 (d, *J* = 13.4 Hz, 1H), 1.46 (d, *J* = 12.8 Hz, 1H), 1.33 (d, *J* = 13.1 Hz, 1H), 1.21 – 1.16 (m, 1H), 1.07 (t, *J* = 13.9 Hz, 1H), 0.92 – 0.86 (m, 1H), 0.67 – 0.58 (m, 1H); ^{13}C NMR (100 MHz, $CDCl_3$) δ 154.1, 149.8, 144.5, 136.4, 134.8, 134.5, 134.2, 132.8, 132.0, 130.1, 129.8, 128.6, 128.4, 128.1, 127.6, 127.0, 125.2, 123.0, 122.4, 115.5, 55.5, 54.6, 53.3, 31.3, 30.6, 26.6, 23.6, 21.5; IR (neat): 2926, 1776, 1715, 1596, 1486, 1440, 1410, 1351, 1291, 1165, 1090, 1040, 982, 813, 664, 543; HRMS (ESI) m/z: $[M + H]^+$ calcd for $C_{32}H_{30}ClN_4O_4S$ 601.1671, found 601.1671.

(*E*)-1-benzylidene-6-(*m*-tolyl)-2-tosyl-2,3,3a,8a,9,10,11,12-octahydro-1*H*,5*H*-pyrrolo[3,4-*c*][1,2,4]triazolo[1,2-*a*]cinnoline-5,7(6*H*)-dione (3ad)

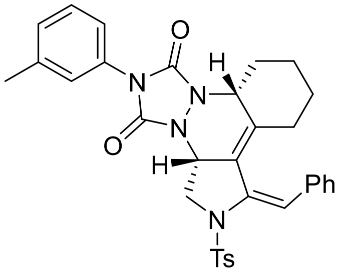

3ad

The reaction was conducted with *N*-(3-cyclohexylideneallyl)-4-methyl-*N*-(phenylethynyl)benzenesulfonamide (**1a**, 0.2 mmol, 78.4 mg), 4-(*m*-tolyl)-3*H*-1,2,4-triazole-3,5(4*H*)-dione **2h** (0.3 mmol, 56.7 mg), and $Cu(OTf)_2$ (0.02 mmol, 7.2 mg) in DCE (4.0 mL) at room temperature. Purification by column chromatography on silica gel (petroleum ether/ethyl acetate = 5:1) yielded **3ad** (69.7 mg, 60%) as a pale yellow oil.

^{1}H NMR (400 MHz, $CDCl_3$) δ 7.79 (d, *J* = 8.2 Hz, 2H), 7.38 – 7.34 (m, 2H), 7.30 – 7.26 (m, 5H), 7.23 – 7.18 (m, 4H), 4.66 – 4.62 (m, 1H), 4.22 – 4.15 (m, 2H), 3.48 (t, *J* = 10.0 Hz, 1H), 2.40 (s, 6H), 2.30 (d, *J* = 10.4 Hz, 1H), 2.04 (d, *J* = 13.9 Hz, 1H), 1.66 (d, *J* = 13.9 Hz, 1H), 1.46 (d, *J* = 10.1 Hz, 1H), 1.33 (d, *J* = 13.5 Hz, 1H), 1.21 – 1.16 (m, 1H), 1.06 (d, *J* = 13.2 Hz, 1H), 0.91 – 0.85 (m, 1H), 0.68 – 0.58 (m, 1H); ^{13}C NMR (100 MHz, $CDCl_3$) δ 154.7, 150.4, 144.4, 139.3, 136.5, 134.7, 134.2, 132.9, 130.6, 129.7, 129.2,

129.0, 128.6, 128.1, 127.6, 126.9, 125.9, 122.4, 122.4, 115.4, 55.5, 54.6, 53.4, 31.3, 30.5, 26.6, 23.7, 21.5, 21.3; IR (neat): 2929, 1778, 1719, 1654, 1597, 1496, 1414, 1351, 1276, 1166, 1088, 1040, 886, 750, 664, 591, 544; HRMS (ESI) m/z: $[M + H]^+$ calcd for $C_{33}H_{33}N_4O_4S$ 581.2217, found 581.2216.

(*E*)-7-benzylidene-5,6-dimethyl-2-phenyl-8-tosyl-7,8,9,9a-tetrahydro-1*H*,5*H*-pyrrolo[3,4-*c*][1,2,4]triazolo[1,2-*a*]pyridazine-1,3(2*H*)-dione (3ae)

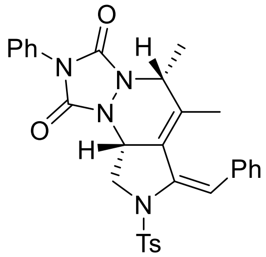

3ae

The reaction was conducted with 4-methyl-*N*-(4-methylpenta-2,3-dien-1-yl)-*N*-(phenylethynyl)benzenesulfonamide (**1w**, 0.2 mmol, 70.2 mg), 4-phenyl-3*H*-1,2,4-triazole-3,5(4*H*)-dione **2a** (0.3 mmol, 52.5 mg), and $Cu(OTf)_2$ (0.02 mmol, 7.2 mg) in DCE (4.0 mL) at room temperature. Purification by column chromatography on silica gel (petroleum ether/ethyl acetate = 5:1) yielded **3ae** (73.6 mg, 68%) as a pale yellow oil.

^1H NMR (400 MHz, $CDCl_3$) δ 7.71 (d, *J* = 8.3 Hz, 2H), 7.41 – 7.39 (m, 4H), 7.33 – 7.31 (m, 1H), 7.23 – 7.19 (m, 4H), 7.13 – 7.10 (m, 3H), 4.62 – 4.57 (m, 1H), 4.13 – 4.05 (m, 2H), 3.62 (d, *J* = 17.2 Hz, 1H), 3.49 – 3.44 (m, 1H), 2.34 (s, 3H), 1.04 (s, 3H); ^{13}C NMR (100 MHz, $CDCl_3$) δ 153.8, 151.4, 144.5, 136.9, 134.5, 133.2, 130.7, 129.8, 129.2, 128.7, 128.4, 128.3, 127.5, 127.3, 126.9, 125.2, 125.1, 115.5, 54.1, 53.1, 47.3, 21.6, 18.4; IR (neat): 2926, 1775, 1716, 1640, 1597, 1501, 1415, 1289, 1168, 1076, 1054, 906, 764, 594,; HRMS (ESI) m/z: $[M + H]^+$ calcd for $C_{30}H_{29}N_4O_4S$ 541.1904, found 541.1904.

(*E*)-1-benzylidene-2-tosyl-2,3,3a,5a,6,7,8,9-octahydro-1*H*-benzo[*e*]isoindole-4,4,5,5-tetracarbonitrile (3ag)

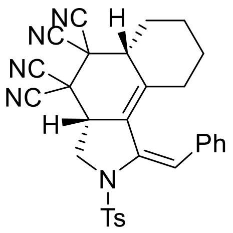

3ag

The reaction was conducted with *N*-(3-cyclohexylideneallyl)-4-methyl-*N*-(phenylethynyl)benzenesulfonamide (**1a**, 0.2 mmol, 78.4 mg), ethene-1,1,2,2-tetracarbonitrile **2j** (0.3 mmol, 38.4 mg), and $Cu(OTf)_2$ (0.02 mmol, 7.2 mg) in DCE (4.0 mL) at room temperature. Purification by column chromatography on silica gel (petroleum ether/ethyl acetate = 5:1) yielded **3ag** (54.1 mg, 52%) as a pale yellow oil.

^{1}H NMR (400 MHz, $CDCl_3$) δ 7.72 (d, *J* = 7.7 Hz, 2H), 7.65 (d, *J* = 8.0 Hz, 2H), 7.38 – 7.35 (m, 2H), 7.31 – 7.26 (m, 3H), 6.55 (s, 1H), 4.32 – 4.27 (m, 1H), 3.63 (t, *J* = 10.9 Hz, 1H), 3.25 (d, *J* = 14.2 Hz, 1H), 3.05 – 3.00 (m, 2H), 2.37 (s, 3H), 2.31 (d, *J* = 9.4 Hz, 1H), 2.11 – 1.98 (m, 2H), 1.91 – 1.84 (m, 2H), 1.60 – 1.54 (m, 1H), 1.26 – 1.18 (m, 1H); ^{13}C NMR (100 MHz, $CDCl_3$) δ 145.6, 134.8, 133.3, 132.1, 130.2, 129.8, 129.6, 128.6, 128.2, 127.9, 126.2, 124.3, 111.0, 110.2, 109.5, 108.7, 50.3, 45.9, 43.4, 41.0, 39.1, 31.8, 30.9, 25.9, 25.6, 21.6; IR (neat): 2926, 1774, 1619, 1604, 1543, 1421, 1389, 1221, 1105, 1046, 889, 782, 637, 586, 543; HRMS (ESI) m/z: $[M + H]^+$ calcd for $C_{30}H_{26}N_5O_2S$ 520.1802, found 520.1802.

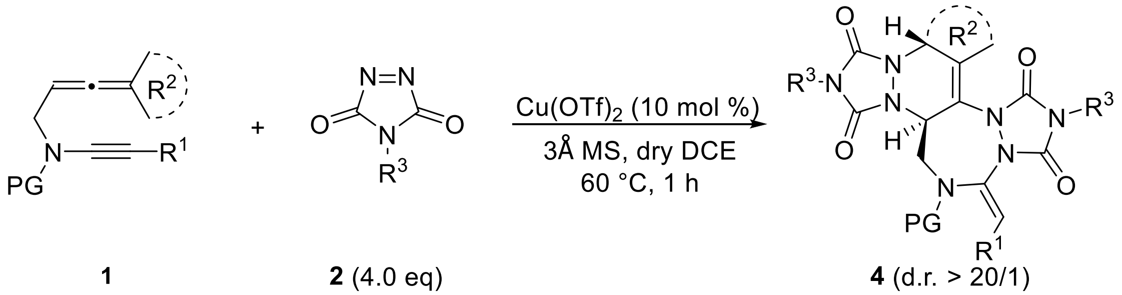

Supplementary Figure 95. Synthesis of pentacyclic triazepanes **4**.

General procedure for the synthesis of pentacyclic triazepanes 4:

PTAD (*cis*-diazenes) **2** (0.8 mmol), 3Å molecular sieves (40 mg), and $Cu(OTf)_2$ (0.02 mmol, 7.2 mg) were added in this order to the allenynes **1** (0.2 mmol) in dry DCE (4.0 mL) at room temperature. The reaction mixture was stirred at 60 °C (60 °C, heating

mantle temperature) and the progress of the reaction was monitored by TLC. The reaction typically took 1 h. Upon completion, the mixture was then concentrated and the residue was purified by chromatography on silica gel (eluent: petroleum ether/ethyl acetate) to afford the desired products **4**.

(*Z*)-7-benzylidene-2,10-diphenyl-6-tosyl-4a,6,7,13,14,15,16,16a-octahydro-1*H*,5*H*,9*H*-[1,2,4]triazolo[1',2':1,2][1,2,4]triazepino[6,7-*c*][1,2,4]triazolo[1,2-*a*]cinnoline-1,3,9,11(2*H*,10*H*)-tetraone (4a)

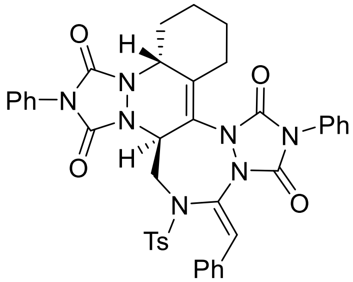

4a

The reaction was conducted with *N*-(3-cyclohexylideneallyl)-4-methyl-*N*-(phenylethynyl)benzenesulfonamide **1a** (0.2 mmol, 78.4 mg), 4-phenyl-3*H*-1,2,4-triazole-3,5(4*H*)-dione **2a** (0.8 mmol, 140.0 mg), 3Å molecular sieves (40 mg), and $Cu(OTf)_2$ (0.02 mmol, 7.2 mg) in dry DCE (4.0 mL) at 60 ^oC (60 °C, heating mantle temperature). Purification by column chromatography on silica gel (petroleum ether/ethyl acetate = 5:1) yielded **4a** (112.8 mg, 76%) as a pale yellow oil.

^1H NMR (400 MHz, $CDCl_3$) δ 7.65 – 7.61 (m, 4H), 7.58 – 7.54 (m, 2H), 7.52 – 7.47 (m, 3H), 7.44 – 7.37 (m, 2H), 7.17 – 7.14 (m, 1H), 7.07 – 7.03 (m, 4H), 6.97 (d, *J* = 8.1 Hz, 2H), 6.73 (s, 1H), 5.05 – 5.01 (m, 1H), 4.86 – 4.81 (m, 1H), 4.48 – 4.44 (m, 1H), 3.09 – 3.03 (m, 1H), 2.66 (d, *J* = 13.4 Hz, 1H), 2.49 (d, *J* = 8.5 Hz, 1H), 2.21 (s, 3H), 2.00 – 1.95 (m, 3H), 1.75 – 1.58 (m, 4H); ^{13}C NMR (100 MHz, $CDCl_3$) δ 151.2, 151.0, 150.7, 147.5, 146.8, 144.5, 135.7, 131.4, 131.3, 130.9, 130.8, 129.5, 129.2, 129.1(4), 129.0(9), 128.6, 128.5, 128.3, 128.2, 127.5, 126.1, 125.5, 125.0, 118.5, 57.1, 54.5, 49.8, 30.5, 30.4, 27.2, 24.3, 21.3; IR (neat): 2924, 1771, 1722, 1646, 1502, 1453, 1417, 1361, 1273, 1164, 1086, 963, 736, 690, 611, 511; HRMS (ESI) m/z: $[M + Na]^+$ calcd for $C_{40}H_{35}N_7NaO_6S$ 764.2262, found 764.2266.

(*Z*)-7-benzylidene-6-((4-methoxyphenyl)sulfonyl)-2,10-diphenyl-4a,6,7,13,14,15,16,16a-octahydro-1*H*,5*H*,9*H*-[1,2,4]triazolo[1',2':1,2][1,2,4]triazepino[6,7-*c*][1,2,4]triazolo[1,2-*a*]cinnoline-1,3,9,11(2*H*,10*H*)-tetraone (4b)

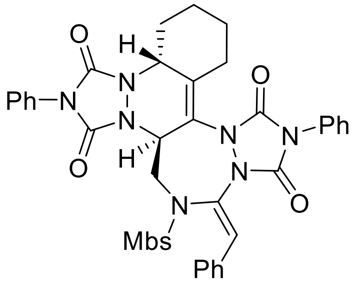

4b

The reaction was conducted with *N*-(3-cyclohexylideneallyl)-4-methoxy-*N*-(phenylethynyl)benzenesulfonamide **1b** (0.2 mmol, 81.6 mg), 4-phenyl-3*H*-1,2,4-triazole-3,5(4*H*)-dione **2a** (0.8 mmol, 140.0 mg), 3Å molecular sieves (40 mg), and $Cu(OTf)_2$ (0.02 mmol, 7.2 mg) in dry DCE (4.0 mL) at 60 ºC (60 °C, heating mantle temperature). Purification by column chromatography on silica gel (petroleum ether/ethyl acetate = 5:1) yielded **4b** (112.2 mg, 74%) as a pale yellow oil.

^{1}H NMR (400 MHz, $CDCl_3$) δ 7.68 (d, *J* = 9.0 Hz, 2H), 7.63 – 7.47 (m, 8H), 7.44 – 7.37 (m, 2H), 7.18 – 7.15 (m, 1H), 7.12 – 7.09 (m, 3H), 6.73 (s, 1H), 6.63 (d, *J* = 9.0 Hz, 2H), 5.05 – 5.01 (m, 1H), 4.86 – 4.81 (m, 1H), 4.48 – 4.44 (m, 1H), 3.69 (s, 3H), 3.09 – 3.03 (m, 1H), 2.67 (d, *J* = 13.6 Hz, 1H), 2.50 (d, *J* = 10.9 Hz, 1H), 2.03 – 1.95 (m, 3H), 1.79 – 1.65 (m, 4H); ^{13}C NMR (100 MHz, $CDCl_3$) δ 163.5, 151.2, 151.0, 150.8, 147.5, 146.8, 131.4(4), 131.3(6), 131.0, 130.8, 130.1, 129.8, 129.2(4), 129.1(6), 128.6, 128.5, 128.4, 128.3, 126.1, 125.7, 125.0, 118.6, 114.0, 57.2, 55.5, 54.6, 49.8, 30.5, 30.4, 27.2, 24.3; IR (neat): 2924, 1769, 1716, 1647, 1598, 1500, 1417, 1359, 1264, 1159, 1088, 961, 736, 691, 611, 557; HRMS (ESI) m/z: $[M + H]^+$ calcd for $C_{40}H_{36}N_7O_7S$ 758.2391, found 758.2388.

(*Z*)-7-benzylidene-2,10-diphenyl-6-(phenylsulfonyl)-4a,6,7,13,14,15,16,16a-octahydro-1*H*,5*H*,9*H*-[1,2,4]triazolo[1',2':1,2][1,2,4]triazepino[6,7-*c*][1,2,4]triazolo[1,2-*a*]cinnoline-1,3,9,11(2*H*,10*H*)-tetraone (4c)

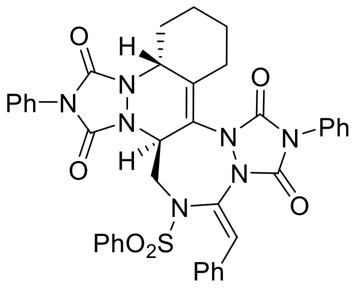

4c

The reaction was conducted with *N*-(3-cyclohexylideneallyl)-*N*-(phenylethynyl)benzenesulfonamide **1c** (0.2 mmol, 75.6 mg), 4-phenyl-3*H*-1,2,4-triazole-3,5(4*H*)-dione **2a** (0.8 mmol, 140.0 mg), 3Å molecular sieves (40 mg), and $Cu(OTf)_2$ (0.02 mmol, 7.2 mg) in dry DCE (4.0 mL) at 60 ^oC (60 °C, heating mantle temperature). Purification by column chromatography on silica gel (petroleum ether/ethyl acetate = 5:1) yielded **4c** (104.8 mg, 72%) as a pale yellow oil.

^1H NMR (400 MHz, $CDCl_3$) δ 7.79 (d, *J* = 7.3 Hz, 2H), 7.63 – 7.47 (m, 7H), 7.44 – 7.39 (m, 2H), 7.33 (t, *J* = 7.5 Hz, 1H), 7.23 – 7.19 (m, 2H), 7.15 – 7.12 (m, 1H), 7.08 – 7.03 (m, 4H), 6.74 (s, 1H), 5.06 – 5.02 (m, 1H), 4.87 – 4.82 (m, 1H), 4.48 – 4.44 (m, 1H), 3.10 – 3.04 (m, 1H), 2.67 (d, *J* = 13.7 Hz, 1H), 2.49 (d, *J* = 11.0 Hz, 1H), 2.04 – 1.95 (m, 3H), 1.72 – 1.62 (m, 4H); ^{13}C NMR (100 MHz, $CDCl_3$) δ 151.2, 151.0, 150.7, 147.6, 146.9, 138.8, 133.5, 131.4, 130.8(0), 130.7(5), 129.4, 129.3, 129.2, 128.9, 128.6, 128.5, 128.4, 128.3, 127.5, 126.1, 125.5, 125.0, 118.5, 57.1, 54.6, 49.9, 30.5, 30.4, 27.2, 24.3; IR (neat): 2929, 1773, 1719, 1598, 1504, 1414, 1357, 1276, 1167, 1088, 1027, 961, 863, 753, 688, 573; HRMS (ESI) m/z: $[M + H]^+$ calcd for $C_{39}H_{34}N_7O_6S$ 728.2286, found 728.2282.

(*Z*)-7-benzylidene-6-((4-bromophenyl)sulfonyl)-2,10-diphenyl-4a,6,7,13,14,15,16,16a-octahydro-1*H*,5*H*,9*H*-[1,2,4]triazolo[1',2':1,2][1,2,4]triazepino[6,7-*c*][1,2,4]triazolo[1,2-*a*]cinnoline-1,3,9,11(2*H*,10*H*)-tetraone (4d)

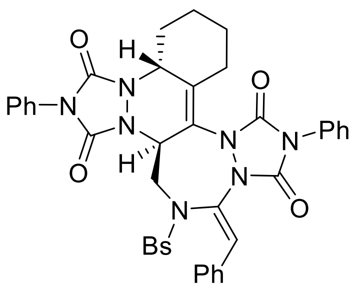

4d

The reaction was conducted with 4-bromo-*N*-(3-cyclohexylideneallyl)-*N*-(phenylethynyl)benzenesulfonamide **1d** (0.2 mmol, 91.2 mg), 4-phenyl-3*H*-1,2,4-triazole-3,5(4*H*)-dione **2a** (0.8 mmol, 140.0 mg), 3Å molecular sieves (40 mg), and $Cu(OTf)_2$ (0.02 mmol, 7.2 mg) in dry DCE (4.0 mL) at 60 ºC (60 °C, heating mantle temperature). Purification by column chromatography on silica gel (petroleum ether/ethyl acetate = 5:1) yielded **4d** (129.1 mg, 80%) as a pale yellow oil.

^{1}H NMR (400 MHz, $CDCl_3$) δ 7.61 – 7.48 (m, 9H), 7.45 – 7.38 (m, 2H), 7.26 – 7.19 (m, 3H), 7.10 – 7.06 (m, 2H), 6.98 (d, *J* = 7.6 Hz, 2H), 6.75 (s, 1H), 5.09 – 5.05 (m, 1H), 4.90 – 4.86 (m, 1H), 4.50 – 4.46 (m, 1H), 3.15 – 3.09 (m, 1H), 2.69 (d, *J* = 13.3 Hz, 1H), 2.51 (d, *J* = 8.7 Hz, 1H), 2.05 – 1.96 (m, 3H), 1.80 – 1.66 (m, 4H); ^{13}C NMR (100 MHz, $CDCl_3$) δ 151.2, 151.1, 151.0, 147.6, 147.2, 137.3, 132.0, 131.4, 130.8, 130.7, 129.3, 129.2, 129.0, 128.9, 128.6, 128.5, 128.3, 126.2, 125.5, 125.0, 118.4, 57.2, 54.9, 50.3, 30.6, 30.5, 27.2, 24.3; IR (neat): 2928, 1773, 1718, 1654, 1561, 1500, 1413, 1341, 1275, 1152, 1031, 957, 865, 765, 587, 506; HRMS (ESI) m/z: $[M + Na]^+$ calcd for $C_{39}H_{32}BrN_7NaO_6S$ 828.1210, found 828.1212.

(*Z*)-7-benzylidene-6-(methylsulfonyl)-2,10-diphenyl-4a,6,7,13,14,15,16,16a-octahydro-1*H*,5*H*,9*H*-[1,2,4]triazolo[1',2':1,2][1,2,4]triazepino[6,7-*c*][1,2,4]triazolo[1,2-*a*]cinnoline-1,3,9,11(2*H*,10*H*)-tetraone (4e)

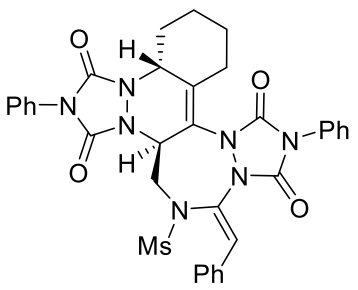

4e

The reaction was conducted with *N*-(3-cyclohexylideneallyl)-*N*-(phenylethynyl)methanesulfonamide **1e** (0.2 mmol, 63.0 mg), 4-phenyl-3*H*-1,2,4-triazole-3,5(4*H*)-dione **2a** (0.8 mmol, 140.0 mg), 3Å molecular sieves (40 mg), and $Cu(OTf)_2$ (0.02 mmol, 7.2 mg) in dry DCE (4.0 mL) at 60 ^oC (60 °C, heating mantle temperature). Purification by column chromatography on silica gel (petroleum ether/ethyl acetate = 5:1) yielded **4e** (102.6 mg, 77%) as a yellow solid (mp 223-225 °C).

^{1}H NMR (400 MHz, $CDCl_3$) δ 7.59 – 7.54 (m, 4H), 7.51 – 7.47 (m, 6H), 7.43 – 7.39 (m, 4H), 6.90 (s, 1H), 5.08 – 5.04 (m, 1H), 4.84 – 4.80 (m, 1H), 4.51 – 4.47 (m, 1H), 3.14 – 3.08 (m, 1H), 2.75 (s, 3H), 2.70 (s, 1H), 2.51 (d, *J* = 11.0 Hz, 1H), 2.07 – 1.96 (m, 3H), 1.80 – 1.66 (m, 4H); ^{13}C NMR (100 MHz, $CDCl_3$) δ 151.3, 150.9, 147.6, 147.3, 131.3, 130.8(4), 130.7(6), 130.1, 129.2(3), 129.1(5), 129.0, 128.9, 128.6, 128.3, 126.4, 126.3, 126.2, 125.3, 124.9, 118.4, 57.2, 54.9, 49.5, 40.6, 30.6, 30.4, 27.3, 24.3; IR (neat): 2924, 1771, 1718, 1637, 1500, 1455, 1413, 1378, 1341, 1269, 1149, 959, 861, 765, 688, 575, 503; HRMS (ESI) m/z: $[M + Na]^+$ calcd for $C_{34}H_{31}N_7NaO_6S$ 688.1949, found 688.1945.

(*Z*)-7-(4-fluorobenzylidene)-2,10-diphenyl-6-tosyl-4a,6,7,13,14,15,16,16a-octahydro-1*H*,5*H*,9*H*-[1,2,4]triazolo[1',2':1,2][1,2,4]triazepino[6,7-*c*][1,2,4]triazolo[1,2-*a*]cinnoline-1,3,9,11(2*H*,10*H*)-tetraone (4f)

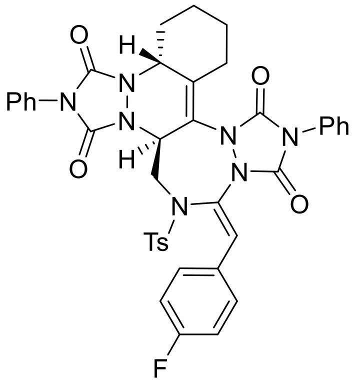

4f

The reaction was conducted with *N*-(3-cyclohexylideneallyl)-*N*-((4-fluorophenyl)ethynyl)-4-methylbenzenesulfonamide **1f** (0.2 mmol, 82.0 mg), 4-phenyl-3*H*-1,2,4-triazole-3,5(4*H*)-dione **2a** (0.8 mmol, 140.0 mg), 3Å molecular sieves (40 mg), and $Cu(OTf)_2$ (0.02 mmol, 7.2 mg) in dry DCE (4.0 mL) at 60 ^{o}C (60 °C, heating mantle temperature). Purification by column chromatography on silica gel (petroleum ether/ethyl acetate = 5:1) yielded **4f** (132.2 mg, 87%) as a pale yellow oil.

^{1}H NMR (400 MHz, $CDCl_3$) δ 7.65 – 7.50 (m, 9H), 7.42 – 7.38 (m, 2H), 7.08 – 7.00 (m, 4H), 6.78 – 6.70 (m, 3H), 5.03 – 4.99 (m, 1H), 4.85 – 4.81 (m, 1H), 4.46 (d, *J* = 8.1 Hz, 1H), 3.03 (t, *J* = 12.0 Hz, 1H), 2.65 (d, *J* = 12.5 Hz, 1H), 2.49 (d, *J* = 9.2 Hz, 1H), 2.25 (s, 3H), 2.02 – 1.95 (m, 3H), 1.74 – 1.62 (m, 4H); ^{13}C NMR (100 MHz, $CDCl_3$) δ 162.9 (d, *J* = 249.0 Hz), 151.1 (d, *J* = 25.0 Hz), 150.7, 147.5, 146.8, 144.7, 135.9, 131.4, 130.8, 130.5 (d, *J* = 9.0 Hz), 129.9, 129.5, 129.2(3), 129.1(6), 128.5, 128.3, 127.5, 127.2 (d, *J* = 4.0 Hz), 126.1, 125.4 (d, *J* = 2.0 Hz), 124.9, 118.5, 115.4 (d, *J* = 22.0 Hz), 57.2, 54.5, 49.8, 30.5, 30.4, 27.1, 24.3, 21.3; IR (neat): 2927, 1771, 1716, 1600, 1504, 1416, 1361, 1276, 1231, 1164, 1086, 961, 863, 735, 686, 608, 547; HRMS (ESI) m/z: $[M + H]^+$ calcd for $C_{40}H_{35}FN_7O_6S$ 760.2348, found 760.2344.

(*Z*)-7-(4-bromobenzylidene)-2,10-diphenyl-6-tosyl-4a,6,7,13,14,15,16,16a-octahydro-1*H*,5*H*,9*H*-[1,2,4]triazolo[1',2':1,2][1,2,4]triazepino[6,7-*c*][1,2,4]triazolo[1,2-*a*]cinnoline-1,3,9,11(2*H*,10*H*)-tetraone (4g)

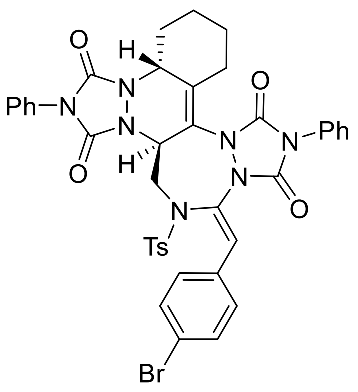

4g

The reaction was conducted with *N*-((4-bromophenyl)ethynyl)-*N*-(3-cyclohexylideneallyl)-4-methylbenzenesulfonamide **1g** (0.2 mmol, 94.0 mg), 4-phenyl-3*H*-1,2,4-triazole-3,5(4*H*)-dione **2a** (0.8 mmol, 140.0 mg), 3Å molecular sieves (40 mg), and $Cu(OTf)_2$ (0.02 mmol, 7.2 mg) in dry DCE (4.0 mL) at 60 ^oC (60 °C, heating mantle temperature). Purification by column chromatography on silica gel (petroleum ether/ethyl acetate = 5:1) yielded **4g** (128.1 mg, 78%) as a pale yellow oil.

^1H NMR (400 MHz, $CDCl_3$) δ 7.62 – 7.58 (m, 5H), 7.52 – 7.48 (m, 4H), 7.42 – 7.38 (m, 2H), 7.16 (d, *J* = 7.6 Hz, 2H), 6.98 (d, *J* = 7.6 Hz, 2H), 6.89 (d, *J* = 7.6 Hz, 2H), 6.67 (s, 1H), 5.07 – 5.03 (m, 1H), 4.86 – 4.82 (m, 1H), 4.47 (d, *J* = 9.3 Hz, 1H), 3.04 (t, *J* = 11.9 Hz, 1H), 2.66 (d, *J* = 13.0 Hz, 1H), 2.50 (d, *J* = 10.7 Hz, 1H), 2.29 (s, 3H), 2.04 – 1.96 (m, 3H), 1.76 – 1.66 (m, 4H); ^{13}C NMR (100 MHz, $CDCl_3$) δ 151.3, 151.0, 150.8, 147.6, 146.9, 145.0, 135.9, 131.5, 131.4, 130.8, 130.0, 129.8, 129.4(9), 129.4(6), 129.3, 129.2, 128.6, 128.3, 127.5, 126.7, 126.1, 125.0, 123.2, 118.6, 57.2, 54.6, 50.1, 30.6, 30.4, 27.2, 24.3, 21.5; IR (neat): 2929, 1771, 1720, 1648, 1596, 1502, 1416, 1359, 1272, 1164, 1071, 961, 810, 736, 690, 647, 609, 514; HRMS (ESI) m/z: $[M + H]^+$ calcd for $C_{40}H_{35}BrN_7O_6S$ 820.1547, found 820.1550.

(*Z*)-2,10-diphenyl-6-tosyl-7-(4-(trifluoromethyl)benzylidene)-4a,6,7,13,14,15,16,16a-octahydro-1*H*,5*H*,9*H*-[1,2,4]triazolo[1',2':1,2][1,2,4]triazepino[6,7-*c*][1,2,4]triazolo[1,2-*a*]cinnoline-1,3,9,11(2*H*,10*H*)-tetraone (4h)

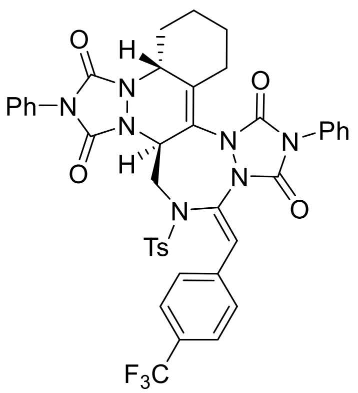

4h

The reaction was conducted with *N*-(3-cyclohexylideneallyl)-4-methyl-*N*-((4-(trifluoromethyl)phenyl)ethynyl)benzenesulfonamide **1h** (0.2 mmol, 92.0 mg), 4-phenyl-3*H*-1,2,4-triazole-3,5(4*H*)-dione **2a** (0.8 mmol, 140.0 mg), 3Å molecular sieves (40 mg), and $Cu(OTf)_2$ (0.02 mmol, 7.2 mg) in dry DCE (4.0 mL) at 60 °C (60 °C, heating mantle temperature). Purification by column chromatography on silica gel (petroleum ether/ethyl acetate = 5:1) yielded **4h** (118.3 mg, 73%) as a pale yellow oil.

^1H NMR (400 MHz, $CDCl_3$) δ 7.62 – 7.50 (m, 9H), 7.43 – 7.38 (m, 2H), 7.28 – 7.22 (m, 2H), 7.11 (d, *J* = 8.1 Hz, 2H), 6.92 (d, *J* = 8.1 Hz, 2H), 6.77 (s, 1H), 5.12 – 5.08 (m, 1H), 4.92 – 4.87 (m, 1H), 4.50 – 4.46 (m, 1H), 3.12 – 3.06 (m, 1H), 2.66 (d, *J* = 13.3 Hz, 1H), 2.51 (d, *J* = 8.9 Hz, 1H), 2.19 (s, 3H), 2.05 – 1.96 (m, 3H), 1.77 – 1.64 (m, 4H); ^{13}C NMR (100 MHz, $CDCl_3$) δ 151.3, 151.0, 150.9, 147.6, 147.0, 144.8, 135.6, 134.6, 131.3, 130.8, 129.4, 129.3, 129.2, 128.8, 128.6(2), 128.5(5), 128.3, 128.2, 127.5, 126.2, 125.04 (q, J = 3.6 Hz), 124.9, 118.7, 57.2, 54.7, 50.2, 30.6, 30.4, 27.2, 24.3, 21.1; IR (neat): 2924, 1771, 1715, 1646, 1500, 1416, 1324, 1269, 1164, 1125, 1068, 963, 865, 645, 545; HRMS (ESI) m/z: $[M + H]^+$ calcd for $C_{41}H_{35}F_3N_7O_6S$ 810.2316, found 810.2317.

(*Z*)-4-((1,3,9,11-tetraoxo-2,10-diphenyl-6-tosyl-2,3,4a,5,6,10,11,13,14,15,16,16a-dodecahydro-1*H*,7*H*,9*H*-[1,2,4]triazolo[1',2':1,2][1,2,4]triazepino[6,7-*c*][1,2,4]triazolo[1,2-*a*]cinnolin-7-ylidene)methyl)benzonitrile (4i)

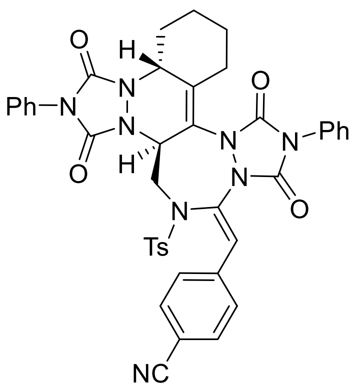

4i

The reaction was conducted with *N*-((4-cyanophenyl)ethynyl)-*N*-(3-cyclohexylideneallyl)-4-methylbenzenesulfonamide **1i** (0.2 mmol, 83.4 mg), 4-phenyl-3*H*-1,2,4-triazole-3,5(4*H*)-dione **2a** (0.8 mmol, 140.0 mg), 3Å molecular sieves (40 mg), and $Cu(OTf)_2$ (0.02 mmol, 7.2 mg) in dry DCE (4.0 mL) at 60 ^{o}C (60 °C, heating mantle temperature). Purification by column chromatography on silica gel (petroleum ether/ethyl acetate = 5:1) yielded **4i** (102.8 mg, 67%) as a pale yellow oil.

^{1}H NMR (400 MHz, $CDCl_3$) δ 7.61 – 7.51 (m, 9H), 7.46 – 7.41 (m, 2H), 7.31 (d, *J* = 7.9 Hz, 2H), 7.13 (d, *J* = 7.9 Hz, 2H), 6.98 (d, *J* = 7.8 Hz, 2H), 6.75 (s, 1H), 5.10 – 5.06 (m, 1H), 4.90 – 4.85 (m, 1H), 4.47 (d, *J* = 7.6 Hz, 1H), 3.06 (t, *J* = 12.0 Hz, 1H), 2.64 (d, *J* = 12.5 Hz, 1H), 2.51 (d, *J* = 8.2 Hz, 1H), 2.28 (s, 3H), 2.04 – 1.96 (m, 3H), 1.77 – 1.62 (m, 4H); ^{13}C NMR (100 MHz, $CDCl_3$) δ 151.2, 151.0, 150.7, 147.6, 147.0, 145.1, 135.8, 135.7, 131.9, 131.2, 130.7, 129.6, 129.3, 129.2, 129.0, 128.8, 128.7, 128.4, 128.1, 127.4, 126.1, 124.9, 118.7, 118.1, 111.9, 57.2, 54.5, 50.2, 30.6, 30.5, 27.1, 24.3, 21.4; IR (neat): 2927, 1774, 1719, 1654, 1502, 1413, 1357, 1280, 1164, 1121, 1072, 961, 863, 690, 586, 465; HRMS (ESI) m/z: [M + H]$^+$ calcd for $C_{41}H_{35}N_8O_6S$ 767.2395, found 767.2396.

methyl (*Z*)-4-((1,3,9,11-tetraoxo-2,10-diphenyl-6-tosyl-2,3,4a,5,6,10,11,13,14,15,16,16a-dodecahydro-1*H*,7*H*,9*H*-[1,2,4]triazolo[1',2':1,2][1,2,4]triazepino[6,7-*c*][1,2,4]triazolo[1,2-*a*]cinnolin-7-ylidene)methyl)benzoate (4j).

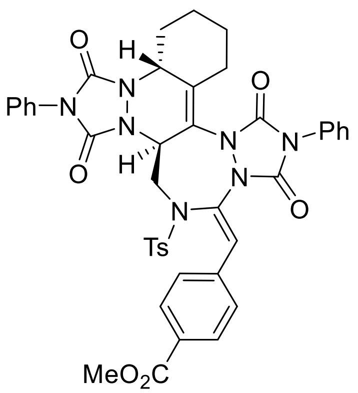

4j

The reaction was conducted with methyl 4-(((*N*-(3-cyclohexylideneallyl)-4-methylphenyl)sulfonamido)ethynyl)benzoate **1j** (0.2 mmol, 90.0 mg), 4-phenyl-3*H*-1,2,4-triazole-3,5(4*H*)-dione **2a** (0.8 mmol, 140.0 mg), 3Å molecular sieves (40 mg), and $Cu(OTf)_2$ (0.02 mmol, 7.2 mg) in dry DCE (4.0 mL) at 60 ºC (60 °C, heating mantle temperature). Purification by column chromatography on silica gel (petroleum ether/ethyl acetate = 5:1) yielded **4j** (113.6 mg, 71%) as a pale yellow oil.

^{1}H NMR (400 MHz, $CDCl_3$) δ 7.68 (d, *J* = 7.9 Hz, 1H), 7.62 – 7.50 (m, 9H), 7.43 – 7.38 (m, 2H), 7.07 (d, *J* = 7.9 Hz, 2H), 6.93 (d, *J* = 7.8 Hz, 2H), 6.77 (s, 1H), 5.11 – 5.08 (m, 1H), 4.89 – 4.85 (m, 1H), 4.48 (d, *J* = 8.0 Hz, 1H), 3.91 (s, 3H), 3.08 (t, *J* = 12.0 Hz, 1H), 2.66 (d, *J* = 12.6 Hz, 1H), 2.51 (d, *J* = 9.3 Hz, 1H), 2.17 (s, 3H), 2.05 – 1.96 (m, 3H), 1.80 – 1.64 (m, 4H); ^{13}C NMR (100 MHz, $CDCl_3$) δ 166.2, 151.3, 151.0, 150.8, 147.7, 146.9, 144.8, 135.8, 135.5, 131.3, 130.8, 130.0, 129.5, 129.4, 129.3, 129.2, 128.6, 128.3, 127.9, 127.4, 126.1, 125.0, 118.7, 57.2, 54.6, 52.2, 50.2, 30.6, 30.4, 27.2, 24.3, 21.2; IR (neat): 2924, 1771, 1716, 1646, 1503, 1416, 1359, 1279, 1164, 1111, 959, 812, 736, 690, 611, 548; HRMS (ESI) m/z: $[M + H]^+$ calcd for $C_{42}H_{38}N_7O_8S$ 800.2497, found 800.2494.

(*Z*)-4-((1,3,9,11-tetraoxo-2,10-diphenyl-6-tosyl-2,3,4a,5,6,10,11,13,14,15,16,16a-dodecahydro-1*H*,7*H*,9*H*-[1,2,4]triazolo[1',2':1,2][1,2,4]triazepino[6,7-*c*][1,2,4]triazolo[1,2-*a*]cinnolin-7-ylidene)methyl)benzaldehyde (4k)

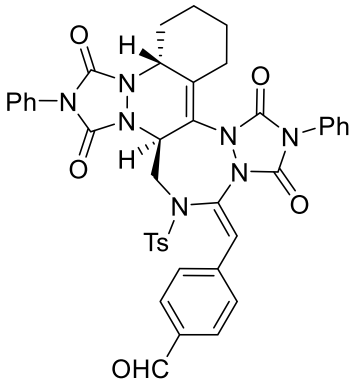

4k

The reaction was conducted with *N*-(3-cyclohexylideneallyl)-*N*-((4-formylphenyl)ethynyl)-4-methylbenzenesulfonamide **1k** (0.2 mmol, 84.0 mg), 4-phenyl-3*H*-1,2,4-triazole-3,5(4*H*)-dione **2a** (0.8 mmol, 140.0 mg), 3Å molecular sieves (40 mg), and $Cu(OTf)_2$ (0.02 mmol, 7.2 mg) in dry DCE (4.0 mL) at 60 ^oC (60 °C, heating mantle temperature). Purification by column chromatography on silica gel (petroleum ether/ethyl acetate = 5:1) yielded **4k** (109.3 mg, 71%) as a pale yellow oil.

^1H NMR (400 MHz, $CDCl_3$) δ 9.92 (s, 1H), 7.62 – 7.51 (m, 11H), 7.46 – 7.39 (m, 2H), 7.19 (d, *J* = 7.7 Hz, 2H), 6.94 (d, *J* = 7.7 Hz, 2H), 6.79 (s, 1H), 5.11 – 5.07 (m, 1H), 4.91 – 4.86 (m, 1H), 4.48 (d, *J* = 8.2 Hz, 1H), 3.08 (t, *J* = 12.1 Hz, 1H), 2.65 (d, *J* = 13.0 Hz, 1H), 2.51 (d, *J* = 9.6 Hz, 1H), 2.17 (s, 3H), 2.05 – 1.97 (m, 3H), 1.77 – 1.58 (m, 4H); ^{13}C NMR (100 MHz, $CDCl_3$) δ 191.1, 151.3, 151.0, 150.7, 147.7, 146.9, 144.9, 137.0, 136.0, 135.8, 131.3, 130.8, 129.5, 129.4, 129.3, 129.2, 128.9, 128.7, 128.5, 128.3, 127.5, 126.1, 125.0, 118.8, 57.2, 54.6, 50.2, 30.6, 30.5, 27.2, 24.3, 21.4; IR (neat): 2928, 1773, 1719, 1654, 1604, 1503, 1413, 1358, 1274, 1212, 1164, 1087, 963, 839, 690, 584, 405; HRMS (ESI) m/z: $[M + Na]^+$ calcd for $C_{41}H_{35}N_7NaO_7S$ 792.2211, found 792.2212.

(*Z*)-7-(4-(*tert*-butyl)benzylidene)-2,10-diphenyl-6-tosyl-4a,6,7,13,14,15,16,16a-octahydro-1*H*,5*H*,9*H*-[1,2,4]triazolo[1',2':1,2][1,2,4]triazepino[6,7-*c*][1,2,4]triazolo[1,2-*a*]cinnoline-1,3,9,11(2*H*,10*H*)-tetraone (4l)

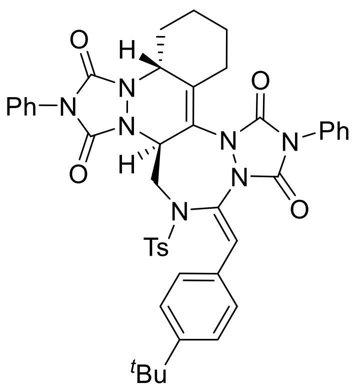

4l

The reaction was conducted with *N*-((4-(*tert*-butyl)phenyl)ethynyl)-*N*-(3-cyclohexylideneallyl)-4-methylbenzenesulfonamide **1l** (0.2 mmol, 89.6 mg), 4-phenyl-3*H*-1,2,4-triazole-3,5(4*H*)-dione **2a** (0.8 mmol, 140.0 mg), 3Å molecular sieves (40 mg), and $Cu(OTf)_2$ (0.02 mmol, 7.2 mg) in dry DCE (4.0 mL) at 60 °C (60 °C, heating mantle temperature). Purification by column chromatography on silica gel (petroleum ether/ethyl acetate = 5:1) yielded **4l** (108.5 mg, 68%) as a pale yellow oil.

^{1}H NMR (400 MHz, $CDCl_3$) δ 7.69 (d, *J* = 7.4 Hz, 2H), 7.63 – 7.47 (m, 7H), 7.44 – 7.37 (m, 2H), 7.11 – 7.00 (m, 6H), 6.71 (s, 1H), 5.00 – 4.96 (m, 1H), 4.85 – 4.80 (m, 1H), 4.44 (d, *J* = 8.4 Hz, 1H), 3.05 (t, *J* = 12.0 Hz, 1H), 2.66 (d, *J* = 12.5 Hz, 1H), 2.48 (d, *J* = 9.4 Hz, 1H), 2.23 (s, 3H), 2.01 – 1.94 (m, 3H), 1.74 – 1.62 (m, 4H), 1.25 (s, 9H); ^{13}C NMR (100 MHz, $CDCl_3$) δ 152.9, 151.2, 151.0, 150.5, 147.5, 146.7, 144.3, 135.9, 131.7, 131.5, 130.8, 129.4, 129.2(3), 129.1(6), 128.6, 128.4, 128.3, 127.9, 127.7, 126.1, 125.3, 125.0, 124.4, 118.5, 57.1, 54.6, 49.6, 34.7, 31.1, 30.5, 30.4, 27.2, 24.3, 21.5; IR (neat): 2925, 1771, 1722, 1596, 1502, 1417, 1359, 1267, 1164, 1086, 961, 812, 690, 643, 545; HRMS (ESI) m/z: [M + Na]$^+$ calcd for $C_{44}H_{43}N_7NaO_6S$ 820.2888, found 820.2889.

(*Z*)-7-(3-fluorobenzylidene)-2,10-diphenyl-6-tosyl-4a,6,7,13,14,15,16,16a-octahydro-1*H*,5*H*,9*H*-[1,2,4]triazolo[1',2':1,2][1,2,4]triazepino[6,7-*c*][1,2,4]triazolo[1,2-*a*]cinnoline-1,3,9,11(2*H*,10*H*)-tetraone (4m)

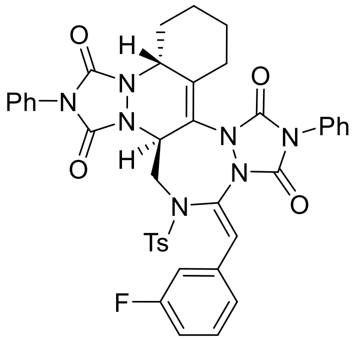

4m

The reaction was conducted with *N*-(3-cyclohexylideneallyl)-*N*-((3-fluorophenyl)ethynyl)-4-methylbenzenesulfonamide **1n** (0.2 mmol, 82.0 mg), 4-phenyl-3*H*-1,2,4-triazole-3,5(4*H*)-dione **2a** (0.8 mmol, 140.0 mg), 3Å molecular sieves (40 mg), and $Cu(OTf)_2$ (0.02 mmol, 7.2 mg) in dry DCE (4.0 mL) at 60 °C (60 °C, heating mantle temperature). Purification by column chromatography on silica gel (petroleum ether/ethyl acetate = 5:1) yielded **4m** (111.0 mg, 73%) as a pale yellow oil.

^{1}H NMR (400 MHz, $CDCl_3$) δ 7.64 – 7.57 (m, 5H), 7.54 – 7.48 (m, 4H), 7.45 – 7.38 (m, 2H), 7.09 – 7.03 (m, 1H), 7.00 (d, *J* = 8.0 Hz, 2H), 6.87 – 6.82 (m, 2H), 6.71 – 6.64 (m, 2H), 5.10 – 5.06 (m, 1H), 4.88 – 4.83 (m, 1H), 4.49 – 4.45 (m, 1H), 3.07 – 3.01 (m, 1H), 2.66 (d, *J* = 12.9 Hz, 1H), 2.50 (d, *J* = 9.7 Hz, 1H), 2.23 (s, 3H), 2.04 – 1.96 (m, 3H), 1.76 – 1.61 (m, 4H); ^{13}C NMR (100 MHz, $CDCl_3$) δ 162.3 (d, *J* = 245.0 Hz), 151.3, 150.9 (d, *J* = 16.0 Hz), 147.6, 146.9, 144.7, 135.6, 133.1 (d, *J* = 8.0 Hz), 131.4, 130.8, 129.8 (d, *J* = 8.0 Hz), 129.5, 129.5 (d, *J* = 3.0 Hz), 129.3, 129.2, 128.6, 128.3, 127.5, 127.1, 126.2, 125.0, 124.9 (d, *J* = 3.0 Hz), 118.7, 115.9 (d, *J* = 21.0 Hz), 114.6 (d, *J* = 22.0 Hz), 57.2, 54.6, 50.0, 30.6, 30.4, 27.2, 24.3, 21.3; IR (neat): 2929, 1773, 1719, 1654, 1502, 1420, 1358, 1274, 1164, 1087, 978, 881, 751, 617, 591, 548, 407; HRMS (ESI) m/z: $[M + H]^+$ calcd for $C_{40}H_{35}FN_7O_6S$ 760.2348, found 760.2348.

(*Z*)-7-(3-chlorobenzylidene)-2,10-diphenyl-6-tosyl-4a,6,7,13,14,15,16,16a-octahydro-1*H*,5*H*,9*H*-[1,2,4]triazolo[1',2':1,2][1,2,4]triazepino[6,7-*c*][1,2,4]triazolo[1,2-*a*]cinnoline-1,3,9,11(2*H*,10*H*)-tetraone (4n)

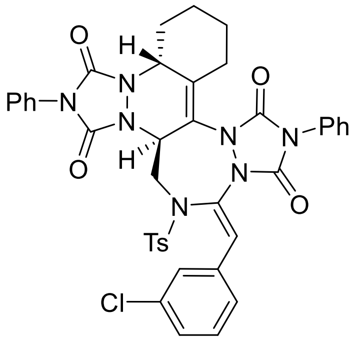

4n

The reaction was conducted with *N*-((3-chlorophenyl)ethynyl)-*N*-(3-cyclohexylideneallyl)-4-methylbenzenesulfonamide **1o** (0.2 mmol, 85.2 mg), 4-phenyl-3*H*-1,2,4-triazole-3,5(4*H*)-dione **2a** (0.8 mmol, 140.0 mg), 3Å molecular sieves (40 mg), and $Cu(OTf)_2$ (0.02 mmol, 7.2 mg) in dry DCE (4.0 mL) at 60 ^{o}C (60 °C, heating mantle temperature). Purification by column chromatography on silica gel (petroleum ether/ethyl acetate = 5:1) yielded **4n** (93.1 mg, 60%) as a pale yellow oil.

^{1}H NMR (400 MHz, $CDCl_3$) δ 7.63 – 7.58 (m, 5H), 7.54 – 7.48 (m, 4H), 7.44 – 7.38 (m, 2H), 7.10 (d, *J* = 7.8 Hz, 1H), 7.04 – 6.91 (m, 5H), 6.66 (s, 1H), 5.12 – 5.08 (m, 1H), 4.88 – 4.84 (m, 1H), 4.47 (d, *J* = 8.5 Hz, 1H), 3.04 (t, *J* = 12.0 Hz, 1H), 2.66 (d, *J* = 12.6 Hz, 1H), 2.50 (d, *J* = 10.2 Hz, 1H), 2.23 (s, 3H), 2.04 – 1.96 (m, 3H), 1.76 – 1.59 (m, 4H); ^{13}C NMR (100 MHz, $CDCl_3$) δ 151.3, 151.0, 150.9, 147.6, 146.9, 144.6, 135.5, 134.3, 132.8, 131.4, 130.8, 129.6, 129.5, 129.3, 129.2, 128.9, 128.6, 128.3, 127.6, 127.4, 127.2, 126.2, 125.0, 118.7, 57.2, 54.7, 50.0, 30.6, 30.4, 27.2, 24.3, 21.4; IR (neat): 2929, 1773, 1719, 1597, 1502, 1420, 1357, 1267, 1164, 1087, 965, 869, 765, 689, 616, 581, 504; HRMS (ESI) m/z: $[M + H]^+$ calcd for $C_{40}H_{35}ClN_7O_6S$ 776.2053, found 776.2054.

(*Z*)-7-(3-bromobenzylidene)-2,10-diphenyl-6-tosyl-4a,6,7,13,14,15,16,16a-octahydro-1*H*,5*H*,9*H*-[1,2,4]triazolo[1',2':1,2][1,2,4]triazepino[6,7-*c*][1,2,4]triazolo[1,2-*a*]cinnoline-1,3,9,11(2*H*,10*H*)-tetraone (4o)

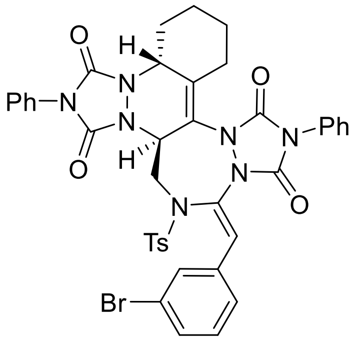

4o

The reaction was conducted with *N*-((3-bromophenyl)ethynyl)-*N*-(3-cyclohexylideneallyl)-4-methylbenzenesulfonamide **1p** (0.2 mmol, 94.0 mg), 4-phenyl-3*H*-1,2,4-triazole-3,5(4*H*)-dione **2a** (0.8 mmol, 140.0 mg), 3Å molecular sieves (40 mg), and $Cu(OTf)_2$ (0.02 mmol, 7.2 mg) in dry DCE (4.0 mL) at 60 °C (60 °C, heating mantle temperature). Purification by column chromatography on silica gel (petroleum ether/ethyl acetate = 5:1) yielded **4o** (118.2 mg, 72%) as a pale yellow oil.

^1H NMR (400 MHz, $CDCl_3$) δ 7.62 – 7.58 (m, 5H), 7.54 – 7.48 (m, 4H), 7.44 – 7.38 (m, 2H), 7.31 – 7.24 (m, 1H), 7.08 (s, 1H), 7.01 – 6.95 (m, 4H), 6.64 (s, 1H), 5.10 (d, *J* = 6.0 Hz, 1H), 4.87 – 4.83 (m, 1H), 4.47 (d, *J* = 8.3 Hz, 1H), 3.04 (t, *J* = 11.9 Hz, 1H), 2.66 (d, *J* = 12.5 Hz, 1H), 2.50 (d, *J* = 9.0 Hz, 1H), 2.23 (s, 3H), 2.04 – 1.96 (m, 3H), 1.76 – 1.63 (m, 4H); ^{13}C NMR (100 MHz, $CDCl_3$) δ 151.3, 150.9, 147.6, 147.0, 144.6, 135.4, 133.0, 131.8, 131.3, 130.8, 130.5, 129.7, 129.6, 129.3, 129.2, 129.1, 128.6, 128.3, 127.6, 127.4, 127.2, 126.2, 125.0, 122.6, 118.7, 57.2, 54.7, 50.0, 30.6, 30.4, 27.2, 24.3, 21.4; IR (neat): 2927, 1774, 1719, 1597, 1502, 1420, 1358, 1267, 1164, 1089, 965, 867, 736, 689, 645, 580, 504; HRMS (ESI) m/z: $[M + H]^+$ calcd for $C_{40}H_{35}BrN_7O_6S$ 820.1547, found 820.1547.

(*Z*)-7-(3-methylbenzylidene)-2,10-diphenyl-6-tosyl-4a,6,7,13,14,15,16,16a-octahydro-1*H*,5*H*,9*H*-[1,2,4]triazolo[1',2':1,2][1,2,4]triazepino[6,7-*c*][1,2,4]triazolo[1,2-*a*]cinnoline-1,3,9,11(2*H*,10*H*)-tetraone (4p)

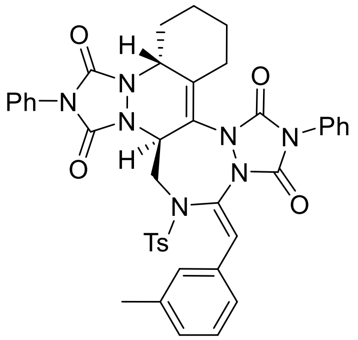

4p

The reaction was conducted with *N*-(3-cyclohexylideneallyl)-4-methyl-*N*-(*m*-tolylethynyl)benzenesulfonamide **1q** (0.2 mmol, 81.2 mg), 4-phenyl-3*H*-1,2,4-triazole-3,5(4*H*)-dione **2a** (0.8 mmol, 140.0 mg), 3Å molecular sieves (40 mg), and $Cu(OTf)_2$ (0.02 mmol, 7.2 mg) in dry DCE (4.0 mL) at 60 °C (60 °C, heating mantle temperature). Purification by column chromatography on silica gel (petroleum ether/ethyl acetate = 5:1) yielded **4p** (105.8 mg, 70%) as a pale yellow oil.

^{1}H NMR (400 MHz, $CDCl_3$) δ 7.67 – 7.57 (m, 5H), 7.54 – 7.48 (m, 4H), 7.44 – 7.37 (m, 2H), 7.00 – 6.83 (m, 6H), 6.70 (s, 1H), 5.06 (d, *J* = 6.4 Hz, 1H), 4.82 – 4.78 (m, 1H), 4.46 (d, *J* = 10.0 Hz, 1H), 3.04 (t, *J* = 11.9 Hz, 1H), 2.67 (d, *J* = 12.4 Hz, 1H), 2.49 (d, *J* = 10.3 Hz, 1H), 2.22 (s, 3H), 2.13 (s, 3H), 2.03 – 1.95 (m, 3H), 1.75 – 1.62 (m, 4H); ^{13}C NMR (100 MHz, $CDCl_3$) δ 151.2, 151.0, 150.8, 147.6, 146.8, 144.3, 137.9, 135.9, 131.4(3), 131.4(0), 130.8, 130.0, 129.4, 129.2(1), 129.1(6), 128.8, 128.4, 128.3, 127.4, 126.2, 126.1, 125.4, 125.0, 118.6, 57.1, 54.6, 49.8, 30.5, 30.4, 27.2, 24.3, 21.3, 21.2; IR (neat): 2926, 1771, 1716, 1598, 1503, 1417, 1361, 1276, 1163, 1088, 965, 810, 690, 646, 584, 545; HRMS (ESI) m/z: $[M + H]^+$ calcd for $C_{41}H_{38}N_7O_6S$ 756.2599, found 756.2596.

(*Z*)-7-(3,4-dichlorobenzylidene)-2,10-diphenyl-6-tosyl-4a,6,7,13,14,15,16,16a-octahydro-1*H*,5*H*,9*H*-[1,2,4]triazolo[1',2':1,2][1,2,4]triazepino[6,7-*c*][1,2,4]triazolo[1,2-*a*]cinnoline-1,3,9,11(2*H*,10*H*)-tetraone (4q)

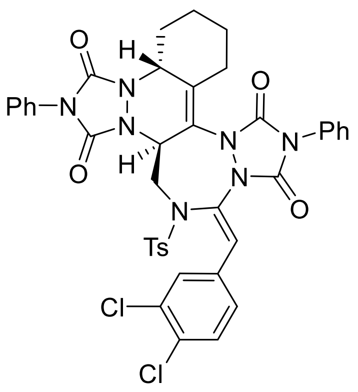

4q

The reaction was conducted with *N*-(3-cyclohexylideneallyl)-*N*-((3,4-dichlorophenyl)ethynyl)-4-methylbenzenesulfonamide **1r** (0.2 mmol, 92.0 mg), 4-phenyl-3*H*-1,2,4-triazole-3,5(4*H*)-dione **2a** (0.8 mmol, 140.0 mg), 3Å molecular sieves (40 mg), and $Cu(OTf)_2$ (0.02 mmol, 7.2 mg) in dry DCE (4.0 mL) at 60 ^oC (60 °C, heating mantle temperature). Purification by column chromatography on silica gel (petroleum ether/ethyl acetate = 5:1) yielded **4q** (111.9 mg, 69%) as a pale yellow oil.

^1H NMR (400 MHz, $CDCl_3$) δ 7.62 – 7.58 (m, 5H), 7.54 – 7.48 (m, 4H), 7.45 – 7.38 (m, 2H), 7.13 (d, *J* = 8.3 Hz, 1H), 7.05 – 6.99 (m, 3H), 6.87 (d, *J* = 8.3 Hz, 1H), 6.63 (s, 1H), 5.12 – 5.08 (m, 1H), 4.88 – 4.84 (m, 1H), 4.48 (d, *J* = 8.1 Hz, 1H), 3.04 (t, *J* = 11.9 Hz, 1H), 2.65 (d, *J* = 12.7 Hz, 1H), 2.51 (d, *J* = 9.8 Hz, 1H), 2.26 (s, 3H), 2.04 – 1.96 (m, 3H), 1.77 – 1.64 (m, 4H); ^{13}C NMR (100 MHz, $CDCl_3$) δ 151.3, 151.0, 147.6, 147.0, 145.0, 135.7, 133.0, 132.6, 131.3, 131.1, 130.8, 130.1, 129.5, 129.4, 129.3, 129.2, 128.6, 128.3, 128.0, 127.8, 127.8, 127.3, 126.2, 125.0, 118.7, 57.2, 54.7, 50.2, 30.6, 30.4, 27.2, 24.3, 21.4; IR (neat): 2929, 1773, 1719, 1598, 1503, 1414, 1357, 1274, 1164, 1089, 1029, 965, 876, 763, 692, 588, 543; HRMS (ESI) m/z: $[M + Na]^+$ calcd for $C_{40}H_{33}Cl_2N_7NaO_6S$ 832.1482, found 832.1481.

(*Z*)-7-(3,5-dimethylbenzylidene)-2,10-diphenyl-6-tosyl-4a,6,7,13,14,15,16,16a-octahydro-1*H*,5*H*,9*H*-[1,2,4]triazolo[1',2':1,2][1,2,4]triazepino[6,7-*c*][1,2,4]triazolo[1,2-*a*]cinnoline-1,3,9,11(2*H*,10*H*)-tetraone (4r)

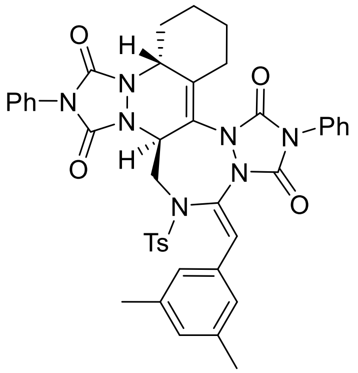

4r

The reaction was conducted with *N*-(3-cyclohexylideneallyl)-*N*-((3,5-dimethylphenyl)ethynyl)-4-methylbenzenesulfonamide **1s** (0.2 mmol, 84.0 mg), 4-phenyl-3*H*-1,2,4-triazole-3,5(4*H*)-dione **2a** (0.8 mmol, 140.0 mg), 3Å molecular sieves (40 mg), and $Cu(OTf)_2$ (0.02 mmol, 7.2 mg) in dry DCE (4.0 mL) at 60 °C (60 °C, heating mantle temperature). Purification by column chromatography on silica gel (petroleum ether/ethyl acetate = 5:1) yielded **4r** (106.3 mg, 69%) as a pale yellow oil.

^{1}H NMR (400 MHz, $CDCl_3$) δ 7.68 – 7.57 (m, 5H), 7.53 – 7.47 (m, 4H), 7.43 – 7.37 (m, 2H), 7.00 (d, *J* = 7.8 Hz, 2H), 6.79 (s, 1H), 6.66 (s, 3H), 5.11 – 5.07 (m, 1H), 4.79 – 4.75 (m, 1H), 4.45 (d, *J* = 8.1 Hz, 1H), 3.01 (t, *J* = 12.0 Hz, 1H), 2.68 (d, *J* = 12.7 Hz, 1H), 2.49 (d, *J* = 9.7 Hz, 1H), 2.23 (s, 3H), 2.10 (s, 6H), 2.03 – 1.95 (m, 3H), 1.75 – 1.62 (m, 4H); ^{13}C NMR (100 MHz, $CDCl_3$) δ 151.2, 151.0, 150.9, 147.6, 146.7, 144.2, 137.8, 136.1, 131.5, 131.0, 130.8, 130.7, 129.4, 129.2(2), 129.1(7), 128.4, 128.2, 127.4, 126.4, 126.2, 125.4, 125.0, 118.6, 57.1, 54.6, 49.8, 30.5, 30.4, 27.2, 24.3, 21.3, 21.1; IR (neat): 2925, 1771, 1716, 1596, 1503, 1417, 1361, 1273, 1163, 1088, 880, 812, 735, 689, 645, 545; HRMS (ESI) m/z: $[M + Na]^+$ calcd for $C_{42}H_{39}N_7NaO_6S$ 792.2575, found 792.2576.

(*Z*)-2,10-diphenyl-7-(thiophen-3-ylmethylene)-6-tosyl-4a,6,7,13,14,15,16,16a-octahydro-1*H*,5*H*,9*H*-[1,2,4]triazolo[1',2':1,2][1,2,4]triazepino[6,7-*c*][1,2,4]triazolo[1,2-*a*]cinnoline-1,3,9,11(2*H*,10*H*)-tetraone (4s)

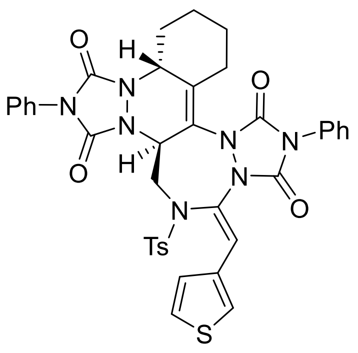

4s

The reaction was conducted with *N*-(3-cyclohexylideneallyl)-4-methyl-*N*-(thiophen-3-ylethynyl)benzenesulfonamide **1t** (0.2 mmol, 79.6 mg), 4-phenyl-3*H*-1,2,4-triazole-3,5(4*H*)-dione **2a** (0.8 mmol, 140.0 mg), 3Å molecular sieves (40 mg), and $Cu(OTf)_2$ (0.02 mmol, 7.2 mg) in dry DCE (4.0 mL) at 60 ^oC (60 °C, heating mantle temperature). Purification by column chromatography on silica gel (petroleum ether/ethyl acetate = 5:1) yielded **4s** (62.8 mg, 42%) as a pale yellow oil.

^1H NMR (400 MHz, $CDCl_3$) δ 7.74 (d, *J* = 8.1 Hz, 2H), 7.61 (d, *J* = 7.9 Hz, 2H), 7.56 – 7.47 (m, 5H), 7.42 – 7.39 (m, 2H), 7.24 (d, *J* = 1.5 Hz, 1H), 7.11 – 7.05 (m, 3H), 6.86 (d, *J* = 4.9 Hz, 1H), 6.81 (s, 1H), 4.90 – 4.87 (m, 1H), 4.80 – 4.76 (m, 1H), 4.45 – 4.41 (m, 1H), 3.05 – 2.99 (m, 1H), 2.63 (d, *J* = 12.4 Hz, 1H), 2.47 (d, *J* = 10.7 Hz, 1H), 2.30 (s, 3H), 2.00 – 1.94 (m, 3H), 1.72 – 1.61 (m, 4H); ^{13}C NMR (100 MHz, $CDCl_3$) δ 151.2, 151.0, 150.2, 147.4, 146.8, 144.8, 136.2, 132.3, 131.4, 130.8, 129.7, 129.3, 129.2, 128.5, 128.3, 127.7, 127.5, 126.8, 126.4, 126.0, 125.0, 123.9, 118.3, 57.2, 54.3, 49.2, 30.6, 30.4, 27.2, 24.3, 21.5; IR (neat): 2927, 1773, 1719, 1654, 1596, 1503, 1413, 1353, 1289, 1164, 1137, 1071, 965, 761, 578, 504; HRMS (ESI) m/z: $[M + Na]^+$ calcd for $C_{38}H_{33}N_7NaO_6S_2$ 770.1826, found 770.1827.

(*Z*)-7-(cyclopropylmethylene)-2,10-diphenyl-6-tosyl-4a,6,7,13,14,15,16,16a-octahydro-1*H*,5*H*,9*H*-[1,2,4]triazolo[1',2':1,2][1,2,4]triazepino[6,7-*c*][1,2,4]triazolo[1,2-*a*]cinnoline-1,3,9,11(2*H*,10*H*)-tetraone (4t)

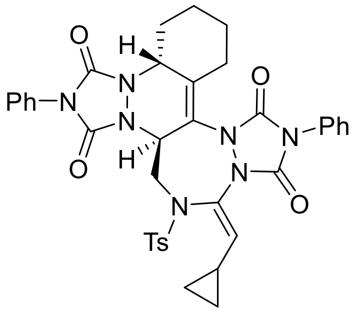

4t

The reaction was conducted with *N*-(3-cyclohexylideneallyl)-*N*-(cyclopropylethynyl)-4-methylbenzenesulfonamide **1u** (0.2 mmol, 71.0 mg), 4-phenyl-3*H*-1,2,4-triazole-3,5(4*H*)-dione **2a** (0.8 mmol, 140.0 mg), 3Å molecular sieves (40 mg), and $Cu(OTf)_2$ (0.02 mmol, 7.2 mg) in dry DCE (4.0 mL) at 60 ^{o}C (60 °C, heating mantle temperature). Purification by column chromatography on silica gel (petroleum ether/ethyl acetate = 5:1) yielded **4t** (98.8 mg, 70%) as a pale yellow oil.

^{1}H NMR (400 MHz, $CDCl_3$) δ 7.83 (d, *J* = 7.8 Hz, 2H), 7.57 – 7.48 (m, 7H), 7.42 – 7.38 (m, 2H), 7.30 – 7.25 (m, 3H), 5.35 – 5.31 (m, 1H), 4.87 – 4.75 (m, 2H), 4.50 – 4.47 (m, 1H), 2.99 – 2.94 (m, 1H), 2.64 (d, *J* = 12.2 Hz, 1H), 2.48 (d, *J* = 8.3 Hz, 1H), 2.39 (s, 3H), 2.00 – 1.94 (m, 3H), 1.74 – 1.61 (m, 3H), 0.96 – 0.88 (m, 1H), 0.79 – 0.69 (m, 1H), 0.45 – 0.31 (m, 3H); ^{13}C NMR (100 MHz, $CDCl_3$) δ 151.5, 150.9, 150.8, 147.3, 146.7, 144.3, 140.5, 136.3, 131.5, 130.9, 129.7, 129.2, 128.4, 128.0, 126.0, 125.4, 125.0, 124.6, 118.3, 57.2, 54.8, 50.0, 30.5, 30.4, 27.2, 24.4, 21.5, 9.6, 8.1, 7.5; IR (neat): 2924, 1769, 1716, 1661, 1569, 1502, 1417, 1296, 1163, 1090, 959, 871, 692, 613, 548; HRMS (ESI) m/z: $[M + Na]^+$ calcd for $C_{37}H_{35}N_7NaO_6S$ 728.2262, found 728.2261.

(*Z*)-7-benzylidene-2,10-diphenyl-6-tosyl-4a,5,6,7,13,14,15,15a-octahydro-1*H*,9*H*-cyclopenta[5,6][1,2,4]triazolo[1',2':1,2]pyridazino[3,4-*f*][1,2,4]triazolo[1,2-*a*][1,2,4]triazepine-1,3,9,11(2*H*,10*H*)-tetraone (4u)

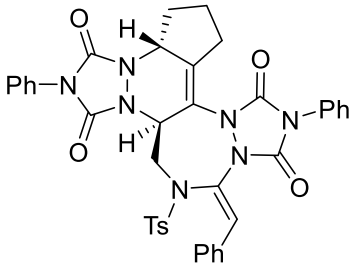

4u

The reaction was conducted with *N*-(3-cyclopentylideneallyl)-4-methyl-*N*-(phenylethynyl)benzenesulfonamide **1v** (0.2 mmol, 75.6 mg), 4-phenyl-3*H*-1,2,4-triazole-3,5(4*H*)-dione **2a** (0.8 mmol, 140.0 mg), 3Å molecular sieves (40 mg), and $Cu(OTf)_2$ (0.02 mmol, 7.2 mg) in dry DCE (4.0 mL) at 60 ºC (60 °C, heating mantle temperature). Purification by column chromatography on silica gel (petroleum ether/ethyl acetate = 5:1) yielded **4u** (101.9 mg, 70%) as a pale yellow oil.

^{1}H NMR (400 MHz, $CDCl_3$) δ 7.63 – 7.60 (m, 4H), 7.53 – 7.47 (m, 5H), 7.44 – 7.39 (m, 2H), 7.15 – 7.10 (m, 1H), 7.06 – 7.01 (m, 4H), 6.94 (d, *J* = 8.1 Hz, 2H), 6.73 (s, 1H), 5.59 – 5.54 (m, 1H), 4.93 – 4.90 (m, 1H), 4.25 – 4.21 (m, 1H), 3.25 – 3.19 (m, 1H), 2.88 – 2.73 (m, 2H), 2.55 – 2.48 (m, 1H), 2.21 (s, 3H), 2.12 – 2.00 (m, 2H), 1.86 – 1.78 (m, 1H), 1.32 – 1.24 (m, 1H); ^{13}C NMR (100 MHz, $CDCl_3$) δ 152.4, 151.8, 151.2, 150.2, 146.6, 144.3, 136.0, 131.4, 131.0, 130.7, 129.4, 129.3, 129.2(1), 129.1(9), 128.8, 128.6, 128.5, 128.4, 128.3, 127.4, 126.8, 126.3, 125.6, 120.3, 59.2, 56.9, 53.8, 31.6, 26.0, 21.8, 21.3; IR (neat): 2922, 2851, 1774, 1720, 1657, 1598, 1502, 1414, 1360, 1274, 1162, 690, 611; HRMS (ESI) m/z: $[M + Na]^+$ calcd for $C_{39}H_{33}N_7NaO_6S$ 750.2105, found 750.2108.

(*Z*)-7-benzylidene-2,10-bis(4-fluorophenyl)-6-tosyl-4a,6,7,13,14,15,16,16a-octahydro-1*H*,5*H*,9*H*-[1,2,4]triazolo[1',2':1,2][1,2,4]triazepino[6,7-*c*][1,2,4]triazolo[1,2-*a*]cinnoline-1,3,9,11(2*H*,10*H*)-tetraone (4v)

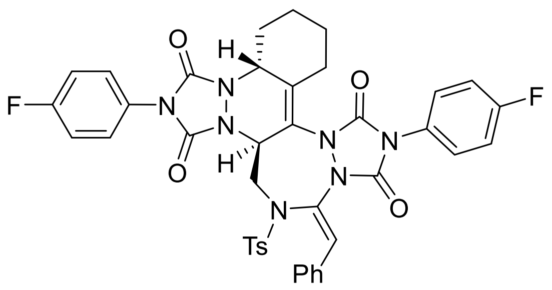

4v

The reaction was conducted with *N*-(3-cyclohexylideneallyl)-4-methyl-*N*-(phenylethynyl)benzenesulfonamide **1a** (0.2 mmol, 78.4 mg), 4-(4-fluorophenyl)-3*H*-1,2,4-triazole-3,5(4*H*)-dione **2b** (0.8 mmol, 154.4 mg), 3Å molecular sieves (40 mg), and $Cu(OTf)_2$ (0.02 mmol, 7.2 mg) in dry DCE (4.0 mL) at 60 ºC (60 °C, heating mantle temperature). Purification by column chromatography on silica gel (petroleum ether/ethyl acetate = 5:1) yielded **4v** (124.5 mg, 80%) as a pale yellow oil.

^{1}H NMR (400 MHz, $CDCl_3$) δ 7.64 – 7.56 (m, 5H), 7.23 – 7.16 (m, 5H), 7.03 – 6.96 (m, 6H), 6.72 – 6.69 (m, 1H), 5.07 – 5.05 (m, 1H), 4.82 (d, *J* = 13.3 Hz, 1H), 4.46 (d, *J* = 11.1 Hz, 1H), 3.04 (t, *J* = 10.8 Hz, 1H), 2.67 (d, *J* = 11.2 Hz, 1H), 2.49 (d, *J* = 9.8 Hz, 1H), 2.22 (s, 3H), 2.04 – 1.97 (m, 3H), 1.78 – 1.63 (m, 4H); ^{13}C NMR (100 MHz, $CDCl_3$) δ 162.2 (d, *J* = 247.0 Hz), 161.9 (d, *J* = 247.0 Hz), 151.1, 151.0, 150.8, 147.5, 147.0, 144.6, 135.6, 131.4, 130.8, 129.5, 129.2, 128.6, 128.3, 128.2 (d, *J* = 9.0 Hz), 127.5, 127.3 (d, *J* = 3.0 Hz), 126.9 (d, *J* = 9.0 Hz), 126.8 (d, *J* = 3.0 Hz), 125.6, 118.4, 116.3 (d, *J* = 23.0 Hz), 116.2 (d, *J* = 23.0 Hz), 57.2, 54.7, 49.9, 30.5(3), 30.4(6), 27.2, 24.3, 21.4; IR (neat): 2929, 1773, 1719, 1654, 1618, 1512, 1420, 1355, 1276, 1163, 1082, 959, 866, 749, 604, 467; HRMS (ESI) m/z: $[M + Na]^+$ calcd for $C_{40}H_{33}F_2N_7NaO_6S$ 800.2073, found 800.2070.

(*Z*)-7-benzylidene-2,10-bis(4-chlorophenyl)-6-tosyl-4a,6,7,13,14,15,16,16a-octahydro-1*H*,5*H*,9*H*-[1,2,4]triazolo[1',2':1,2][1,2,4]triazepino[6,7-*c*][1,2,4]triazolo[1,2-*a*]cinnoline-1,3,9,11(2*H*,10*H*)-tetraone (4w)

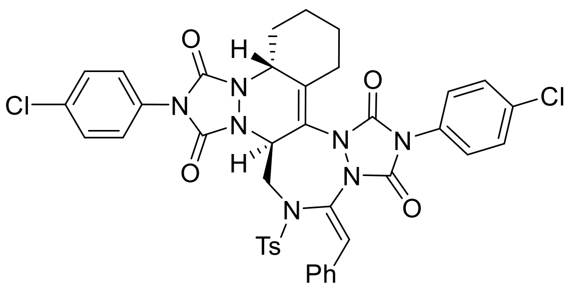

4w

The reaction was conducted with *N*-(3-cyclohexylideneallyl)-4-methyl-*N*-(phenylethynyl)benzenesulfonamide **1a** (0.2 mmol, 78.4 mg), 4-(4-chlorophenyl)-3*H*-1,2,4-triazole-3,5(4*H*)-dione **2c** (0.8 mmol, 168.0 mg), 3Å molecular sieves (40 mg), and $Cu(OTf)_2$ (0.02 mmol, 7.2 mg) in dry DCE (4.0 mL) at 60 ºC (60 °C, heating mantle temperature). Purification by column chromatography on silica gel (petroleum ether/ethyl acetate = 5:1) yielded **4w** (116.8 mg, 72%) as a pale yellow oil.

^{1}H NMR (400 MHz, $CDCl_3$) δ 7.63 – 7.56 (m, 5H), 7.50 – 7.45 (m, 4H), 7.15 (t, *J* = 5.6 Hz, 1H), 7.06 – 7.03 (m, 4H), 6.96 (d, *J* = 7.6 Hz, 2H), 6.76 – 6.71 (m, 1H), 5.07 – 5.03 (m, 1H), 4.84 – 4.79 (m, 1H), 4.46 (d, *J* = 7.9 Hz, 1H), 3.03 (t, *J* = 12.1 Hz, 1H), 2.66 (d, *J* = 12.7 Hz, 1H), 2.49 (d, *J* = 9.7 Hz, 1H), 2.21 (s, 3H), 2.04 – 1.96 (m, 3H), 1.74 – 1.64 (m, 4H); ^{13}C NMR (100 MHz, $CDCl_3$) δ 150.9, 150.7, 150.6, 147.3, 147.0, 144.6, 135.6, 134.3, 133.9, 131.5, 130.7, 130.0, 129.5, 129.4, 129.3, 129.2, 128.6, 128.3, 127.5, 127.3, 126.4, 126.0, 125.5, 118.3, 57.2, 54.6, 49.9, 30.5, 30.5, 27.2, 24.3, 21.4; IR (neat): 2925, 1774, 1719, 1596, 1496, 1417, 1361, 1277, 1164, 1092, 1018, 963, 833, 751, 653, 578, 508; HRMS (ESI) m/z: $[M + Na]^+$ calcd for $C_{40}H_{33}Cl_2N_7NaO_6S$ 832.1482, found 832.1483.

(*Z*)-7-benzylidene-6-tosyl-2,10-bis(4-(trifluoromethyl)phenyl)-4a,6,7,13,14,15,16,16a-octahydro-1*H*,5*H*,9*H*-[1,2,4]triazolo[1',2':1,2][1,2,4]triazepino[6,7-*c*][1,2,4]triazolo[1,2-*a*]cinnoline-1,3,9,11(2*H*,10*H*)-tetraone (4x)

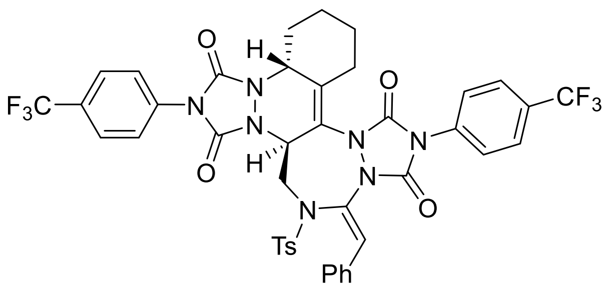

4x

The reaction was conducted with *N*-(3-cyclohexylideneallyl)-4-methyl-*N*-(phenylethynyl)benzenesulfonamide **1a** (0.2 mmol, 78.4 mg), 4-(4-(trifluoromethyl)phenyl)-3*H*-1,2,4-triazole-3,5(4*H*)-dione **2d** (0.8 mmol, 194.4 mg), 3Å molecular sieves (40 mg), and $Cu(OTf)_2$ (0.02 mmol, 7.2 mg) in dry DCE (4.0 mL) at 60 ^{o}C (60 °C, heating mantle temperature). Purification by column chromatography on silica gel (petroleum ether/ethyl acetate = 5:1) yielded **4x** (122.9 mg, 70%) as a pale yellow oil. ^{1}H NMR (400 MHz, $CDCl_3$) δ 7.83 – 7.75 (m, 7H), 7.62 (d, *J* = 7.4 Hz, 2H), 7.47 – 7.39 (m, 1H), 7.16 (t, *J* = 6.1 Hz, 1H), 7.06 – 7.01 (m, 3H), 6.97 (d, *J* = 7.8 Hz, 2H), 6.73 (s, 1H), 5.12 – 5.08 (m, 1H), 4.85 – 4.80 (m, 1H), 4.51 – 4.47 (m, 1H), 3.08 – 3.02 (m, 1H), 2.68 (d, *J* = 12.9 Hz, 1H), 2.51 (d, *J* = 10.4 Hz, 1H), 2.21 (s, 3H), 2.06 – 1.98 (m, 3H), 1.76 – 1.63 (m, 4H); ^{13}C NMR (100 MHz, $CDCl_3$) δ 150.6, 150.5, 150.4, 147.3, 147.0, 144.8, 135.4, 134.6, 134.1, 131.8, 130.6, 130.2, 129.9, 129.5, 129.4, 128.6, 128.4, 127.5, 126.4 (q, *J* = 3.5 Hz), 126.2 (q, *J* = 4.4 Hz), 126.0, 125.4, 124.6, 118.8, 118.1, 57.2, 54.7, 49.9, 30.6, 30.5, 27.2, 24.3, 21.4; IR (neat): 2929, 1776, 1728, 1612, 1524, 1417, 1325, 1165, 1127, 1067, 1016, 959, 843, 760, 653, 611, 577, 512; HRMS (ESI) m/z: $[M + Na]^+$ calcd for $C_{42}H_{33}F_6N_7NaO_6S$ 900.2009, found 900.2012.

(*Z*)-7-benzylidene-2,10-di-*p*-tolyl-6-tosyl-4a,6,7,13,14,15,16,16a-octahydro-1*H*,5*H*,9*H*-[1,2,4]triazolo[1',2':1,2][1,2,4]triazepino[6,7-*c*][1,2,4]triazolo[1,2-*a*]cinnoline-1,3,9,11(2*H*,10*H*)-tetraone (4y)

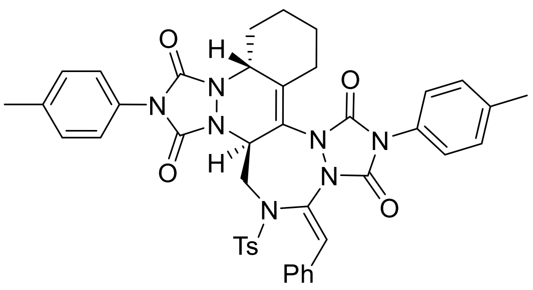

4y

The reaction was conducted with *N*-(3-cyclohexylideneallyl)-4-methyl-*N*-(phenylethynyl)benzenesulfonamide **1a** (0.2 mmol, 78.4 mg), 4-(*p*-tolyl)-3*H*-1,2,4-triazole-3,5(4*H*)-dione **2e** (0.8 mmol, 151.2 mg), 3Å molecular sieves (40 mg), and $Cu(OTf)_2$ (0.02 mmol, 7.2 mg) in dry DCE (4.0 mL) at 60 ºC (60 °C, heating mantle temperature). Purification by column chromatography on silica gel (petroleum ether/ethyl acetate = 5:1) yielded **4y** (120.1 mg, 78%) as a pale yellow oil.

^{1}H NMR (400 MHz, $CDCl_3$) δ 7.64 (d, *J* = 7.7 Hz, 2H), 7.49 – 7.41 (m, 4H), 7.33 – 7.28 (m, 4H), 7.16 – 7.07 (m, 4H), 6.97 (d, *J* = 7.8 Hz, 2H), 6.72 (s, 1H), 5.01 – 4.97 (m, 1H), 4.84 – 4.80 (m, 1H), 4.44 (d, *J* = 7.9 Hz, 1H), 3.05 (t, *J* = 12.0 Hz, 1H), 2.64 (d, *J* = 12.8 Hz, 1H), 2.47 (d, *J* = 10.0 Hz, 1H), 2.40 (s, 3H), 2.39 (s, 3H), 2.22 (s, 3H), 2.01 – 1.93 (m, 3H), 1.73 – 1.64 (m, 4H); ^{13}C NMR (100 MHz, $CDCl_3$) δ 151.4, 151.1, 150.7, 147.7, 146.7, 144.5, 138.6, 138.4, 135.8, 131.3, 131.0, 129.9, 129.7, 129.5, 129.1, 128.7, 128.6, 128.3, 128.1, 127.5, 126.0, 125.5, 124.9, 118.6, 57.1, 54.5, 49.8, 30.5, 30.4, 27.1, 24.3, 21.4, 21.2, 21.1; IR (neat): 2927, 1769, 1715, 1646, 1516, 1417, 1359, 1273, 1164, 1084, 961, 863, 736, 655, 610, 511; HRMS (ESI) m/z: $[M + Na]^+$ calcd for $C_{42}H_{39}N_7NaO_6S$ 792.2575, found 792.2578.

(*Z*)-7-benzylidene-6-tosyl-2,10-bis(4-(trifluoromethoxy)phenyl)-4a,6,7,13,14,15,16,16a-octahydro-1*H*,5*H*,9*H*-[1,2,4]triazolo[1',2':1,2][1,2,4]triazepino[6,7-*c*][1,2,4]triazolo[1,2-*a*]cinnoline-1,3,9,11(2*H*,10*H*)-tetraone (4z)

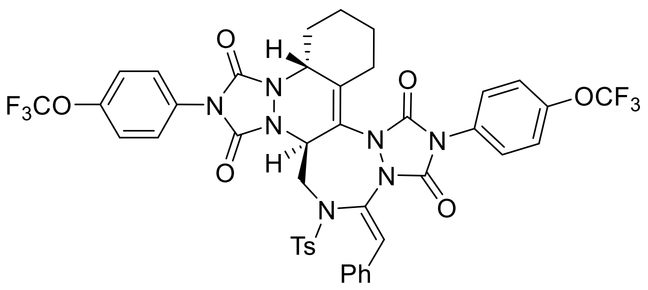

4z

The reaction was conducted with *N*-(3-cyclohexylideneallyl)-4-methyl-*N*-(phenylethynyl)benzenesulfonamide **1a** (0.2 mmol, 78.4 mg), 4-(4-(trifluoromethoxy)phenyl)-3*H*-1,2,4-triazole-3,5(4*H*)-dione **2f** (0.8 mmol, 207.2 mg), 3Å molecular sieves (40 mg), and $Cu(OTf)_2$ (0.02 mmol, 7.2 mg) in dry DCE (4.0 mL) at 60 ^{o}C (60 °C, heating mantle temperature). Purification by column chromatography on silica gel (petroleum ether/ethyl acetate = 5:1) yielded **4z** (123.8 mg, 68%) as a pale yellow oil. ^{1}H NMR (400 MHz, $CDCl_3$) δ 7.71 – 7.67 (m, 4H), 7.62 (d, *J* = 7.2 Hz, 2H), 7.38 – 7.34 (m, 4H), 7.15 (t, *J* = 6.2 Hz, 1H), 7.06 – 7.00 (m, 3H), 6.96 (d, *J* = 7.5 Hz, 2H), 6.72 (s, 1H), 5.10 – 5.07 (m, 1H), 4.85 – 4.80 (m, 1H), 4.48 (d, *J* = 8.6 Hz, 1H), 3.04 (t, *J* = 11.9 Hz, 1H), 2.67 (d, *J* = 12.6 Hz, 1H), 2.50 (d, *J* = 9.7 Hz, 1H), 2.21 (s, 3H), 2.05 – 1.97 (m, 3H), 1.76 – 1.67 (m, 4H); ^{13}C NMR (100 MHz, $CDCl_3$) δ 150.8(0), 150.7(5), 150.6, 148.7 (q, *J* = 3.5 Hz), 148.3 (q, *J* = 1.7 Hz) 147.3, 147.1, 144.6, 135.5, 131.5, 130.7, 129.9, 129.5, 129.4, 129.2, 128.5, 128.3, 127.6, 127.5, 126.2, 125.5, 121.8, 121.7, 119.1, 118.3, 57.2, 54.7, 49.9, 30.5(4), 30.4(7), 27.2, 24.3, 21.3; IR (neat): 2935, 1776, 1729, 1645, 1514, 1417, 1361, 1259, 1222, 1164, 1084, 1016, 961, 849, 654, 516; HRMS (ESI) m/z: $[M + Na]^+$ calcd for $C_{42}H_{33}F_6N_7NaO_8S$ 932.1908, found 932.1907.

(*Z*)-7-benzylidene-2,10-bis(3-chlorophenyl)-6-tosyl-4a,6,7,13,14,15,16,16a-octahydro-1*H*,5*H*,9*H*-[1,2,4]triazolo[1',2':1,2][1,2,4]triazepino[6,7-*c*][1,2,4]triazolo[1,2-*a*]cinnoline-1,3,9,11(2*H*,10*H*)-tetraone (4aa)

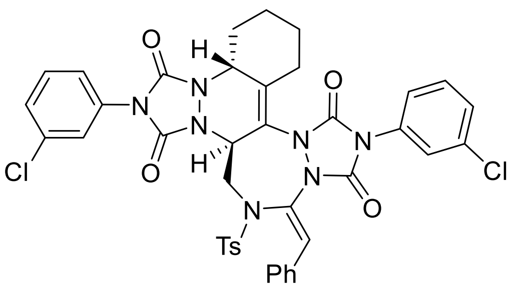

4aa

The reaction was conducted with *N*-(3-cyclohexylideneallyl)-4-methyl-*N*-(phenylethynyl)benzenesulfonamide **1a** (0.2 mmol, 78.4 mg), 4-(3-chlorophenyl)-3*H*-1,2,4-triazole-3,5(4*H*)-dione **2g** (0.8 mmol, 168.0 mg), 3Å molecular sieves (40 mg), and $Cu(OTf)_2$ (0.02 mmol, 7.2 mg) in dry DCE (4.0 mL) at 60 °C (60 °C, heating mantle temperature). Purification by column chromatography on silica gel (petroleum ether/ethyl acetate = 5:1) yielded **4aa** (111.9 mg, 69%) as a pale yellow oil.

^{1}H NMR (400 MHz, $CDCl_3$) δ 7.67 – 7.63 (m, 4H), 7.58 – 7.53 (m, 2H), 7.48 – 7.37 (m, 4H), 7.21 – 7.15 (m, 1H), 7.11 – 7.05 (m, 3H), 6.98 (d, *J* = 6.9 Hz, 2H), 6.72 (s, 1H), 5.06 (d, *J* = 3.1 Hz, 1H), 4.83 (d, *J* = 13.3 Hz, 1H), 4.47 (d, *J* = 11.0 Hz, 1H), 3.04 (t, *J* = 12.0 Hz, 1H), 2.66 (d, *J* = 12.4 Hz, 1H), 2.50 (d, *J* = 10.2 Hz, 1H), 2.22 (s, 3H), 2.04 – 1.97 (m, 3H), 1.74 – 1.64 (m, 4H); ^{13}C NMR (100 MHz, $CDCl_3$) δ 150.7, 150.5, 150.4, 147.1, 144.6, 135.6, 134.8, 132.5, 132.0, 131.6, 130.7, 130.2, 130.1, 129.5, 129.2, 128.7, 128.6, 128.5, 128.4, 127.5, 126.2, 125.4, 124.9, 124.1, 122.8, 118.3, 57.2, 54.6, 49.9, 30.5(4), 30.4(7), 27.2, 24.3, 21.4; IR (neat): 2924, 1771, 1715, 1643, 1592, 1486, 1406, 1359, 1259, 1161, 1086, 973, 812, 655, 545; HRMS (ESI) m/z: $[M + Na]^+$ calcd for $C_{40}H_{33}Cl_2N_7NaO_6S$ 832.1482, found 832.1482.

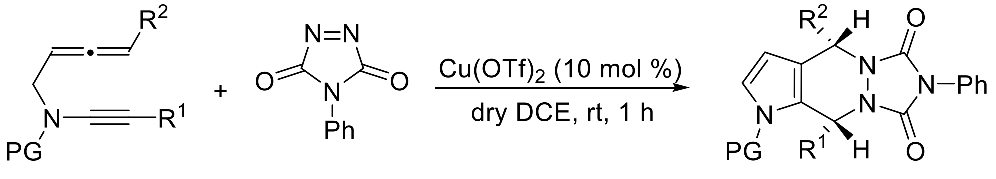

Supplementary Figure 96. Synthesis of tricyclic pyrrolidines **6**.

General procedure for the synthesis of tricyclic pyrrolidines 6:

PTAD (*cis*-diazenes) **2a** (0.3 mmol, 52.5 mg), and $Cu(OTf)_2$ (0.02 mmol, 7.2 mg) were added in this order to the allenynes **5** (0.2 mmol) in dry DCE (8.0 mL) at room temperature. Under N_2 atmosphere, the reaction mixture was stirred at room temperature and the progress of the reaction was monitored by TLC. The reaction typically took 1 h. Upon completion, the mixture was then concentrated and the residue was purified by chromatography on silica gel (eluent: petroleum ether/ethyl acetate) to afford the desired products **6**.

4-ethyl-7,10-diphenyl-1-tosyl-4,10-dihydro-1*H*,6*H*-pyrrolo[2,3-*d*][1,2,4]triazolo[1,2-*a*]pyridazine-6,8(7*H*)-dione (6a)

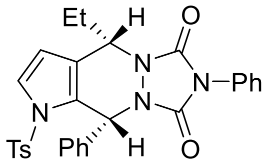

6a

The reaction was conducted with *N*-(hexa-2,3-dien-1-yl)-4-methyl-*N*-(phenylethynyl)benzenesulfonamide **5a** (0.2 mmol, 70.2 mg), 4-phenyl-3*H*-1,2,4-triazole-3,5(4*H*)-dione **2a** (0.3 mmol, 52.5 mg), and $Cu(OTf)_2$ (0.02 mmol, 7.2 mg) in dry DCE (4.0 mL) at room temperature under N_2 atmosphere. Purification by column chromatography on silica gel (petroleum ether/ethyl acetate = 3:1) yielded **6a** (61.1 mg, 58%) as a pale yellow oil.

^{1}H NMR (400 MHz, $CDCl_3$) ^{1}H NMR (400 MHz, $CDCl_3$) δ 7.39 – 7.35 (m, 5H), 7.32 – 7.24 (m, 4H), 7.19 – 7.15 (m, 2H), 7.11 (d, *J* = 8.3 Hz, 2H), 6.99 (d, *J* = 8.2 Hz, 2H), 6.74 (s, 1H), 6.32 (d, *J* = 3.4 Hz, 1H), 4.93 – 4.87 (m, 1H), 2.76 – 2.69 (m, 1H), 2.33 (s, 3H), 2.25 – 2.16 (m, 1H), 1.04 (t, *J* = 7.4 Hz, 3H); ^{13}C NMR (100 MHz, $CDCl_3$) δ 153.9, 150.3, 144.8, 135.9, 135.0, 131.0, 129.7, 129.4, 128.9, 128.6, 128.3, 128.0, 126.9, 125.4, 125.1, 123.3(1), 123.2(7), 108.8, 58.1, 55.8, 26.0, 21.5, 8.6; IR (neat): 2921, 2850,1771, 1716, 1503, 1456, 1416, 1374, 1239, 1173, 1129, 718, 673, 541; HRMS (ESI) m/z: [M + H]$^+$ calcd for $C_{29}H_{26}N_4NaO_4S$ 549.1567, found 549.1566.

4-ethyl-1-((4-methoxyphenyl)sulfonyl)-7,10-diphenyl-4,10-dihydro-1*H*,6*H*-pyrrolo[2,3-*d*][1,2,4]triazolo[1,2-*a*]pyridazine-6,8(7*H*)-dione (6b)

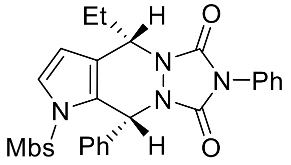

6b

The reaction was conducted with *N*-(hexa-2,3-dien-1-yl)-4-methoxy-*N*-(phenylethynyl)benzenesulfonamide **5b** (0.2 mmol, 73.4 mg), 4-phenyl-3*H*-1,2,4-triazole-3,5(4*H*)-dione **2a** (0.3 mmol, 52.5 mg), and $Cu(OTf)_2$ (0.02 mmol, 7.2 mg) in dry DCE (8.0 mL) at room temperature under N_2 atmosphere. Purification by column chromatography on silica gel (petroleum ether/ethyl acetate = 3:1) yielded **6b** (73.8 mg, 68%) as a pale yellow oil.

^{1}H NMR (400 MHz, $CDCl_3$) δ 7.40 – 7.26 (m, 8H), 7.21 – 7.14 (m, 5H), 6.75 (s, 1H), 6.64 (d, *J* = 7.3 Hz, 2H), 6.32 (d, *J* = 1.7 Hz, 1H), 4.94 – 4.87 (m, 1H), 3.80 (s, 3H), 2.76 – 2.69 (m, 1H), 2.24 – 2.18 (m, 1H), 1.04 (t, *J* = 6.9 Hz, 3H); ^{13}C NMR (100 MHz, $CDCl_3$) δ 163.5, 153.8, 150.2, 136.0, 131.0, 129.4, 129.2(4), 129.1(6), 128.9, 128.7, 128.3, 128.0, 125.4, 124.9, 123.1(4), 123.0(8), 114.3, 108.7, 58.1, 55.7, 55.6, 26.0, 8.6; IR (neat): 2918, 1771, 1716, 1593, 1498, 1457, 1414, 1373, 1265, 1167, 1089, 1025, 835, 805, 587; HRMS (ESI) m/z: $[M + Na]^+$ calcd for $C_{29}H_{26}N_4NaO_5S$ 565.1516, found 565.1518.

4-ethyl-7,10-diphenyl-1-(phenylsulfonyl)-4,10-dihydro-1*H*,6*H*-pyrrolo[2,3-*d*][1,2,4]triazolo[1,2-*a*]pyridazine-6,8(7*H*)-dione (6c)

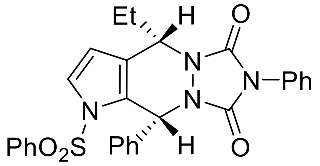

6c

The reaction was conducted with *N*-(hexa-2,3-dien-1-yl)-*N*-(phenylethynyl)benzenesulfonamide **5c** (0.2 mmol, 67.4 mg), 4-phenyl-3*H*-1,2,4-triazole-3,5(4*H*)-dione **2a** (0.3 mmol, 52.5 mg), and $Cu(OTf)_2$ (0.02 mmol, 7.2 mg) in dry DCE (8.0 mL) at room temperature under N_2 atmosphere. Purification by column chromatography on silica gel (petroleum ether/ethyl acetate = 3:1) yielded **6c** (57.5 mg, 56%) as a pale yellow oil.

^{1}H NMR (400 MHz, $CDCl_3$) ^{1}H NMR (400 MHz, $CDCl_3$) δ 7.46 – 7.43 (m, 1H), 7.39 – 7.35 (m, 5H), 7.33 – 7.30 (m, 1H), 7.26 – 7.21 (m, 7H), 7.17 – 7.13 (m, 2H), 6.74 (s, 1H), 6.35 (d, *J* = 3.5 Hz, 1H), 4.94 – 4.88 (m, 1H), 2.78 – 2.71 (m, 1H), 2.25 – 2.18 (m, 1H), 1.04 (t, *J* = 7.4 Hz, 3H); ^{13}C NMR (100 MHz, $CDCl_3$) δ 153.9, 150.3, 137.9, 135.7, 133.5, 131.0, 129.5, 129.1, 129.0, 128.8, 128.3, 128.0, 126.7, 125.4, 125.2, 123.4, 123.4, 109.0, 58.1, 55.8, 25.9, 8.5; IR (neat): 2933, 1771, 1716, 1503, 1448, 1413, 1375, 1282, 1237, 1140, 1089, 1042, 856, 726, 602; HRMS (ESI) m/z: $[M + Na]^+$ calcd for $C_{28}H_{24}N_4NaO_4S$ 535.1410, found 535.1411.

1-((4-bromophenyl)sulfonyl)-4-ethyl-7,10-diphenyl-4,10-dihydro-1*H*,6*H*-pyrrolo[2,3-*d*][1,2,4]triazolo[1,2-*a*]pyridazine-6,8(7*H*)-dione (6d)

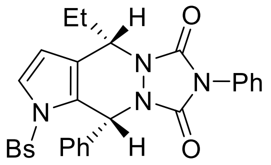

6d

The reaction was conducted with 4-bromo-*N*-(hexa-2,3-dien-1-yl)-*N*-(phenylethynyl)benzenesulfonamide **5d** (0.2 mmol, 83.2 mg), 4-phenyl-3*H*-1,2,4-triazole-3,5(4*H*)-dione **2a** (0.3 mmol, 52.5 mg), and $Cu(OTf)_2$ (0.02 mmol, 7.2 mg) in dry DCE (8.0 mL) at room temperature under N_2 atmosphere. Purification by column chromatography on silica gel (petroleum ether/ethyl acetate = 3:1) yielded **6d** (65.0 mg, 55%) as a pale yellow oil.

^{1}H NMR (400 MHz, $CDCl_3$) δ 7.41 – 7.35 (m, 5H), 7.32 – 7.26 (m, 4H), 7.23 – 7.15 (m, 4H), 7.00 (d, *J* = 8.6 Hz, 2H), 6.71 (s, 1H), 6.36 (d, *J* = 3.4 Hz, 1H), 4.94 – 4.88 (m, 1H), 2.80 – 2.73 (m, 1H), 2.26 – 2.18 (m, 1H), 1.05 (t, *J* = 7.4 Hz, 3H); ^{13}C NMR (100 MHz,

$CDCl_3$) δ 153.9, 150.3, 136.8, 135.5, 132.3, 131.0, 129.6, 129.0, 128.9, 128.8, 128.4, 128.2, 128.1, 125.4, 125.2, 123.8, 123.3, 109.2, 58.1, 55.7, 25.8, 8.6; IR (neat): 2931, 1771, 1716, 1574, 1503, 1456, 1415, 1378, 1280, 1176, 1129, 1087, 1009, 744, 614; HRMS (ESI) m/z: $[M + Na]^+$ calcd for $C_{28}H_{23}BrN_4NaO_4S$ 613.0516, found 613.0518.

4-ethyl-1-((4-nitrophenyl)sulfonyl)-7,10-diphenyl-4,10-dihydro-1*H*,6*H*-pyrrolo[2,3-*d*][1,2,4]triazolo[1,2-*a*]pyridazine-6,8(7*H*)-dione (6e)

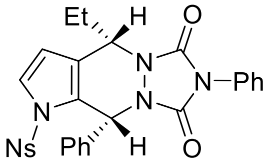

6e

The reaction was conducted with *N*-(hexa-2,3-dien-1-yl)-4-nitro-*N*-(phenylethynyl)benzenesulfonamide **5e** (0.2 mmol, 76.4 mg), 4-phenyl-3*H*-1,2,4-triazole-3,5(4*H*)-dione **2a** (0.3 mmol, 52.5 mg), and $Cu(OTf)_2$ (0.02 mmol, 7.2 mg) in dry DCE (8.0 mL) at room temperature under N_2 atmosphere. Purification by column chromatography on silica gel (petroleum ether/ethyl acetate = 3:1) yielded **6e** (58.0 mg, 52%) as a pale yellow oil.

^{1}H NMR (400 MHz, $CDCl_3$) δ 7.97 (d, J = 8.7 Hz, 2H), 7.42 – 7.26 (m, 9H), 7.20 (d, J = 7.6 Hz, 2H), 7.15 – 7.11 (m, 2H), 6.70 (s, 1H), 6.42 (d, J = 3.4 Hz, 1H), 4.95 – 4.89 (m, 1H), 2.83 – 2.76 (m, 1H), 2.26 – 2.20 (m, 1H), 1.04 (t, J = 7.4 Hz, 3H); ^{13}C NMR (100 MHz, $CDCl_3$) δ 153.8, 150.4, 150.1, 143.1, 135.2, 130.8, 129.7, 129.1, 129.0, 128.5, 128.1(3), 128.0(7), 125.6, 125.4, 124.4, 124.1, 123.5, 109.7, 58.1, 55.7, 25.6, 8.5; IR (neat): 2850, 1772, 1716, 1532, 1503, 1416, 1382, 1348, 1182, 1127, 855, 740, 615, 587, 460; HRMS (ESI) m/z: $[M + Na]^+$ calcd for $C_{28}H_{23}N_5NaO_6S$ 580.1261, found 580.1264.

4-ethyl-10-(4-fluorophenyl)-1-((4-methoxyphenyl)sulfonyl)-7-phenyl-4,10-dihydro-1*H*,6*H*-pyrrolo[2,3-*d*][1,2,4]triazolo[1,2-*a*]pyridazine-6,8(7*H*)-dione (6f)

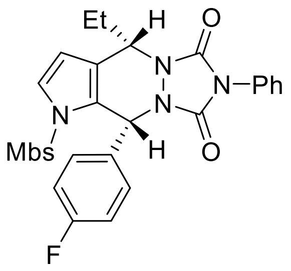

6f

The reaction was conducted with *N*-((4-fluorophenyl)ethynyl)-*N*-(hexa-2,3-dien-1-yl)-4-methoxybenzenesulfonamide **5f** (0.2 mmol, 77.0 mg), 4-phenyl-3*H*-1,2,4-triazole-3,5(4*H*)-dione **2a** (0.3 mmol, 52.5 mg), and $Cu(OTf)_2$ (0.02 mmol, 7.2 mg) in dry DCE (8.0 mL) at room temperature under N_2 atmosphere. Purification by column chromatography on silica gel (petroleum ether/ethyl acetate = 3:1) yielded **6f** (75.2 mg, 67%) as a pale yellow oil.

^{1}H NMR (400 MHz, $CDCl_3$) δ 7.35 – 7.29 (m, 5H), 7.25 (t, *J* = 7.1 Hz, 1H), 7.15 – 7.11 (m, 4H), 6.79 – 6.74 (m, 2H), 6.66 – 6.61 (m, 3H), 6.25 (d, *J* = 3.4 Hz, 1H), 4.87 – 4.80 (m, 1H), 3.74 (s, 3H), 2.71 – 2.65 (m, 1H), 2.16 – 2.09 (m, 1H), 0.95 (t, *J* = 7.4 Hz, 3H); ^{13}C NMR (100 MHz, $CDCl_3$) δ 163.6, 162.8 (d, *J* = 246.0 Hz), 153.8, 150.4, 131.8 (d, *J* = 3.0 Hz), 131.2 (d, *J* = 9.0 Hz), 130.9, 129.2, 129.0, 128.1, 125.4, 124.7, 123.5, 123.3, 115.2 (d, *J* = 22.0 Hz), 114.2, 108.5, 58.1, 55.7, 55.0, 25.8, 8.5; IR (neat): 2637, 1867, 1771, 1716, 1500, 1456, 1415, 1373, 1265, 1192, 1168, 1089, 1025, 677, 588; HRMS (ESI) m/z: $[M + Na]^+$ calcd for $C_{29}H_{25}FN_4NaO_5S$ 583.1422, found 583.1425.

10-(4-chlorophenyl)-4-ethyl-1-((4-methoxyphenyl)sulfonyl)-7-phenyl-4,10-dihydro-1*H*,6*H*-pyrrolo[2,3-*d*][1,2,4]triazolo[1,2-*a*]pyridazine-6,8(7*H*)-dione (6g)

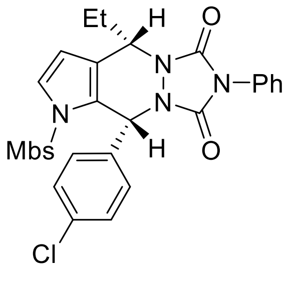

6g

The reaction was conducted with *N*-((4-chlorophenyl)ethynyl)-*N*-(hexa-2,3-dien-1-yl)-4-methoxybenzenesulfonamide **5g** (0.2 mmol, 80.4 mg), 4-phenyl-3*H*-1,2,4-triazole-3,5(4*H*)-dione **2a** (0.3 mmol, 52.5 mg), and $Cu(OTf)_2$ (0.02 mmol, 7.2 mg) in dry DCE (8.0 mL) at room temperature under N_2 atmosphere. Purification by column chromatography on silica gel (petroleum ether/ethyl acetate = 3:1) yielded **6g** (90.0 mg, 78%) as a pale yellow oil.

^{1}H NMR (400 MHz, $CDCl_3$) δ 7.42 – 7.36 (m, 5H), 7.33 – 7.30 (m, 1H), 7.17 – 7.09 (m, 6H), 6.69 – 6.67 (m, 3H), 6.33 (d, *J* = 3.4 Hz, 1H), 4.93 – 4.87 (m, 1H), 3.83 (s, 3H), 2.79 – 2.72 (m, 1H), 2.22 – 2.16 (m, 1H), 1.01 (t, *J* = 7.4 Hz, 3H); ^{13}C NMR (100 MHz, $CDCl_3$) δ 163.6, 153.8, 150.4, 134.7, 134.3, 130.9, 130.8, 129.1, 129.0, 128.9, 128.4, 128.1, 125.4, 124.4, 123.6, 123.4, 114.2, 108.4, 58.1, 55.7, 55.0, 25.7, 8.4; IR (neat): 2849, 1770, 1716, 1593, 1499, 1457, 1416, 1372, 1314, 1265, 1192, 1167, 1017, 774, 677; HRMS (ESI) m/z: $[M + Na]^+$ calcd for $C_{29}H_{25}ClN_4NaO_5S$ 599.1126, found 599.1128.

10-(4-bromophenyl)-4-ethyl-1-((4-methoxyphenyl)sulfonyl)-7-phenyl-4,10-dihydro-1*H*,6*H*-pyrrolo[2,3-*d*][1,2,4]triazolo[1,2-*a*]pyridazine-6,8(7*H*)-dione (6h)

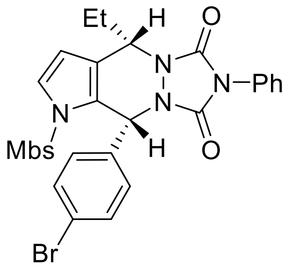

6h

The reaction was conducted with *N*-((4-bromophenyl)ethynyl)-*N*-(hexa-2,3-dien-1-yl)-4-methoxybenzenesulfonamide **5h** (0.2 mmol, 89.2 mg), 4-phenyl-3*H*-1,2,4-triazole-3,5(4*H*)-dione **2a** (0.3 mmol, 52.5 mg), and $Cu(OTf)_2$ (0.02 mmol, 7.2 mg) in dry DCE (8.0 mL) at room temperature under N_2 atmosphere. Purification by column chromatography on silica gel (petroleum ether/ethyl acetate = 3:1) yielded **6h** (87.1 mg, 70%) as a pale yellow oil.

^{1}H NMR (400 MHz, $CDCl_3$) δ 7.43 – 7.36 (m, 5H), 7.34 – 7.30 (m, 1H), 7.26 – 7.25 (m, 2H), 7.15 (d, *J* = 8.9 Hz, 2H), 7.08 (d, *J* = 8.2 Hz, 2H), 6.70 – 6.64 (m, 3H), 6.33 (d, *J* =

3.3 Hz, 1H), 4.93 – 4.87 (m, 1H), 3.85 (s, 3H), 2.79 – 2.72 (m, 1H), 2.22 – 2.15 (m, 1H), 1.00 (t, *J* = 7.3 Hz, 3H); ^{13}C NMR (100 MHz, $CDCl_3$) δ 163.6, 153.8, 150.4, 134.8, 131.4, 131.2, 130.9, 129.1, 129.0, 128.9, 128.1, 125.4, 124.3, 123.6, 123.4, 123.0, 114.2, 108.4, 58.1, 55.8, 55.0, 25.7, 8.4; IR (neat): 2926, 1714, 1679, 1658, 1645, 1634, 1594, 1498, 1416, 1167, 1129, 804, 676, 586; HRMS (ESI) m/z: $[M + Na]^+$ calcd for $C_{29}H_{25}BrN_4NaO_5S$ 643.0621, found 643.0620.

10-(3-chlorophenyl)-4-ethyl-1-((4-methoxyphenyl)sulfonyl)-7-phenyl-4,10-dihydro-1*H*,6*H*-pyrrolo[2,3-*d*][1,2,4]triazolo[1,2-*a*]pyridazine-6,8(7*H*)-dione (6i)

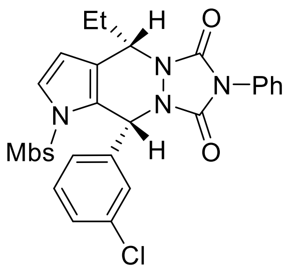

6i

The reaction was conducted with *N*-((3-chlorophenyl)ethynyl)-*N*-(hexa-2,3-dien-1-yl)-4-methoxybenzenesulfonamide **5i** (0.2 mmol, 80.4 mg), 4-phenyl-3*H*-1,2,4-triazole-3,5(4*H*)-dione **2a** (0.3 mmol, 52.5 mg), and $Cu(OTf)_2$ (0.02 mmol, 7.2 mg) in dry DCE (4.0 mL) at room temperature under N_2 atmosphere. Purification by column chromatography on silica gel (petroleum ether/ethyl acetate = 3:1) yielded **6i** (83.1 mg, 72%) as a pale yellow oil.

^{1}H NMR (400 MHz, $CDCl_3$) δ 7.35 – 7.30 (m, 6H), 7.26 – 7.23 (m, 1H), 7.15 – 7.10 (m, 4H), 6.82 (s, 1H), 6.63 – 6.59 (m, 3H), 6.26 (d, *J* = 3.2 Hz, 1H), 4.87 – 4.80 (m, 1H), 3.75 (s, 3H), 2.73 – 2.66 (m, 1H), 2.14 – 2.09 (m, 1H), 0.95 (t, *J* = 7.3 Hz, 3H); ^{13}C NMR (100 MHz, $CDCl_3$) δ 163.7, 153.9, 150.2, 137.7, 134.2, 130.9, 129.7, 129.0(0), 128.9(6), 128.9(1), 128.8(9), 128.4, 128.1, 125.4, 124.0, 123.6, 114.3, 108.6, 58.2, 55.7, 54.9, 25.8, 8.3; IR (neat): 2919, 2849, 2194, 1772, 1716, 1594, 1499, 1458, 1416, 1373, 1192, 1168, 1131, 1089, 1025; HRMS (ESI) m/z: $[M + Na]^+$ calcd for $C_{29}H_{25}ClN_4NaO_5S$ 599.1126, found 599.1126.

10-(3-bromophenyl)-4-ethyl-1-((4-methoxyphenyl)sulfonyl)-7-phenyl-4,10-dihydro-1*H*,6*H*-pyrrolo[2,3-*d*][1,2,4]triazolo[1,2-*a*]pyridazine-6,8(7*H*)-dione (6j)

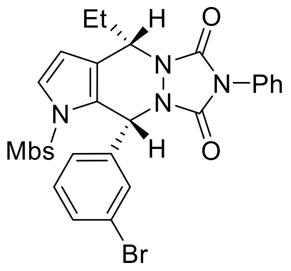

6j

The reaction was conducted with *N*-((3-bromophenyl)ethynyl)-*N*-(hexa-2,3-dien-1-yl)-4-methoxybenzenesulfonamide **5a** (0.2 mmol, 89.2 mg), 4-phenyl-3*H*-1,2,4-triazole-3,5(4*H*)-dione **2a** (0.3 mmol, 52.5 mg), and $Cu(OTf)_2$ (0.02 mmol, 7.2 mg) in dry DCE (8.0 mL) at room temperature under N_2 atmosphere. Purification by column chromatography on silica gel (petroleum ether/ethyl acetate = 3:1) yielded **6j** (93.3 mg, 75%) as a pale yellow oil.

^{1}H NMR (400 MHz, $CDCl_3$) δ 7.48 (d, *J* = 7.7 Hz, 1H), 7.43 – 7.37 (m, 6H), 7.33 – 7.30 (m, 1H), 7.18 – 7.14 (m, 3H), 7.02 (s, 1H), 6.69 – 6.65 (m, 3H), 6.33 (d, *J* = 3.4 Hz, 1H), 4.91 – 4.88 (m, 1H), 3.83 (s, 3H), 2.82 – 2.75 (m, 1H), 2.21 – 2.16 (m, 1H), 1.02 (t, *J* = 7.4 Hz, 3H); ^{13}C NMR (100 MHz, $CDCl_3$) δ 163.7, 153.9, 150.2, 137.9, 131.9, 131.7, 130.9, 130.1, 128.9(9), 128.9(7), 128.9(2), 128.8(8), 128.1, 125.4, 124.0, 123.6(0), 123.5(7), 122.4, 114.3, 108.5, 58.2, 55.7, 54.9, 25.7, 8.3; IR (neat): 2922, 2851, 1772, 1716, 1594, 1499, 1416, 1373, 1265, 1192, 1167, 1153, 1089; HRMS (ESI) m/z: [M + Na]$^+$ calcd for $C_{29}H_{25}BrN_4NaO_5S$ 643.0621, found 643.0621.

4-ethyl-1-((4-methoxyphenyl)sulfonyl)-7-phenyl-10-(p-tolyl)-4,10-dihydro-1*H*,6*H*-pyrrolo[2,3-*d*][1,2,4]triazolo[1,2-*a*]pyridazine-6,8(7*H*)-dione (6k)

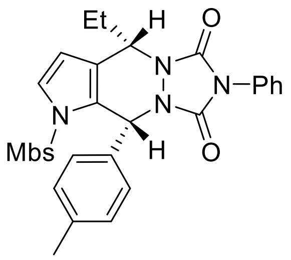

6k

The reaction was conducted with *N*-(hexa-2,3-dien-1-yl)-4-methoxy-*N*-(*p*-tolylethynyl)benzenesulfonamide **5a** (0.2 mmol, 76.2 mg), 4-phenyl-3*H*-1,2,4-triazole-3,5(4*H*)-dione **2a** (0.3 mmol, 52.5 mg), and $Cu(OTf)_2$ (0.02 mmol, 7.2 mg) in dry DCE (4.0 mL) at room temperature under N_2 atmosphere. Purification by column chromatography on silica gel (petroleum ether/ethyl acetate = 3:1) yielded **6k** (73.5 mg, 66%) as a pale yellow oil.

^{1}H NMR (400 MHz, $CDCl_3$) δ 7.34 – 7.31 (m, 4H), 7.28 (d, *J* = 3.0 Hz, 1H), 7.25 – 7.22 (m, 1H), 7.10 (d, *J* = 8.6 Hz, 2H), 7.04 (d, *J* = 7.6 Hz, 2H), 6.89 (d, *J* = 7.8 Hz, 2H), 6.63 (s, 1H), 6.56 (d, *J* = 8.7 Hz, 2H), 6.23 (d, *J* = 3.1 Hz, 1H), 4.85 – 4.78 (m, 1H), 3.73 (s, 3H), 2.68 – 2.61 (m, 1H), 2.25 (s, 3H), 2.15 – 2.09 (m, 1H), 0.95 (t, *J* = 7.3 Hz, 3H); ^{13}C NMR (100 MHz, $CDCl_3$) δ 163.5, 153.8, 150.3, 138.4, 133.2, 131.1, 129.3(9), 129.3(5), 129.2, 128.9(4), 128.9(0), 127.9, 125.4, 125.1, 123.2, 123.0, 114.1, 108.6, 58.1, 55.6, 55.4, 26.0, 21.2, 8.5; IR (neat): 2923, 2852, 1770, 1714, 1594, 1498, 1457, 1412, 1373, 1263, 1166, 1140, 1090; HRMS (ESI) m/z: $[M + Na]^+$ calcd for $C_{30}H_{28}N_4NaO_5S$ 579.1673, found 579.1678.

10-(4-(*tert*-butyl)phenyl)-4-ethyl-1-((4-methoxyphenyl)sulfonyl)-7-phenyl-4,10-dihydro-1*H*,6*H*-pyrrolo[2,3-*d*][1,2,4]triazolo[1,2-*a*]pyridazine-6,8(7*H*)-dione (6l)

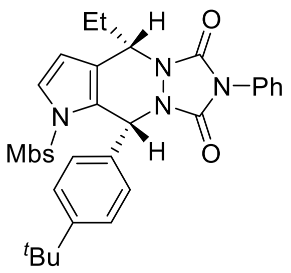

6l

The reaction was conducted with *N*-((4-(*tert*-butyl)phenyl)ethynyl)-*N*-(hexa-2,3-dien-1-yl)-4-methoxybenzenesulfonamide **5a** (0.2 mmol, 84.8 mg), 4-phenyl-3*H*-1,2,4-triazole-3,5(4*H*)-dione **2a** (0.3 mmol, 52.5 mg), and $Cu(OTf)_2$ (0.02 mmol, 7.2 mg) in dry DCE (8.0 mL) at room temperature under N_2 atmosphere. Purification by column chromatography on silica gel (petroleum ether/ethyl acetate = 3:1) yielded **6l** (73.1 mg, 61%) as a pale yellow oil.

^{1}H NMR (400 MHz, $CDCl_3$) δ 7.33 – 7.31 (m, 4H), 7.24 (d, *J* = 3.5 Hz, 2H), 7.17 – 7.12 (m, 4H), 7.06 (d, *J* = 9.0 Hz, 2H), 6.67 (s, 1H), 6.57 (d, *J* = 9.0 Hz, 2H), 6.23 (d, *J* = 3.4 Hz, 1H), 4.84 – 4.82 (m, 1H), 3.71 (s, 3H), 2.68 – 2.61 (m, 1H), 2.17 – 2.12 (m, 1H), 1.24 (s, 9H), 0.99 (t, *J* = 7.4 Hz, 1H); ^{13}C NMR (100 MHz, $CDCl_3$) δ 163.5, 153.9, 151.6, 150.2, 133.0, 131.1, 129.5, 129.3, 129.1, 129.0, 128.9, 127.9, 125.4, 125.2, 123.0, 122.8, 114.2, 108.8, 58.1, 55.6, 55.5, 34.6, 31.4, 26.1, 8.7; IR (neat): 2921, 2850, 1769, 1716, 1645, 1595, 1499, 1413, 1375, 1167, 1131, 1090, 1025; HRMS (ESI) m/z: $[M + Na]^+$ calcd for $C_{33}H_{34}N_4NaO_5S$ 621.2142, found 621.2146.

10-(3,5-dimethylphenyl)-4-ethyl-1-((4-methoxyphenyl)sulfonyl)-7-phenyl-4,10-dihydro-1*H*,6*H*-pyrrolo[2,3-*d*][1,2,4]triazolo[1,2-*a*]pyridazine-6,8(7*H*)-dione (6m)

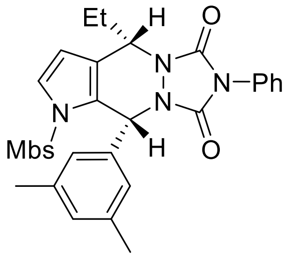

6m

The reaction was conducted with *N*-((3,5-dimethylphenyl)ethynyl)-*N*-(hexa-2,3-dien-1-yl)-4-methoxybenzenesulfonamide **5m** (0.2 mmol, 79.2 mg), 4-phenyl-3*H*-1,2,4-triazole-3,5(4*H*)-dione **2a** (0.3 mmol, 52.5 mg), and $Cu(OTf)_2$ (0.02 mmol, 7.2 mg) in dry DCE (8.0 mL) at room temperature under N_2 atmosphere. Purification by column chromatography on silica gel (petroleum ether/ethyl acetate = 3:1) yielded **6m** (74.2 mg, 65%) as a pale yellow oil.

^{1}H NMR (400 MHz, $CDCl_3$) δ 7.43 – 7.40 (m, 4H), 7.36 (d, *J* = 3.4 Hz, 1H), 7.33 – 7.30 (m, 1H), 7.14 (d, *J* = 9.0 Hz, 2H), 6.87 (s, 1H), 6.83 – 6.78 (m, 2H), 6.69 (s, 1H), 6.62 (d, *J* = 9.0 Hz, 2H), 6.31 (d, *J* = 3.4 Hz, 1H), 4.91 – 4.86 (m, 1H), 3.80 (s, 3H), 2.78 – 2.71 (m, 1H), 2.23 – 2.17 (m, 1H), 2.14 (s, 6H), 1.02 (t, *J* = 7.4 Hz, 3H); ^{13}C NMR (100 MHz, $CDCl_3$) δ 163.4, 154.1, 150.0, 137.8, 135.8, 131.2, 130.4, 129.3, 129.2, 129.0, 128.0, 127.4, 125.4, 125.1, 123.1, 122.9, 114.0, 108.5, 58.2, 55.6, 55.4, 26.1, 21.2, 8.4; IR (neat): 2921, 2850, 1770, 1715, 1647, 1595, 1499, 1416, 1373, 1264, 1161, 1131, 1090, 1026; HRMS (ESI) m/z: $[M + H]^+$ calcd for $C_{31}H_{31}N_4O_5S$ 571.2010, found 571.2014.

1-((4-bromophenyl)sulfonyl)-4-isopropyl-7,10-diphenyl-4,10-dihydro-1*H*,6*H*-pyrrolo[2,3-*d*][1,2,4]triazolo[1,2-*a*]pyridazine-6,8(7*H*)-dione (6n)

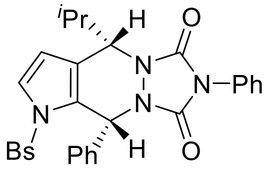

6n

The reaction was conducted with 4-bromo-*N*-(5-methylhexa-2,3-dien-1-yl)-*N*-(phenylethynyl)benzenesulfonamide **5n** (0.2 mmol, 86.0 mg), 4-phenyl-3*H*-1,2,4-triazole-3,5(4*H*)-dione **2a** (0.3 mmol, 52.5 mg), and $Cu(OTf)_2$ (0.02 mmol, 7.2 mg) in dry DCE (8.0 mL) at room temperature under N_2 atmosphere. Purification by column chromatography on silica gel (petroleum ether/ethyl acetate = 3:1) yielded **6n** (65.4 mg, 54%) as a yellow solid (mp 137-139 °C).

^{1}H NMR (400 MHz, $CDCl_3$) δ 7.45 – 7.42 (m, 4H), 7.37 – 7.33 (m, 4H), 7.29 (d, *J* = 7.3 Hz, 1H), 7.23 (d, *J* = 7.5 Hz, 2H), 7.18 (d, *J* = 7.4 Hz, 2H), 7.15 – 7.12 (m, 2H), 6.80 (s, 1H), 6.42 (d, *J* = 3.4 Hz, 1H), 4.84 (d, *J* = 5.5 Hz, 1H), 2.89 – 2.82 (m, 1H), 1.18 (d, *J* = 7.1 Hz, 3H), 0.94 (d, *J* = 6.8 Hz, 3H); ^{13}C NMR (100 MHz, $CDCl_3$) δ 152.3, 150.5, 136.8, 136.7, 132.5, 131.2, 129.3, 129.1, 129.0, 128.7, 128.4, 128.3, 128.0, 125.5, 125.3, 122.7, 111.0, 61.7, 55.5, 32.1, 20.2, 17.8; IR (neat): 2964, 1770, 1716,1574, 1503, 1416, 1392, 1177, 1141, 1087, 1069, 1009, 744, 614; HRMS (ESI) m/z: $[M + Na]^+$ calcd for $C_{29}H_{25}BrN_4NaO_4S$ 627.0672, found 627.0674.

4-isopropyl-1-((4-methoxyphenyl)sulfonyl)-7,10-diphenyl-4,10-dihydro-1*H*,6*H*-pyrrolo[2,3-*d*][1,2,4]triazolo[1,2-*a*]pyridazine-6,8(7*H*)-dione (6o)

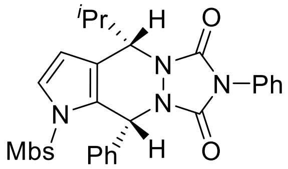

6o

The reaction was conducted with 4-methoxy-*N*-(5-methylhexa-2,3-dien-1-yl)-*N*-(phenylethynyl)benzenesulfonamide **5o** (0.2 mmol, 76.2 mg), 4-phenyl-3*H*-1,2,4-triazole-3,5(4*H*)-dione **2a** (0.3 mmol, 52.5 mg), and $Cu(OTf)_2$ (0.02 mmol, 7.2 mg) in dry DCE (8.0 mL) at room temperature under N_2 atmosphere. Purification by column chromatography on silica gel (petroleum ether/ethyl acetate = 3:1) yielded **6o** (66.8 mg, 60%) as a pale yellow oil.

^{1}H NMR (400 MHz, $CDCl_3$) δ 7.47 – 7.41 (m, 5H), 7.35 – 7.28 (m, 6H), 7.21 (d, *J* = 6.8 Hz, 2H), 6.86 (s, 1H), 6.72 (d, *J* = 7.6 Hz, 2H), 6.37 (d, *J* = 3.3 Hz, 1H), 4.82 (d, *J* = 5.7 Hz, 1H), 3.81 (s, 3H), 2.79 – 2.72 (m, 1H), 1.15 (d, *J* = 7.0 Hz, 3H), 0.90 (d, *J* = 6.8 Hz, 3H); ^{13}C NMR (100 MHz, $CDCl_3$) δ 163.7, 152.3, 150.3, 137.4, 131.3, 129.3, 129.2, 129.1, 129.0, 128.5, 128.3, 128.0, 125.5, 125.0, 122.6, 122.1, 114.4, 110.6, 61.7, 55.7, 55.2, 32.3, 20.2, 17.8; IR (neat): 2928, 1769, 1715, 1594, 1498, 1416, 1373, 1264, 1194, 1166, 1090, 1026, 589; HRMS (ESI) m/z: $[M + Na]^+$ calcd for $C_{30}H_{28}N_4NaO_5S$ 579.1673, found 579.1677.

1-((4-methoxyphenyl)sulfonyl)-7,10-diphenyl-4-propyl-4,10-dihydro-1*H*,6*H*-pyrrolo[2,3-*d*][1,2,4]triazolo[1,2-*a*]pyridazine-6,8(7*H*)-dione (6p)

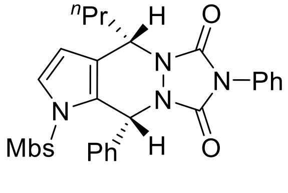

6p

The reaction was conducted with *N*-(hepta-2,3-dien-1-yl)-4-methoxy-*N*-(phenylethynyl)benzenesulfonamide **5p** (0.2 mmol, 76.2 mg), 4-phenyl-3*H*-1,2,4-triazole-3,5(4*H*)-dione **2a** (0.3 mmol, 52.5 mg), and $Cu(OTf)_2$ (0.02 mmol, 7.2 mg) in dry DCE (8.0 mL) at room temperature under N_2 atmosphere. Purification by column chromatography on silica gel (petroleum ether/ethyl acetate = 3:1) yielded **6p** (74.6 mg, 67%) as a pale yellow oil.

^{1}H NMR (400 MHz, $CDCl_3$) δ 7.41 – 7.38 (m, 4H), 7.33 – 7.25 (m, 5H), 7.22 – 7.19 (m, 2H), 7.16 – 7.13 (m, 2H), 6.75 (s, 1H), 6.65 – 6.63 (m, 2H), 6.32 (s, 1H), 4.94 – 4.88 (m, 1H), 3.80 (s, 3H), 2.60 – 2.53 (m, 1H), 2.17 (t, *J* = 12.9 Hz, 1H), 1.67 – 1.62 (m, 1H), 1.47 – 1.39 (m, 1H), 0.97 (t, *J* = 6.2 Hz, 3H); ^{13}C NMR (100 MHz, $CDCl_3$) δ 163.5, 153.7, 150.3, 136.1, 131.0, 129.5, 129.3, 129.2, 128.9, 128.7, 128.4, 128.0, 125.4, 124.6, 123.5, 123.1, 114.3, 108.8, 57.3, 55.7, 55.6, 35.4, 17.6, 14.2; IR (neat): 2931, 1771, 1716, 1594, 1499, 1416, 1373, 1265, 1193, 1167, 1132, 1090, 1027; HRMS (ESI) m/z: [M + Na]$^+$ calcd for $C_{30}H_{28}N_4NaO_5S$ 579.1673, found 579.1673.

4-decyl-1-((4-methoxyphenyl)sulfonyl)-7,10-diphenyl-4,10-dihydro-1*H*,6*H*-pyrrolo[2,3-*d*][1,2,4]triazolo[1,2-*a*]pyridazine-6,8(7*H*)-dione (6q)

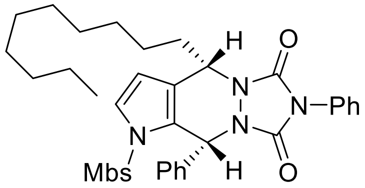

6q

The reaction was conducted with 4-methoxy-*N*-(phenylethynyl)-*N*-(tetradeca-2,3-dien-1-yl)benzenesulfonamide **5q** (0.2 mmol, 96.0 mg), 4-phenyl-3*H*-1,2,4-triazole-3,5(4*H*)-dione **2a** (0.3 mmol, 52.5 mg), and $Cu(OTf)_2$ (0.02 mmol, 7.2 mg) in dry DCE (8.0 mL) at room temperature under N_2 atmosphere. Purification by column chromatography on silica gel (petroleum ether/ethyl acetate = 3:1) yielded **6q** (83.8 mg, 64%) as a pale yellow oil.

^{1}H NMR (400 MHz, $CDCl_3$) δ 7.41 – 7.38 (m, 4H), 7.33 – 7.25 (m, 5H), 7.22 – 7.17 (m, 4H), 6.75 (s, 1H), 6.65 (d, *J* = 9.0 Hz, 2H), 6.31 (d, *J* = 3.4 Hz, 1H), 4.94 – 4.88 (m, 1H),

3.80 (s, 3H), 2.60 – 2.51 (m, 1H), 2.18 (t, *J* = 12.6 Hz, 1H), 1.33 – 1.24 (m, 16H), 0.87 (t, *J* = 6.8 Hz, 3H); ^{13}C NMR (100 MHz, $CDCl_3$) δ 163.6, 153.6, 150.2, 136.2, 131.1, 129.4, 129.3, 129.2, 128.9, 128.7, 128.3, 128.0, 125.4, 124.6, 123.6, 123.1, 114.3, 108.8, 57.4, 55.6, 33.2, 31.9, 29.6(0), 29.5(6), 29.4(5), 29.3, 24.3, 22.7, 14.1; IR (neat): 2923, 2852, 1717, 1651, 1545, 1499, 1456, 1419, 1375, 1314, 1264, 1095, 616, 588; HRMS (ESI) m/z: $[M + Na]^+$ calcd for $C_{37}H_{42}N_4NaO_5S$ 677.2768, found 677.2767.

4-(dec-9-en-1-yl)-1-((4-methoxyphenyl)sulfonyl)-7,10-diphenyl-4,10-dihydro-1*H*,6*H*-pyrrolo[2,3-*d*][1,2,4]triazolo[1,2-*a*]pyridazine-6,8(7*H*)-dione (6r)

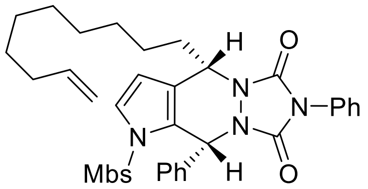

6r

The reaction was conducted with *4*-methoxy-*N*-(phenylethynyl)-*N*-(tetradeca-2,3,13-trien-1-yl)benzenesulfonamide **5r** (0.2 mmol, 95.6 mg), 4-phenyl-3*H*-1,2,4-triazole-3,5(4*H*)-dione **2a** (0.3 mmol, 52.5 mg), and $Cu(OTf)_2$ (0.02 mmol, 7.2 mg) in dry DCE (8.0 mL) at room temperature under N_2 atmosphere. Purification by column chromatography on silica gel (petroleum ether/ethyl acetate = 3:1) yielded **6r** (82.3 mg, 63%) as a pale yellow oil.

^{1}H NMR (400 MHz, $CDCl_3$) δ 7.42 – 7.37 (m, 4H), 7.33 – 7.25 (m, 5H), 7.22 – 7.17 (m, 4H), 6.75 (s, 1H), 6.65 (d, *J* = 7.5 Hz, 2H), 6.31 (d, *J* = 3.3 Hz, 1H), 5.85 – 5.75 (m, 1H), 5.01 – 4.91 (m, 3H), 3.80 (s, 3H), 2.59 – 2.51 (m, 1H), 2.18 (t, *J* = 12.9 Hz, 1H), 2.05 – 2.00 (m, 2H), 1.36 – 1.26 (m, 12H); ^{13}C NMR (100 MHz, $CDCl_3$) δ 163.6, 153.7, 150.2, 139.2, 136.2, 131.1, 129.4, 129.3, 129.2, 128.9, 128.7, 128.3, 128.0, 125.4, 124.7, 123.6, 123.1, 114.3, 114.1, 108.8, 57.4, 55.7, 33.8, 33.2, 29.6, 29.4, 29.1, 28.9, 24.3; IR (neat): 2920, 2850, 1868, 1771, 1748, 1716, 1688, 1646, 1543, 1456, 1417, 1096, 1054, 1029; HRMS (ESI) m/z: $[M + Na]^+$ calcd for $C_{37}H_{40}N_4NaO_5S$ 675.2612, found 675.2615.

Synthetic Applications

(*Z*)-*N*-((1,3-dioxo-2-phenyl-6-styryl-2,3,5,7,8,9,10,10a-octahydro-1*H*-[1,2,4]triazolo[1,2-*a*]cinnolin-5-yl)methyl)-4-methylbenzenesulfonamide (7)

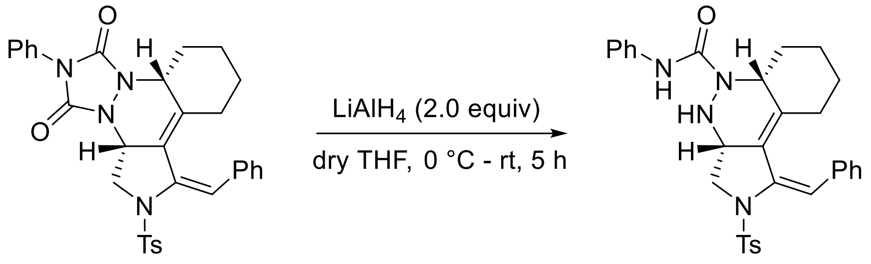

Supplementary Figure 97. Synthesis of compound **7**.

$LiAlH_4$ (0.4 mmol, 15.2 mg) was added dropwise to a solution of compound **3a** (0.2 mmol, 113.4 mg) in dry THF (4.0 mL) at 0 °C. The reaction mixture was stirred at room temperature and the progress of the reaction was monitored by TLC. Upon completion, the mixture was then concentrated and the residue was purified by chromatography on silica gel (eluent: petroleum ether/ethyl acetate) to afford the desired product **7** (92.2 mg, 81% yield, yellow oil). ^{1}H NMR (400 MHz, $CDCl_3$) δ 8.28 (s, 1H), 7.75 (d, *J* = 8.0 Hz, 2H), 7.47 (d, *J* = 7.9 Hz, 2H), 7.32 – 7.25 (m, 6H), 7.18 – 7.14 (m, 3H), 7.06 – 7.01 (m, 2H), 4.42 (d, *J* = 11.0 Hz, 1H), 4.21 – 4.16 (m, 1H), 3.89 – 3.81 (m, 1H), 3.09 – 3.04 (m, 2H), 2.39 (s, 3H), 2.04 (d, *J* = 11.9 Hz, 1H), 1.88 (d, *J* = 14.4 Hz, 1H), 1.64 (d, *J* = 12.7 Hz, 1H), 1.45 – 1.37 (m, 2H), 1.11 – 1.03 (m, 2H), 0.72 – 0.62 (m, 1H); ^{13}C NMR (100 MHz, $CDCl_3$) δ 153.6, 144.4, 138.6, 136.6, 136.4, 134.4, 134.2, 129.7, 129.0, 128.5, 128.3, 127.5, 126.7, 126.4, 122.8, 118.7, 115.8, 55.5, 51.1, 50.6, 31.8, 31.5, 26.2, 24.0, 21.5; IR (neat): 3360 (br), 2924, 1682, 1661, 1647, 1593, 1532, 1489, 1443, 1339, 1221, 1165, 1088, 1041, 749,696, 665, 591; HRMS (ESI) m/z: $[M + H]^+$ calcd for $C_{31}H_{32}N_4NaO_3S$ 563.2087, found 563.2087.

(*E*)-1-benzylidene-4-formyl-*N*-phenyl-2-tosyl-1,2,3,3a,4,5a,6,7,8,9-decahydro-5*H*-pyrrolo[3,4-*c*]cinnoline-5-carboxamide (8)

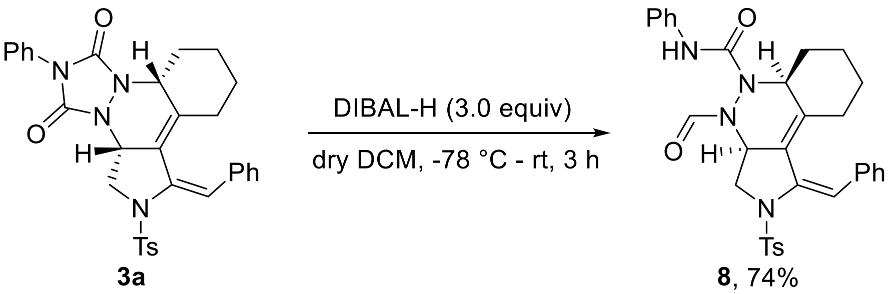

Supplementary Figure 98. Synthesis of compound **8**.

DIBAL-H (0.6 mmol, 0.6 mL, 1.0 M) was added dropwise to a solution of compound **3a** (0.2 mmol, 113.4 mg) in dry DCM (4.0 mL) at -78 ^{o}C. The reaction mixture was stirred at -78 ^{o}C and the progress of the reaction was monitored by TLC. Upon completion, the mixture was then concentrated and the residue was purified by chromatography on silica gel (eluent: petroleum ether/ethyl acetate) to afford the desired product **8** (84.2 mg, 74% yield) White solid (mp 154-156 °C). ^{1}H NMR (400 MHz, $CDCl_3$) δ 8.14 (s, 1H), 7.68 (d, *J* = 7.7 Hz, 2H), 7.61 (s, 1H), 7.45 (d, *J* = 8.1 Hz, 2H), 7.36 – 7.32 (m, 2H), 7.26 – 7.23 (m, 5H), 7.17 – 7.13 (m, 3H), 7.08 (s, 1H), 4.76 – 4.69 (m, 2H), 4.27 (t, *J* = 8.0 Hz, 1H), 3.60 (t, *J* = 10.7 Hz, 1H), 2.33 (s, 3H), 2.00 (d, *J* = 9.9 Hz, 1H), 1.88 (d, *J* = 13.4 Hz, 1H), 1.67 (d, *J* = 11.7 Hz, 1H), 1.40 (d, *J* = 11.3 Hz, 2H), 1.27 – 1.23 (m, 1H), 1.11 (t, *J* = 13.1 Hz, 1H), 0.54 – 0.45 (m, 1H); ^{13}C NMR (100 MHz, $CDCl_3$) δ 164.5, 154.7, 144.5, 137.4, 137.3, 136.2, 134.1, 132.3, 129.6, 128.9, 128.5, 128.0, 127.5, 126.7, 124.5, 122.7, 121.0, 114.7, 54.4, 53.3, 52.0, 32.7, 31.6, 26.2, 24.1, 21.4; IR (neat): 3348 (br), 2930, 1680, 1597, 1532, 1445, 1353, 1236, 1165, 1088, 984, 812, 751, 695, 666, 588, 547; HRMS (ESI) m/z: $[M + Na]^+$ calcd for $C_{32}H_{32}N_4NaO_4S$ 591.2036, found 591.2038.

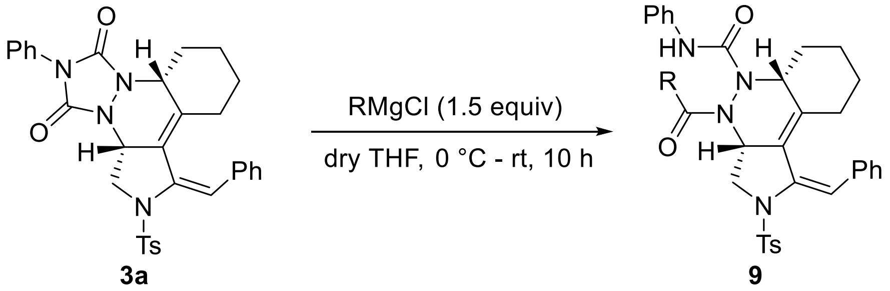

Supplementary Figure 99. Synthesis of compounds **9**.

(*E*)-4-benzoyl-1-benzylidene-*N*-phenyl-2-tosyl-1,2,3,3a,4,5a,6,7,8,9-decahydro-5*H*-pyrrolo[3,4-*c*]cinnoline-5-carboxamide (9a)

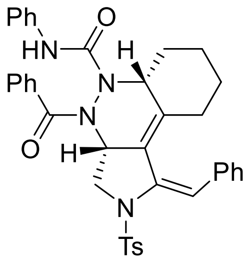

9a

PhMgCl (0.3 mmol, 0.15 mL, 2.0 M) was added dropwise to a solution of compound **3a** (0.2 mmol, 113.4 mg) in dry THF (4.0 mL) at 0 °C. The reaction mixture was stirred at 0 °C and the progress of the reaction was monitored by TLC. Upon completion, the mixture was then concentrated and the residue was purified by chromatography on silica gel (eluent: petroleum ether/ethyl acetate) to afford the desired product **9a** (127.7 mg, 99% yield, yellow oil). ^{1}H NMR (400 MHz, $CDCl_3$) δ 7.78 (d, J = 8.1 Hz, 2H), 7.56 (d, J = 7.6 Hz, 2H), 7.42 (t, J = 7.3 Hz, 1H), 7.36 – 7.26 (m, 6H), 7.22 – 7.07 (m, 9H), 6.71 (s, 1H), 4.93 – 4.89 (m, 1H), 4.46 – 4.38 (m, 2H), 3.44 (t, J = 10.4 Hz, 1H), 2.36 (s, 3H), 2.02 (d, J = 15.9 Hz, 1H), 1.84 (d, J = 9.4 Hz, 1H), 1.70 – 1.64 (m, 1H), 1.50 – 1.41 (m, 2H), 1.33 – 1.26 (m, 1H), 1.08 (t, J = 13.4 Hz, 1H), 0.68 – 0.59 (m, 1H); ^{13}C NMR (100 MHz, $CDCl_3$) δ 173.2, 155.5, 144.3, 137.1, 137.0, 136.6, 134.6, 133.7, 133.1, 130.9, 129.7, 129.1, 128.5, 128.1, 128.0, 127.8, 127.5, 126.5, 124.7, 123.3, 120.9, 113.5, 57.9, 54.1, 53.8, 33.3, 31.4, 26.3, 24.6, 21.5; IR (neat): 3419 (br), 2928, 1690, 1647, 1601, 1533, 1446, 1362, 1347, 1243, 1165, 1110, 924, 751, 666, 590, 545; HRMS (ESI) m/z: [M + Na]$^+$ calcd for $C_{38}H_{36}N_4NaO_4S$ 667.2349, found 667.2348.

(*E*)-4-acetyl-1-benzylidene-*N*-phenyl-2-tosyl-1,2,3,3a,4,5a,6,7,8,9-decahydro-5*H*-pyrrolo[3,4-*c*]cinnoline-5-carboxamide (9b)

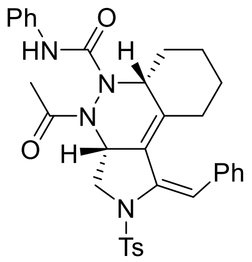

9b

CH_3MgCl (0.3 mmol, 0.1 mL, 3.0 M) was added dropwise to a solution of compound **3a** (0.2 mmol, 113.4 mg) in dry THF (4.0 mL) at 0 °C. The reaction mixture was stirred at 0 °C and the progress of the reaction was monitored by TLC. Upon completion, the mixture was then concentrated and the residue was purified by chromatography on silica gel (eluent: petroleum ether/ethyl acetate) to afford the desired product **9b** (67.6 mg, 58% yield) Yellow solid (mp 188-190 °C). ^1H NMR (400 MHz, $CDCl_3$) δ 7.76 (d, *J* = 8.0 Hz, 2H), 7.40 – 7.33 (m, 4H), 7.27 – 7.22 (m, 4H), 7.17 – 7.08 (m, 5H), 6.86 (s, 1H), 4.89 – 4.84 (m, 1H), 4.74 (d, *J* = 10.4 Hz, 1H), 4.19 (t, *J* = 8.5 Hz, 1H), 3.40 (t, *J* = 10.7 Hz, 1H), 2.34 (s, 3H), 2.15 (s, 3H), 2.10 (d, *J* = 9.5 Hz, 1H), 1.94 (d, *J* = 13.9 Hz, 1H), 1.75 – 1.69 (m, 1H), 1.44 – 1.26 (m, 3H), 1.11 (t, *J* = 13.3 Hz, 1H), 0.59 – 0.50 (m, 1H); ^{13}C NMR (100 MHz, $CDCl_3$) δ 173.1, 154.2, 144.4, 137.5, 137.1, 136.5, 134.5, 132.7, 129.6, 129.1, 128.5, 128.0, 127.5, 126.6, 124.7, 123.3, 120.7, 114.0, 55.5, 53.7, 53.5, 32.9, 31.5, 26.4, 24.4, 21.5, 21.0; IR (neat): 3324 (br), 2935, 1701, 1654, 1598, 1498, 1443, 1348, 1220, 1164, 1115, 1090, 924, 749, 663, 587, 547, 406; HRMS (ESI) m/z: $[M + Na]^+$ calcd for $C_{33}H_{34}N_4NaO_4S$ 605.2193, found 605.2194.

***N*-((1,3-dioxo-2-phenyl-6-(2-phenylacetyl)-2,3,5,7,8,9,10,10a-octahydro-1*H*-[1,2,4]triazolo[1,2-*a*]cinnolin-5-yl)methyl)-4-methylbenzenesulfonamide (10)**

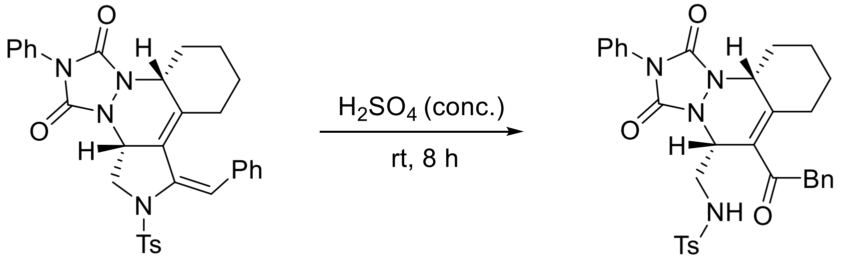

3a **10**, 93%

Supplementary Figure 100. Synthesis of compound **10**.

H_2SO_4 (98%, 8.0 mL) was added to a flame dried *Schlenk*-flask containing **3a** (0.2 mmol, 113.4 mg) at room temperature. The reaction mixture was stirred at room temperature and the progress of the reaction was monitored by TLC. The reaction took 8 h. Upon completion, the solution was diluted with 20 mL EtOAc and neutralized with saturated Na_2CO_3 at 0 °C. The aqueous phase was extracted with EtOAc three times and the combined organic phase was dried with Mg_2SO_4. The solvent was removed under reduced pressure and the residue was purified by chromatography on silica gel (eluent: petroleum ether/ethyl acetate) to afford the desired product **10** (108.8 mg, 93% yield) White solid (mp 174-176 °C). ^1H NMR (400 MHz, $CDCl_3$) δ 7.63 (d, *J* = 8.0 Hz, 2H), 7.50 (d, *J* = 8.1 Hz, 2H), 7.43 – 7.39 (m, 2H), 7.35 (d, *J* = 7.6 Hz, 1H), 7.31 – 7.17 (m, 8H), 5.55 (t, *J* = 6.7 Hz, 1H), 5.12 – 5.03 (m, 1H), 4.12 – 4.09 (m, 1H), 4.02 – 3.89 (m, 2H), 3.23 – 3.17 (m, 1H), 3.10 – 3.00 (m, 2H), 2.76 (d, *J* = 13.4 Hz, 1H), 2.36 (s, 3H), 1.93 – 1.84 (m, 2H), 1.75 – 1.50 (m, 3H); ^{13}C NMR (100 MHz, $CDCl_3$) δ 201.4, 152.9, 150.4, 143.4, 142.4, 136.8, 132.8, 131.1, 129.7, 129.6, 129.0, 128.8, 128.2, 127.6, 127.4, 126.9, 125.9, 58.5, 52.3, 49.7, 44.3, 34.6, 30.7, 27.5, 23.6, 21.4; IR (neat): 3268 (br), 2929, 1768, 1701, 1596, 1502, 1421, 1369, 1333, 1243, 1161, 1094, 1045, 814, 731, 659, 551; HRMS (ESI) m/z: $[M + Na]^+$ calcd for $C_{32}H_{32}N_4NaO_5S$ 607.1986, found 607.1989.

4-ethyl-7,10-diphenyl-4,10-dihydro-1*H*,6*H*-pyrrolo[2,3-*d*][1,2,4]triazolo[1,2-*a*]pyridazine-6,8(7*H*)-dione (11)

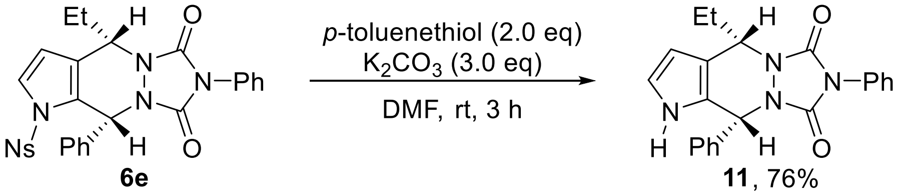

Supplementary Figure 101. Synthesis of compound **11**.

p-toluenethiol (0.4 mmol, 49.6 mg) and K_2CO_3 (0.6 mmol, 82.8 mg) were added in this order to the **6e** (0.2 mmol, 111.6 mg) in DMF (2.0 mL) at room temperature. The reaction mixture was stirred at room temperature and the progress of the reaction was monitored by TLC. Upon completion, the mixture was diluted with 10 mL DCM and washed with

water. The aqueous phase was extracted with DCM three times and the combined organic phase was dried with Mg_2SO_4. The solvent was removed under reduced pressure and the residue was purified by chromatography on silica gel (eluent: petroleum ether/ethyl acetate) to afford the desired product **11** (56.5 mg, 76% yield, yellow oil).[6] ^{1}H NMR (400 MHz, $CDCl_3$) δ 7.82 (s, 1H), 7.52 – 7.47 (m, 2H), 7.43 – 7.30 (m, 8H), 6.73 (t, *J* = 2.6 Hz, 1H), 6.14 (t, *J* = 2.5 Hz, 1H), 5.98 (s, 1H), 5.14 – 5.12 (m, 1H), 2.41 – 2.32 (m, 1H), 2.21 – 2.13 (m, 1H), 1.09 (t, *J* = 7.4 Hz, 3H); ^{13}C NMR (100 MHz, $CDCl_3$) δ 152.6, 151.6, 137.8, 131.3, 128.9, 128.8, 128.5, 127.9, 125.4, 123.2, 119.1, 116.3, 105.3, 58.5, 56.2, 27.4, 9.2; IR (neat): 3339(br), 2921, 2850, 1763, 1703, 1657, 1600, 1502, 1424, 1368, 1143, 1075,1028; HRMS (ESI) m/z: $[M + Na]^+$ calcd for $C_{22}H_{20}N_4NaO_2$ 395.1478, found 395.1479.

Deuterium Labeling Experiment

[D4]-**1a** (91% D)

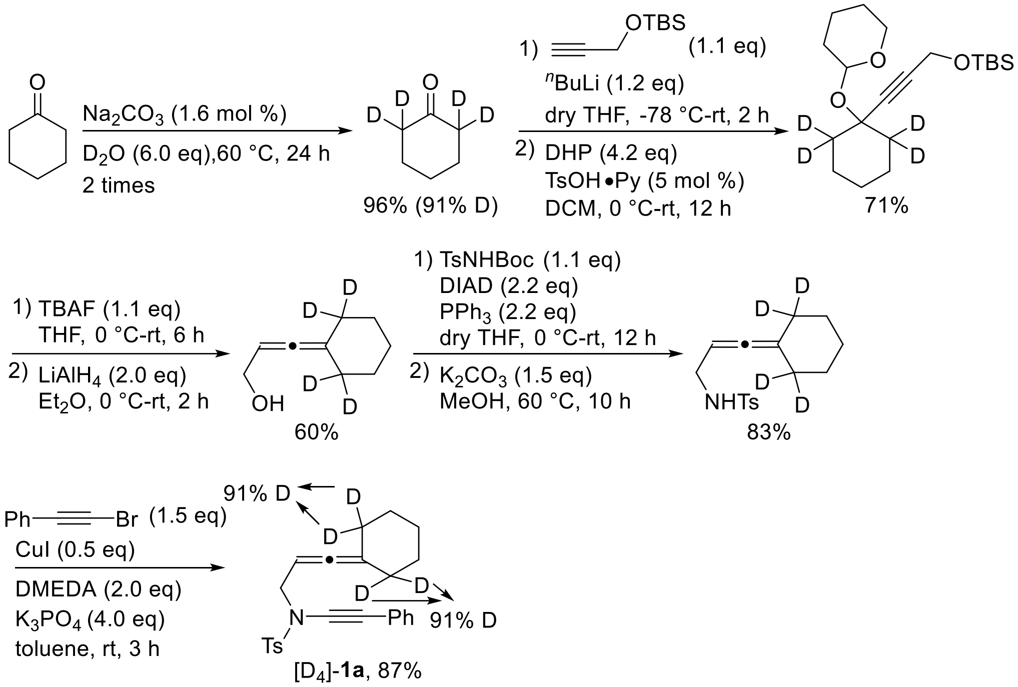

Supplementary Figure 102. Synthesis of compound [D4]-**1a**.

Compound [D4]-**1a** was prepared according to the known procedure[7,8]. ^{1}H NMR (400 MHz, $CDCl_3$) δ 7.84 (d, *J* = 8.3 Hz, 2H), 7.36 – 7.32 (m, 4H), 7.28 – 7.24 (m, 3H), 4.96 (t, *J* = 6.9 Hz, 1H), 4.01 (d, *J* = 6.9 Hz, 2H), 2.42 (s, 3H), 2.04 – 1.98 (m, 0.37H), 1.51 – 1.43 (m, 6H); ^{13}C NMR (100 MHz, $CDCl_3$) δ 200.6, 144.5, 134.9, 131.2, 129.6, 128.1, 127.6, 127.5, 122.9, 104.4, 83.1, 82.3, 70.9, 52.1, 26.9, 26.8, 25.7, 21.5.

[D4]-**3a** (91% D)

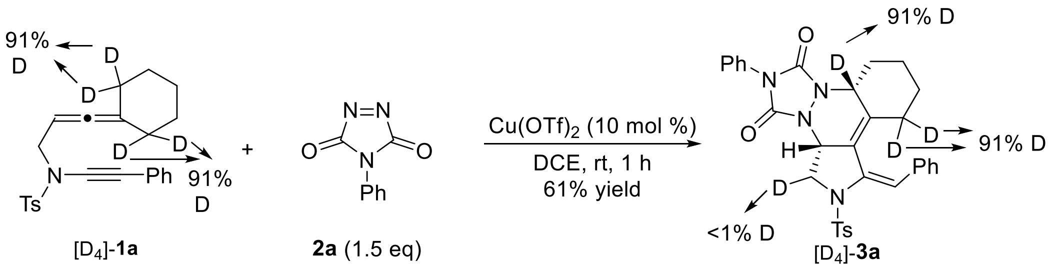

Supplementary Figure 103. Synthesis of compound [D4]-**3a**.

^{1}H NMR (400 MHz, $CDCl_3$) δ 7.79 (d, *J* = 7.1 Hz, 2H), 7.49 – 7.45 (m, 4H), 7.40 – 7.36 (m, 1H), 7.30 – 7.26 (m, 4H), 7.23 – 7.19 (m, 3H), 4.64 (t, *J* = 9.1 Hz, 1H), 4.19 – 4.15

(m, 1.09H), 3.49 (t, J = 9.9 Hz, 1H), 2.39 (s, 3H), 2.30 (d, J = 11.2 Hz, 1H), 2.04 (d, J = 2.0 Hz, 0.09H), 1.64 (d, 1H), 1.45 (d, J = 13.3 Hz, 1H), 1.38 – 1.31 (m, 2.09H), 1.21 – 1.15 (m, 1H), 0.61 (t, J = 12.3 Hz, 1H); ^{13}C NMR (100 MHz, $CDCl_3$) δ 154.6, 150.3, 144.5, 136.5, 134.5, 134.2, 132.9, 130.8, 129.7, 129.2, 128.6, 128.3, 128.1, 127.6, 126.9, 125.2, 122.5, 115.4, 54.6, 53.4, 30.4, 26.4, 23.6, 21.5.

[D_4]-**4a** (91% D)

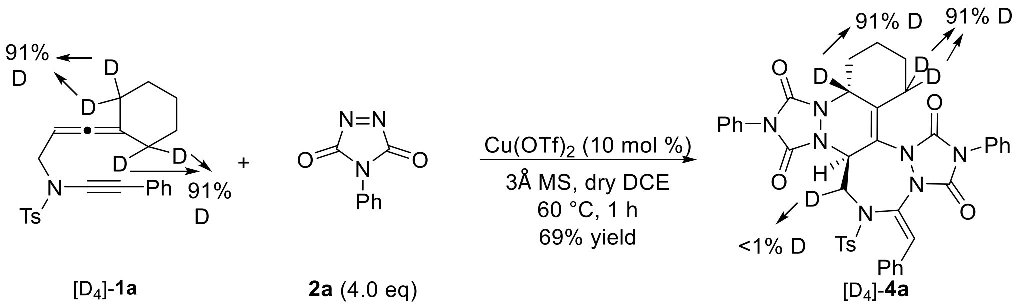

Supplementary Figure 104. Synthesis of compound [D_4]-**4a**.

^{1}H NMR (400 MHz, $CDCl_3$) δ 7.65 – 7.61 (m, 4H), 7.58 – 7.54 (m, 2H), 7.52 – 7.47 (m, 3H), 7.44 – 7.37 (m, 2H), 7.16 – 7.14 (m, 1H), 7.10 – 7.06 (m, 4H), 6.97 (d, J = 8.0 Hz, 2H), 6.73 (s, 1H), 5.05 – 5.01 (m, 1H), 4.85 – 4.81 (m, 1H), 4.48 – 4.44 (m, 0.09H), 3.09 – 3.03 (m, 1H), 2.64 (s, 0.09H), 2.48 (d, J = 11.1 Hz, 1H), 2.21 (s, 3H), 2.04 – 1.94 (m, 2.09H), 1.73 – 1.62 (m, 4H); ^{13}C NMR (100 MHz, $CDCl_3$) δ 151.2, 151.0, 150.7, 147.5, 146.7, 144.5, 135.8, 131.4, 131.4, 130.9, 130.8, 129.5, 129.2, 129.2, 129.1, 128.6, 128.5, 128.4, 128.3, 127.5, 127.5, 126.3, 126.1, 125.6, 125.4, 125.0, 118.6, 54.6, 49.8, 30.3, 27.0, 24.3, 21.4.

3a/[D_4]-**3a** (91% D)

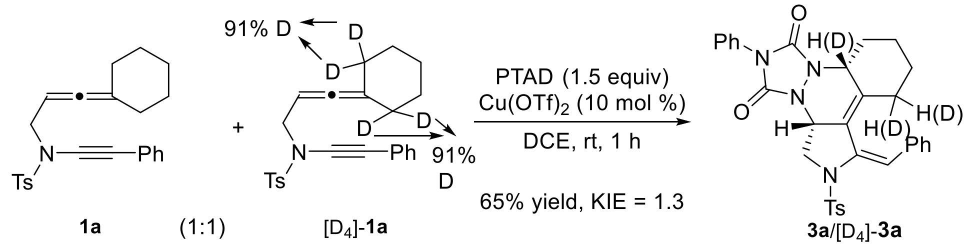

Supplementary Figure 105. Synthesis of compounds **3a**/[D_4]-**3a**.

^{1}H NMR (400 MHz, $CDCl_3$) δ 7.79 (d, J = 8.0 Hz, 2H), 7.49 – 7.45 (m, 4H), 7.40 – 7.37 (m, 1H), 7.30 – 7.26 (m, 4H), 7.23 – 7.19 (m, 3H), 4.67 – 4.62 (m, 1H), 4.23 – 4.15 (m,

1.63H), 3.49 (t, J = 10.0 Hz, 1H), 2.39 (s, 3H), 2.30 (d, J = 11.4 Hz, 1H), 2.04 (d, J = 13.8 Hz, 0.63H), 1.66 (d, J = 13.5 Hz, 1H), 1.45 (d, J = 12.6 Hz, 1H), 1.38 – 1.31 (m, 2.63H), 1.21 – 1.15 (m, 1H), 0.68 – 0.58 (m, 1H); ^{13}C NMR (100 MHz, $CDCl_3$) δ 154.6, 150.3, 144.5, 136.5, 134.6, 134.2, 132.9, 130.8, 129.7, 129.2, 128.6, 128.3, 128.1, 127.6, 126.9, 125.2, 122.4, 115.4, 55.5, 54.6, 53.4, 31.3, 30.5, 26.6, 23.7, 21.5.

4a/[D_4]-**4a** (91% D)

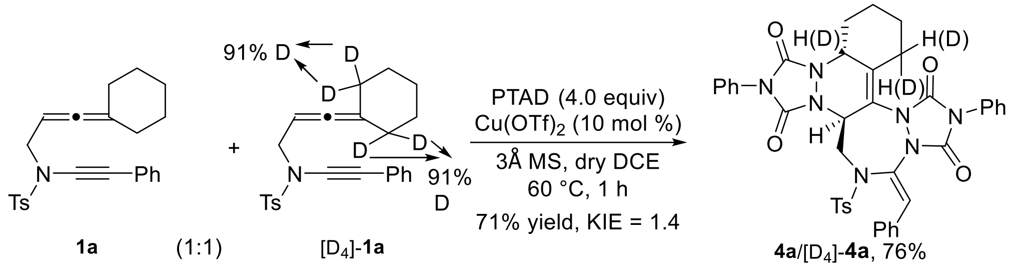

Supplementary Figure 106. Synthesis of compounds **4a**/[D_4]-**4a**.

^{1}H NMR (400 MHz, $CDCl_3$) δ 7.65 – 7.61 (m, 4H), 7.58 – 7.54 (m, 2H), 7.52 – 7.47 (m, 3H), 7.44 – 7.37 (m, 2H), 7.17 – 7.14 (m, 1H), 7.07 – 7.04 (m, 4H), 6.97 (d, J = 8.1 Hz, 2H), 6.73 (s, 1H), 5.05 – 5.01 (m, 1H), 4.86 – 4.81 (m, 1H), 4.48 – 4.44 (m, 0.58H), 3.09 – 3.03 (m, 1H), 2.67 (d, J = 13.6 Hz, 0.58H), 2.48 (d, J = 11.8 Hz, 1H), 2.22 (s, 3H), 2.00 – 1.95 (m, 2.58H), 1.75 – 1.62 (m, 4H); ^{13}C NMR (100 MHz, $CDCl_3$) δ 151.2, 151.0, 150.7, 147.5, 146.8, 144.5, 135.7, 131.4, 131.4, 130.9, 130.8, 129.5, 129.3, 129.2, 129.1, 128.6, 128.5, 128.4, 128.3, 127.5, 126.1, 125.6, 125.0, 118.6, 57.2, 54.6, 49.8, 30.5, 30.4, 27.2, 24.3, 21.4.

Supplementary References

(1) (a) A. Serafino, D. Balestri, L. Marchiò, M. Malacria, E. Derat, G. Maestri, *Org. Lett.* **2020**, *22*, 6354. (b) J. Kuang, S. Ma, *J. Org. Chem.* **2009**, *74*, 1763. (c) J. Kuang, S. Ma, *J. Am. Chem. Soc.* **2010**, *132*, 1786.

(2) W.-B. Shen, X.-T. Tang, T.-T. Zhang, S.-Y. Liu, J.-M. He, T.-F. Su, *Org. Lett.* **2020**, *22*, 6799.

(3) M. Zhu, M. H. Kim, S. Lee, S. J. Bae, S. H. Kim, S. B. Park, *J. Med. Chem.* **2010**, *53*, 8760.

(4) M. J. Bausch, B. David, *J. Org. Chem.* **1992**, *57*, 1118.

(5) (a) J. Li, S. R. Gilbertson, *Org. Lett.* **2021**, *23*, 2911. (b) J. Kuang, X. Tang, S. Ma, *Org. Chem. Front.* **2015**, *2*, 470. (c) Q. Liu, X. Tang, Y. Cai, S. Ma, *Org. Lett.* **2017**, *19*, 5174.

(6) A.-H. Zhou, Q. He, C. Shu, Y.-F. Yu, S. Liu, T. Zhao, W. Zhang, X. Lu, L.-W. Ye, *Chem. Sci.* **2015**, *6*, 1265.

(7) X.-Q. Zhu, P. Hong, Y.-X. Zheng, Y.-Y. Zhen, F.-L. Hong, X. Lu, L.-W. Ye, *Chem. Sci.* **2021**, *12*, 9466.

(8) S. Handa, D. J. Lippincott, D. H. Aue, B. H. Lipshutz, *Angew. Chem. Int. Ed.* **2014**, *53*, 10658.
