# Supplementary material for: Regioselective access to polycyclic N-heterocycles via homogeneous copper-catalyzed cascade cyclization of allenynes
Source: Commun Chem. 2023 May 31;6:104. doi: 10.1038/s42004-023-00910-9 (PMC10232412; doi:10.1038/s42004-023-00910-9)
Supplement: Supplementary file 2 — Description of Additional Supplementary Files [file 42004_2023_910_MOESM2_ESM.pdf]

Description of Additional Supplementary Files

File name: Supplementary Data 1

Description: NMR spectra.

File name: Supplementary Data 2

Description: the cif file of 3d.

File name: Supplementary Data 3

Description: the cif file of 4e.

File name: Supplementary Data 4

Description: the cif file of 6n.

File name: Supplementary Data 5

Description: the cif file of 8.

File name: Supplementary Data 6

Description: the cif file of 9b.

File name: Supplementary Data 7

Description: the cif file of 10.
